# T cell receptor and IL-2 signaling strength control memory CD8^+^ T cell functional fitness via chromatin remodeling

**DOI:** 10.1038/s41467-022-29718-2

**Published:** 2022-04-26

**Authors:** Shu Shien Chin, Erik Guillen, Laurent Chorro, Sooraj Achar, Karina Ng, Susanne Oberle, Francesca Alfei, Dietmar Zehn, Grégoire Altan-Bonnet, Fabien Delahaye, Grégoire Lauvau

**Affiliations:** 1 Albert Einstein College of Medicine, Department of Microbiology and Immunology, Bronx, NY 10461 USA; 2 National Cancer Institute, Cancer & Inflammation Program, Center for Cancer Research, ImmunoDynamics Group, Bethesda, MD 20892 USA; 3 Division of Animal Physiology and Immunology, School of Life Sciences Weihenstephan, Technical University of Munich, 85354 Freising, Germany; 4 Swiss Vaccine Research Institute, Epalinges, Switzerland and Division of Immunology and Allergy, Department of Medicine, Lausanne University Hospital, Lausanne, Switzerland; 5 Albert Einstein College of Medicine, Department of Genetics, Bronx, NY 10461 USA; 6 Institut Pasteur de Lille, UMR1283/8199, 59000 Lille, France

**Keywords:** Immunological memory, Cellular immunity, Gene regulation in immune cells, Lymphocyte activation

## Abstract

Cognate antigen signal controls CD8^+^ T cell priming, expansion size and effector versus memory cell fates, but it is not known if and how it modulates the functional features of memory CD8^+^ T cells. Here we show that the strength of T cell receptor (TCR) signaling controls the requirement for interleukin-2 (IL-2) signals to form a pool of memory CD8^+^ T cells that competitively re-expand upon secondary antigen encounter. Combining strong TCR and intact IL-2 signaling during priming synergistically induces genome-wide chromatin accessibility in regions targeting a wide breadth of biological processes, consistent with greater T cell functional fitness. Chromatin accessibility in promoters of genes encoding for stem cell, cell cycle and calcium-related proteins correlates with faster intracellular calcium accumulation, initiation of cell cycle and more robust expansion. High-dimensional flow-cytometry analysis of these T cells also highlights higher diversity of T cell subsets and phenotypes with T cells primed with stronger TCR and IL-2 stimulation than those primed with weaker strengths of TCR and/or IL-2 signals. These results formally show that epitope selection in vaccine design impacts memory CD8^+^ T cell epigenetic programming and function.

## Abstract (web summary)

The strength and duration of interleukin-2 (IL-2) signals is known to regulate T cell memory. Here the authors show that T cell receptor signalling strength controls the requirement for IL-2 signals to form CD8^+^ T cell memory with high functional activity and a wide range of genes with open chromatin regions.

## Introduction

Most currently approved vaccines in humans rely on strong antibody (Ab)-mediated immunity and only few trigger effective CD8^+^ T cell immunological memory^1^. CD8^+^ T cells can uniquely detect intracellular antigens and are equipped to directly kill microbial pathogen-infected and tumor cells. CD8^+^ T cells are also less prone to be impacted by point mutations that alter conformation sensitive Ab epitopes. Therefore, eliciting a pool of long-lived functional memory CD8^+^ T cells through rational design remains a necessity^2,3^.

Single-cell tracing studies have established that one CD8^+^ T cell has the potential to give rise to multiple progenies, supporting the idea that as T cells expand from an original clone, they differentially integrate priming signals, leading to distinct phenotypic fates^4,5^. While the initiation of clonal expansion most likely results from a digital on-off response, the functional fates of T cells are rather the consequence of an analog response to the sum of the different priming signals^6–9^. The current dogma states that the greater the T cell receptor (TCR) signals are, the stronger the primary expansion of CD8^+^ T cells is, and the more effector cells are produced at the expense of memory cells^10,11^. While cytokines, e.g., IL-2, IL-12, type I IFN, strongly skew CD8^+^ T cell-differentiation towards robust effector cells by directly enhancing expression of transcriptional regulators such as T-bet and Blimp-1 in the T cells^12–17^, cytokines can also directly potentiate TCR signaling^18^. When TCR signaling augments, the overall number of antigen-specific effector and memory CD8^+^ T cells that are generated also increases, but memory cells primed with different strengths of TCR signals do not differ in their ability to clonally re-expand in vivo^19^. Memory CD8^+^ T cells primed with weak TCR signals, however, fail to express effector functions in response to weak (but not strong) epitopes, suggesting that weak TCR signals during priming may induce functionally distinct memory cells^20^. Recent work investigating T cell epigenetic programming further established that the acquisition of functional effector or memory features by CD8^+^ T cells is achieved through specific histone modification-driven chromatin remodeling that occurs at early/effector stages of T cell differentiation^21–26^. In these studies, a significant overlap in the epigenetic landscapes between effector and memory CD8^+^ T cells has generally been reported. Although the memory CD8^+^ T cell epigenetic landscape is largely distinct from that of naïve T cells, memory cells also reacquire part of the naïve T cell-associated landscape via active demethylation of genes associated with naïve T cells status^23^.

Despite many studies investigating the process of CD8^+^ T cell priming and differentiation into memory cells, there is still a gap in knowledge as to whether the strength of TCR and cytokine signals modulate the functional features and the epigenetic programming of memory CD8^+^ T cells. In this work, we test the hypothesis that the strength of TCR signals regulates naïve CD8^+^ T cell dependency on IL-2 signals and their differentiation into functional memory cells. We next examine whether altering the strength of these signals individually or simultaneously modulates memory CD8^+^ T cell transcriptomic and epigenetic programming. We find that the strength of TCR signaling controls the need for IL-2 signals to form fully functional and epigenetically fit memory CD8^+^ T cells. CD8^+^ T cells that are primed under robust TCR and IL-2 signals form memory cells that competitively re-activate and re-expand, and have high stemness features.

## Results

### TCR signaling strength modulates early CD8^+^ T cell activation and transcriptional program

**Fig. 1:**
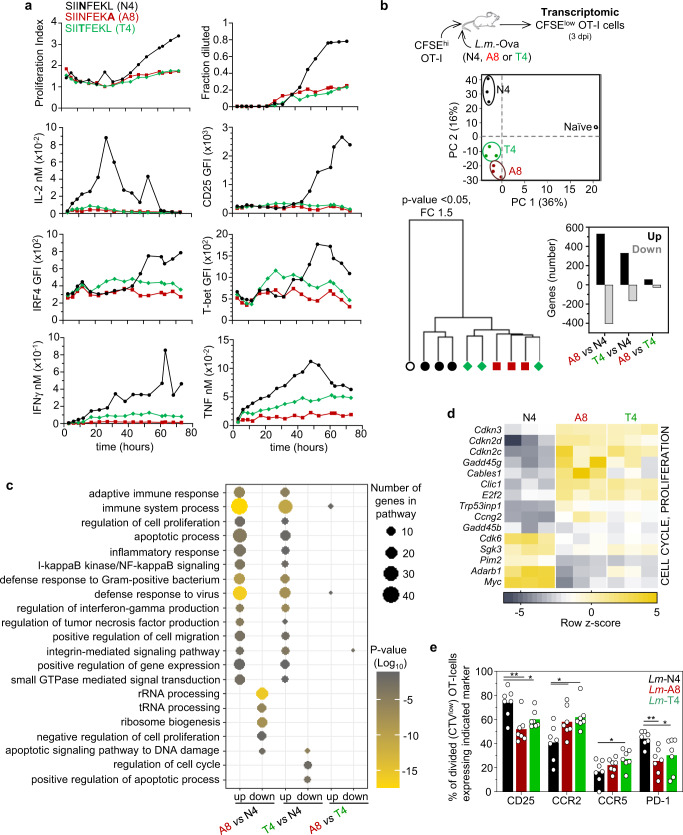
Impact of TCR signaling strength on early CD8^+^ T cell activation. **a** Naïve OT-I cells from spleens were stained with CFSE and stimulated in vitro with 10^−9^ or 10^−10 ^M of ovalbumin (Ova) peptide SIINFEKL (N4) or its altered peptide ligands (T4, A8) for quantitative multi-parameter kinetic monitoring. OT-I cells were stained for cell-surface CD25 and intracellular IRF4 and T-bet, and secreted IL-2, IFNγ and TNF were quantified as described. Each graph summarizes the extent of OT-I cell division (Proliferation index and Fraction diluted at 10^−10 ^M of peptides), OT-I cell expression levels (GFI) of stained markers or secreted cytokine levels (at 10^−9 ^M of peptides). **b** Naïve spleen-derived CFSE-labeled OT-I cells were adoptively transferred to recipient mice, subsequently infected with *Lm-*Ova N4, A8 or T4. 3 days later, CFSE^low^ OT-I cells were flow-sorted from spleens of 3 independent replicates of mice. Total RNA was extracted, reverse transcribed to cDNA before running an Affymetrix mouse expression arrays (Pico 1.0). Principal component analysis (PCA) and hierarchical clustering of expressed genes in analyzed groups with each symbol featuring one mouse. Bar graph shows significantly up- and downregulated genes, with fold change ±1.5 and *p*-value ≤ 0.05. **c** Network analysis of biological-process gene-ontology (GO) term enrichment among significantly up- or downregulated genes in the indicated priming comparisons. Differentially regulated genes were analyzed for over-represented GO terms on DAVID website and visualized in a scatterplot graph. Node color is proportional to the FDR-adjusted *p*-value of the enrichment. Node size is proportional to the number of genes in each GO term. For (B, C), one-way anova statistical test was used. **d** Heat map of cell-cycle/proliferation-related genes for which expression is significantly different between N4, A8 and T4 primed OT-I cells. Row *z*-score fold change is shown in heat map. **e** Proportion of CTV^low^ (divided) OT-I cells expressing indicated markers 3 days post immunization with *Lm-*Ova N4, A8 or T4 with *n* = 7 mice per group over 2 independent experiments. Each symbol represents 1 mouse and *p*-values are indicated when relevant with *p < 0.05 and **p < 0.01, using two-tailed unpaired Student’s *t* test corrected for multiple comparison using a 1% False Discovery Rate (FDR).

We investigated whether TCR signaling strength alters early naïve CD8^+^ T cell activation by using two altered peptide ligands (APLs) of the ovalbumin-derived K^b^-presented SIINFEKL epitope (Ova_257-264_ or N4) that both exhibit low potency for stimulating OT-I transgenic T cells compared to the natural N4 epitope. One variant (A8) decreases peptide/MHC stability after substituting the C-terminus leucine anchor residue with alanine while the other (T4) alters OT-I TCR/peptide interactions upon replacement of the asparagine by a threonine (Supplementary Fig. 1a). T4 and N4 both stabilize K^b^ similarly whereas A8 is a much weaker K^b^ stabilizer (by a factor of ~3–4, Supplementary Fig. 1b^19,27^). Quantitative multi-parameter kinetic monitoring of OT-I cells stimulated in vitro with N4, A8 and T4 peptides showed that the proportion of OT-I cells undergoing at least one division (Fraction diluted) and the extent of their expansion (Proliferation index) was substantially lower for OT-I cells that received weak TCR signals compared to those that were stimulated with strong TCR signals (Fig. 1a and Supplementary Fig. 1c). This was observed across a wide range of peptide concentrations (from 10^−6^ to 10^−9^ M) and as a result of low epitope/MHC stability (A8) or weak OT-I TCR interaction with its cognate epitope (T4). Moreover, such weakly stimulated (A8, T4) OT-I cells secreted little to no cytokines (IL-2, IFNγ, TNF) and only expressed low levels of the Th1 master transcription factor T-bet. Overall, these in vitro data show a strong impact of the strength of TCR signals on CD8^+^ T cell proliferation/expansion, cytokine secretion and effector differentiation. Next, we investigated how naïve CD8^+^ T cells integrate the distinct levels of TCR signals in vivo by FACS-sorting activated/divided (CFSE^low^) OT-I cells 3 days post-infection with *Listeria monocytogenes (Lm)* expressing either A8 or T4 APLs, or the N4 epitope and conducted whole genome expression arrays (Fig. 1b and Supplementary Fig. 1d). Principal component analysis (PCA) shows that, as expected, OT-I cells primed with weak (A8, T4) or strong (N4) TCR signals segregated away from naïve counterparts. A8- and T4-primed OT-I cells clustered together but apart from N4-counterpart, in line with our in vitro quantitative analysis where A8- and T4-primed OT-I cells underwent similar phenotypic and functional changes. This result was also reflected in the large overlap of genes differentially expressed in A8- or T4- compared to N4-primed OT-I cells (~800 genes). GO pathway analysis of upregulated genes in OT-I cells primed with *Lm* expressing A8 or T4 compared to N4 showed an overlap in pathways related to the immune response (adaptive, inflammation, interferon and tumor necrosis factor, defense to virus/bacteria), cell cycle/proliferation and death, adhesion and migration (Fig. 1c, Supplementary Data 1). Differences were noted in the pathways related to the differentially downregulated genes but these pathways were mostly involved in non-immune functions. Essentially no pathways were found when comparing A8- and T4-primed OT-I cells, consistent with the very small number of differentially expressed genes between these two priming conditions. Among the differentially induced genes between A8 or T4- versus N4-primed OT-I cells, many encoded cell-cycle-cyclin-dependent kinases (*Cdkn3, Cdkn2d, Cdkn2c, Cables1, Cdk6*) and transcription factors (TFs) (*Myc, Irf4, Mxd1, E2f2*), interferon-related proteins (*Ifit1, Ifit2, Isg15, Oas1a, Oas3, Ddx58, Nod1*) and TFs (*Irf7, Stat2*), migration and chemotaxis (*Itgax, Sell, Ccr2, Ccr5, S1p1r, klf2*) and effector functions (*Gzma, Gzmk, Klrc1, Klrd1, Klrc2, Klrc3*) (Fig. 1d and Supplementary Fig. 1e). Several of these genes were upregulated in A8- and T4- compared to N4-primed OT-I cells, indicating that naïve CD8^+^ T cells receiving weak TCR signals overall incorporate greater signals related to the highlighted pathways. For instance, expression of several genes coding for proteins involved in cell cycle inhibition (*Cdkn3, Cdkn2d, Cdkn2c, Cables1, E2f2, Trp53inp1)* was increased in OT-I cells primed with the weak A8 and T4 APLs while others implicated in robust cell cycle progression were diminished (*Myc, Cdk6, Irf4*) compared to OT-I cells primed with the strong N4 epitope. Greater upregulation of the high affinity IL-2 receptor alpha chain CD25 on divided (CTV^low^) OT-I cells was also confirmed by FACS 3 days post *Lm*-N4 immunization (Fig. 1e), consistent with a prolonged proliferative program compared to A8- and T4-primed counterparts. Thus, T cell proliferation, a major readout outcome in OT-I cells primed with the N4, T4 or A8 epitope in vitro and in vivo seemed consistent. We also tested whether other differentially expressed genes in activated dividing OT-I cells translated into distinct protein expression levels 3 days post immunization (Fig. 1e and Supplementary Figs. 1f, 2a). We confirmed findings for CCR2, CCR5 and PD-1 which also reflected different activation states of primed T cells. However, expression of CD127, KLRD1 or CD11c was not significantly distinct possibly reflecting differences in the kinetics of gene versus protein expression. Collectively, modulating the amount of TCR signals incorporated by a naïve CD8^+^ T cell clone during priming in vitro and in vivo drives a clearly distinct but consistent functional and transcriptional activation program.

### TCR signaling strength establishes the requirement for IL-2-signals to form functional memory

**Fig. 2:**
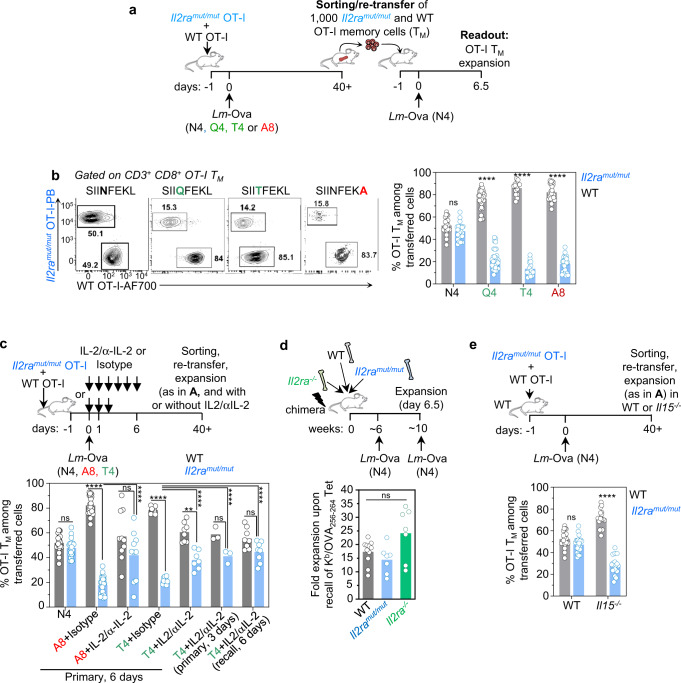
CD8^+^ T cells primed with weak TCR and IL-2 signals failed to form memory cells that competitively expand during recall infection. **a** Schematic of experimental design used or referred to in B, C, E. Briefly, 2000 *Il2ra*^*mut/mut*^ or WT OT-I cells expressing constitutive tomato (Td) and congenically distinct, were adoptively transferred to recipient mice, and infected the next day with *Lm*-Ova N4, Q4, T4 or A8. 1000 *Il2ra*^*mut/mut*^ and WT OT-I memory cells (~day 40 p.i.) were then sorted by FACS and transferred at a 1:1 ratio to new recipient mice subsequently infected with *Lm*-OvaN4 the next day. 6.5 days later, spleens were harvested and stained to quantify *Il2ra*^*mut/mut*^ and WT OT-I memory cell expansion. (**b**) Following (A), representative FACS dot plots of re-expanded *Il2ra*^*mut/mut*^ and WT OT-I memory cells originally primed with the indicated Ova APLs are shown. Bar graph represents the pooled frequencies of re-expanded *Il2ra*^*mut/mut*^ versus WT OT-I memory cells across >6 independent replicate experiments (*n* = 30 mice). **c** As in (A), but mice were also injected with IL-2/anti-IL2 mAb complexes during initial or recall infection, every day for 6 or 3 days as depicted on the schematic and in Supplementary Fig. 2b. The bar graph shows the relative average frequency of re-expanded *Il2ra*^*mut/mut*^ and WT OT*-*I memory cells across 1–4 independent replicate experiments (*n* = 3–30 mice). **d** WT/*Il2ra*^*mut/mut*^/*Il2ra*^*−/−*^ mixed BM chimeras (ratio 1:1:1) were infected i.v. with *Lm*-OvaN4 and challenged 4 weeks later with *Lm*-OvaN4. 6.5 days later, splenocytes were stained with Ova_257–264_/K^d^ tetramers (Tet^+^) and appropriate congenic markers. Bar graphs show the relative fold expansion of Tet^+^ CD8^+^ T cells across 2-3 independent replicate experiments (n = 11 mice). **e** As in (A), but FACS-sorted *Il2ra*^*mut/mut*^ and WT OT*-*I memory cells were adoptively transferred to either WT or *Il15*^*−/−*^ hosts. The bar graph shows the summary of the relative frequency of re-expanded *Il2ra*^*mut/mut*^ and WT OT*-*I memory cells across 4–5 replicate experiments (*n* = 17 mice for transfer into WT (*p* = 0.0907) and −24 mice for transfer in *Il15*^*−/−*^ hosts (*p* < 0.000001)). In all panels, each symbol represents 1 mouse and *p*-values are indicated when relevant with *p < 0.05; **p < 0.01; ***p < 0.001; ****p < 0.0001; ns, not significant, using two-tailed unpaired Student’s *t* test.

Since OT-I cells that received weak compared to strong TCR stimulation exhibited less sustained in vitro proliferation, and upregulated genes involved in shutting off cell cycle in vivo (Fig. 1), we further explored how altering IL-2 signaling, the key T cell proliferative signal, affected T cell clonal expansion and functional memory cell formation in the context of distinct strengths of TCR signals. We hypothesized that in situations of weak TCR stimulation and impaired IL-2 signaling, CD8^+^ T cells may not form functional memory cells. We used *Il2ra*^*mut/mut*^ OT-I cells, impaired in IL-2 signaling as a result of a point mutation in an evolutionarily conserved tyrosine (Y129)^28^. *Il2ra*^*mut/mut*^ OT-I cells were co-transferred with WT counterparts into recipient mice subsequently infected with *Lm* expressing distinct Ova APLs (Q4, T4 or A8) or the N4 epitope (Fig. 2a). As expected^19^, primary expansion of OT-I cells (day 7) was significantly reduced by lowering TCR signaling, and OT-I cells impaired in IL-2 signaling (*Il2ra*^*mut/mut*^) proliferated 3 times less than WT counterparts whether infected with any of the *Lm*-Ova APLs (Supplementary Fig. 2b). At memory stage, i.e., >40 days post primary *Lm* infection, we sorted and co-transferred the same number (1000) of *Il2ra*^*mut/mut*^ and WT OT-I memory cells to naïve mice, that were subsequently challenged with *Lm*-N4 to rigorously assess their ability to competitively expand 6.5 days later (Fig. 2a). While N4-primed *Il2ra*^*mut/mut*^ and WT OT-I memory cells underwent comparable proliferation, Q4-, T4- and A8-primed *Il2ra*^*mut/mut*^ OT-I memory cells did not and expanded ~4–5 times less than WT counterparts (Fig. 2b). Consistent with our hypothesis, this result indicated that TCR signaling strength establishes the dependency of naïve CD8^+^ T cells on intact IL-2 signaling for their differentiation into memory cells capable of competitive clonal expansion. Providing robust IL-2 signals to *Lm*-A8- and *Lm*-T4-primed *Il2ra*^*mut/mut*^ OT-I cells, using IL-2/anti-IL-2 monoclonal Ab (mAb) treatment for 6 days during primary infection, showed significant rescue of their ability to expand compared to control isotype Ab-treated counterparts during secondary antigen encounter after *Lm-*N4 challenge infection (Fig. 2c). IL-2-rescued *Il2ra*^*mut/mut*^ OT-I memory cells primed with the weak Ova APLs (A8, T4) expanded closely to WT OT-I memory cells. When IL-2 signals were given for only 3 instead of 6 days, the extent of the rescue was comparable, suggesting that IL-2 signals were mostly important at early stages post-priming. Interestingly, if IL-2 signals were only provided during the recall response, *Il2ra*^*mut/mut*^ OT-I memory cells primed with weak APL (here T4) expanded like WT counterparts, indicating that providing robust IL-2 signaling during Ag re-encounter also restores their ability to competitively expand (Fig. 2c and Supplementary Fig. 2c). To extend results to polyclonal memory CD8^+^ T cells, we next tracked *Il2ra*^*mut/mut*^, *Il2ra*^*−/−*^ and WT N4-primed polyclonal K^b^/Ova_254-261_ tetramer^+^ (Tet^+^) memory CD8^+^ T cells in mixed bone marrow (BM) chimera mice reconstituted with an equal ratio of each of the above BM genotype (Fig. 2d). We confirmed that antigen-specific memory CD8^+^ T cell of all genotypes proliferated independently of IL-2 upon secondary Ag encounter. However, when N4-primed *Il2ra*^*mut/mut*^ and WT OT-I memory cells were transferred to *Il15*^*−/−*^ mice, they failed to expand equivalently after challenge with either *Lm* or VSV expressing OvaN4, indicating that STAT5 signaling downstream of either IL-15 or IL-2 cytokine stimulation is essential for maximal clonal memory cell expansion (Fig. 2e and Supplementary Fig. 2d, e). In summary, these results establish that the strength of TCR signaling regulates CD8^+^ T cell-dependency on IL-2 signals in order to form memory cells that can effectively expand during secondary Ag encounter. The data further suggest that robust IL-2 signals are mostly required early on post CD8^+^ T cell priming, but that IL-2 signals provided during the recall response can also rescue memory CD8^+^ T cell competitive expansion.

### Existence of IL-2-dependent and IL-2-independent natural epitopes to form functional memory

**Fig. 3:**
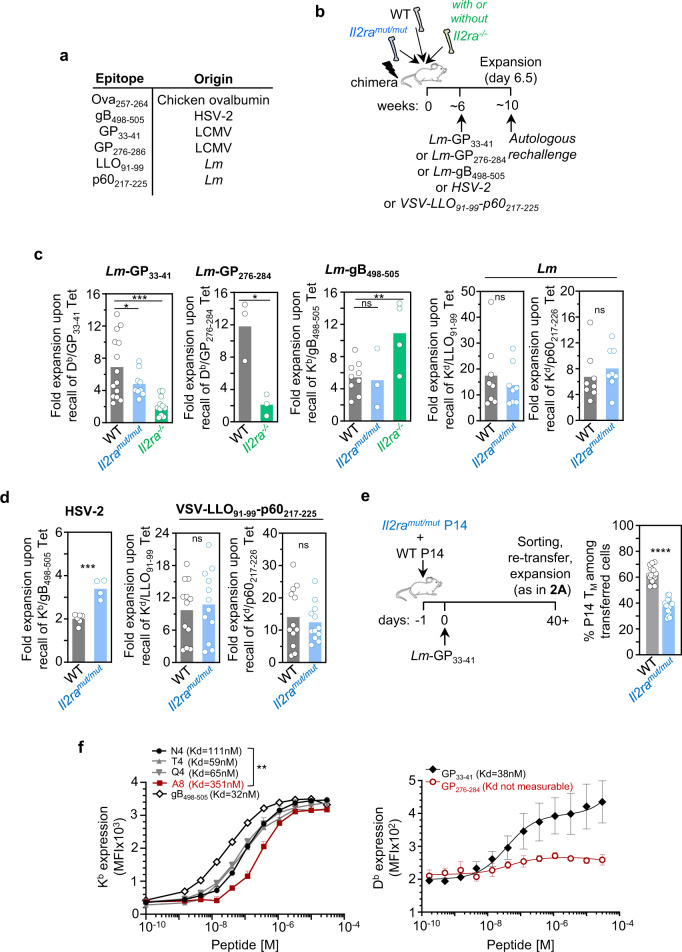
Several natural epitopes require IL-2 signaling to form functional memory CD8^+^ T cells. **a** Table listing the naturally presented epitopes used in our studies and their microbial pathogen origin. **b** Schematic of experimental design for experiments in C, D. **c** WT/*Il2ra*^*mut/mut*^ or WT/*Il2ra*^*mut/mut*^/*Il2ra*^*−/−*^ mixed BM chimeras (ratio 1:1:1) were infected i.v. with indicated pathogens 6–8 weeks post-reconstitution, and challenged with the same autologous pathogen 4–5 weeks after before staining for FACS analysis 6.5 days later using indicated MHC class I tetramers (Tet^+^) and appropriate congenic markers. Bar graphs show the relative fold expansion of Tet^+^ CD8^+^ T cells across 2 replicate experiments (*n* = 8–15 mice). **d** WT/*Il2ra*^*mut/mut*^ mixed BM chimeras were set up as in (B), infected and then challenged 4 weeks later with indicated pathogens before staining spleen cells for FACS analysis 4 or 6.5 days later. Bar graphs show the relative fold expansion of Tet^+^ CD8^+^ T cells across 2 replicate experiments (*n* = 5–12 mice). **e** *Il2ra*^*mut/mut*^ and WT P14 cells were adoptively transferred to naive hosts, subsequently infected the next day with *Lm*-GP_33–41_. *Il2ra*^*mut/mut*^ and WT P14 memory cells were sorted 4 weeks later, transferred (ratio 1:1) to naive hosts, and challenged the next day with *Lm*-GP_33-41_. Spleens cells were stained for FACS analysis 6.5 days later. Bar graph shows the relative frequency of *Il2ra*^*mut/mut*^ and WT P14 memory cells across 3 replicate experiments (*n* = 15 mice, *p* < 0.00001). Each symbol (**c**–**e**) represents one mouse and *p*-values are indicated when relevant with *p < 0.05; **p < 0.01; ***p < 0.001; ****p < 0.0001; ns, not significant, using two-tailed paired Student’s *t* test. **f** H2-K^b^ and H2-D^b^ RMA-S stabilization assay by indicated peptides. Data are presented as average MFI values and error bars indicate SEM. Kd values represent the peptide concentration required to achieve half of maximum cell-surface K^b^ or D^b^ expression. Data represent 1 of 3 independent replicate experiments. *P*-value is calculated using two-tailed unpaired Student’s *t* test on Kb MFI at 120 nM of peptide (*p* = 0.005287).

We next investigated whether naturally presented epitopes may also require IL-2 signaling during priming in order to form functional memory cells that competitively expand during a recall infection. We selected five epitopes expressed in *Lm*, all originally derived from three distinct microbial pathogens, the lymphochoriomeningitis virus (LCMV, GP_33–41_ and GP_276–284_), the herpes simplex virus 2 (HSV-2, gB_498–505_) and *Lm* (LLO_91–99_ and p60_217–225_)(Fig. 3a). We first generated mixed bone marrow (BM) chimeras in lethally irradiated WT recipient mice reconstituted with BM from either *Il2ra*^*mut/mut*^, *Il2ra*^*−/−*^ and WT mice or from *Il2ra*^*mut/mut*^ and WT mice. We used BM donor and recipient mice that express i) distinct CD45 congenic marker combinations on the C57BL/6 genetic background (H-2^b^) and ii) the K^d^ molecule (B6-K^d^). Fully reconstituted chimeras were next primary infected and further challenged with the same microbial pathogen (autologous) 4–8 weeks later (Fig. 3b). Fold expansion of polyclonal Tet^+^ memory CD8^+^ T cells of each genotype and specific for each listed epitope was then quantified by comparing the average splenic frequencies of Tet^+^ memory cells prior to secondary challenge to those 6.5 days post challenge (Fig. 3c, d). While *Il2ra*^*−/−*^ or *Il2ra*^*mut/mut*^ GP_33–41_/D^b^ and GP_276–284_/D^b^-specific memory CD8^+^ T cells failed to expand as competitively as WT counterparts, and required robust IL-2 signals during priming for competitive secondary expansion, those recognizing gB_498–505_/K^b^, LLO_91–99_/K^d^ and p60_217–225_/K^d^ did not (Fig. 3c). Next, using HSV-2 or VSV-expressing the *Lm*-derived LLO_91–99_ and p60_217–225_ epitopes to infect and challenge *Il2ra*^*mut/mut*^/WT mixed BM chimeras, we found that these epitopes also did not depend on IL-2 to form functional memory cells, similarly to when they were expressed in *Lm* (Fig. 3d). Using the clonal population of GP_33–41_/D^b^-specific *Il2ra*^*mut/mut*^ and WT P14 TCR transgenic memory CD8^+^ T cells primed upon *Lm*-GP_33–41_ infection, we further confirmed that P14 memory CD8^+^ T cells also failed to competitively expand when IL-2 signals were impaired (Fig. 3e). Notably, during primary infection of the chimera mice, some epitopes (Ova_257–264_, gB_498–505_) but not others (GP_33–41_) required IL-2 signals for optimal expansion, whether expressed in *Lm* or by other microbial pathogens (VSV, HSV-2)(Supplementary Fig. 3b–g). However, the dependency on IL-2 signals for maximal primary expansion did not predict that of the recall response. Since these various microbial pathogen infections are likely to induce a very distinct inflammatory environment in infected hosts, this result further suggested that IL-2 dependency for formation of functional memory is more likely to be epitope-driven rather than inflammation-driven. The GP_276–286_ and the A8 epitopes failed to respectively form stable complexes with D^b^ and K^b^ in an in vitro RMA-S stabilization assay, while GP_33–41_ (D^b^), gB_498–505_ (K^b^) and N4 (K^b^) epitopes form stable MHC class I complexes (Fig. 3f). Thus, while it is likely that epitopes that cannot form stable complexes with their respective MHC may only trigger weak TCR signaling, and be dependent on IL-2 signals to induce functional memory cells, other mechanisms where epitopes only provide weak signals to T cells (like T4 and Q4 APLs) are also involved. Taken together, these results suggest that the amount of TCR signal an epitope gives to naïve polyclonal CD8^+^ T cells, but not a specific set of T cell clones or inflammatory factors elicited by distinct microbial pathogen infections, will most likely establish the functional fates of memory CD8^+^ T cells and their dependency on cytokine signals. These data also indicate that natural epitopes can trigger distinct memory CD8^+^ T cell programs that either do or do not require intact IL-2 signals to form a pool of fully functional memory cells.

### The duration of cognate T cell antigen presentation may not dictate the formation of functional memory

**Fig. 4:**
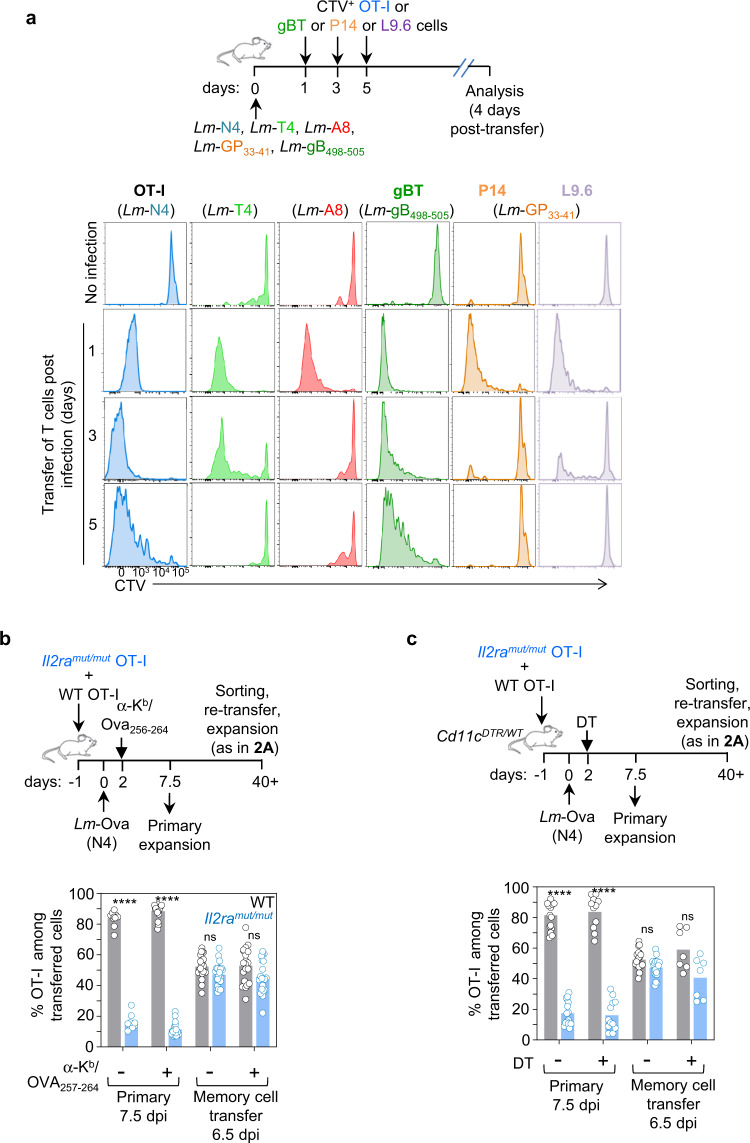
The duration of cognate antigen presentation and the formation of functional memory CD8^+^ T cells are uncoupled. **a** WT mice were infected with indicated *Lm* strains. At specified times, OT-I, gBT, P14 or L9.6 cells were labeled ex vivo with CTV and adoptively transferred to previously infected hosts. Spleen cells were stained for FACS analysis 4 days later. Representative FACS histograms show the dilution of CTV staining in transferred T cells. **b** *Il2ra*^*mut/mut*^ and WT OT-I cells adoptively transferred to naïve WT mice were subsequently infected the day after with *Lm*-Ova N4. Antigen presentation was disrupted by injecting anti-MHC-I K^b^/Ova_256–264_ mAb 48 h post-infection and spleen cells were stained for FACS analysis either 7.5 days later or 6.5 days after re-transfer of sorted memory cells (as in Fig. 2a). Bar graph shows the relative frequency of *Il2ra*^*mut/mut*^ versus WT OT-I cell expansion in 2–5 replicate experiments (*n* = 10–24 mice). **c** Same design as in (**b**), but with *Il2ra*^*mut/mut*^ and WT OT-I cells adoptively transferred to CD11c^DTR/WT^ recipient mice further injected with diphtheria toxin (DT) 48 h post-infection to deplete CD11c^+^ cells. The bar graph shows the relative frequency of *Il2ra*^*mut/mut*^ versus WT OT-I cell expansion across 2–4 independent replicate experiments (*n* = 7–16 mice; each symbol represents one mouse).

We explored the relationship between the duration of epitope presentation in vivo and the induction of functional memory CD8^+^ T cells (Fig. 4a). We adoptively transferred CTV-labeled OT-I, P14, L9.6 (p60_217–225_/K^d^-specific) and gBT-I (gB_498–505_/K^b^-specific) CD8^+^ T cells in mice infected with *Lm* expressing N4, T4, A8 or either of the other epitopes 1, 3 or 5 days before. While both Ova (N4)- and gB_498–505_-derived epitopes could respectively prime naïve OT-I and gBT-I T cells and induce their robust proliferation for at least five days in vivo, *Lm*-A8, LCMV-GP_33–41_- and *Lm* p60_217–225_-derived epitopes only induced OT-I, P14 and L9.6 T cell proliferation for ~2 days, respectively. Since CD8^+^ T cells specific for Ova(N4)-, gB- and p60-derived epitopes did not require IL-2 signals to form functional memory but those specific for GP_33–41_ or induced by A8 did (Fig. 3c, d), it further suggested that the length of epitope presentation in vivo and the formation of functional memory cells were uncoupled. Consistent with this finding, OT-I memory cells could be primed by the weak T4 APL for at least 3 days, although robust IL-2 signals were required to form functional memory cells.

We next tested if disrupting epitope presentation 2 days after priming prevented the induction of functional memory cells. Mice were co-transferred with *Il2ra*^*mut/mut*^ and WT OT-I cells and either treated with an Ova_257–264_/K^b^ blocking mAb (WT mice) or selectively depleted of dendritic cells (DCs) upon diphtheria toxin-injection (*Cd11c*^*DTR/WT*^ mice) two days post *Lm*-Ova immunization (Fig. 4b, c). Primary OT-I T cell expansion in the blood was decreased by ~40–50%, confirming efficacy of both treatments (Supplementary Fig. 4a, b). CD11c^+^ cells were depleted in DT-treated *Cd11c*^*DTR/WT*^ mice, and when DT was given prior immunization, OT-I T cells failed to expand (Supplementary Fig. 4c^29^). Six weeks post immunization, OT-I memory cells (1000) from treated or control groups were sorted and co-transferred to recipient mice subsequently challenged with *Lm*-N4. OT-I memory cell expansion quantified 6.5 days later was similar whether epitope presentation, by either of the outlined methods, was disrupted or not. In these experimental settings, cognate Ag presentation by non-DCs, or as a result of insufficient mAb blockade, may however, still occur. Nevertheless, these data and the fact that both GP_33–41_- and p60_217–225_-derived epitopes were only presented for a short duration but exhibited distinct requirements on IL-2 signals to form functional memory, supported a model in which the strength of initial TCR signaling, but not the duration of cognate Ag presentation, most likely dictates whether naïve CD8^+^ T cells depend on IL-2 signals to form fully functional memory cells.

### Varying TCR and IL-2 signaling strength strongly modify memory CD8^+^ T cell chromatin accessibility but not transcriptomic profiles

**Fig. 5:**
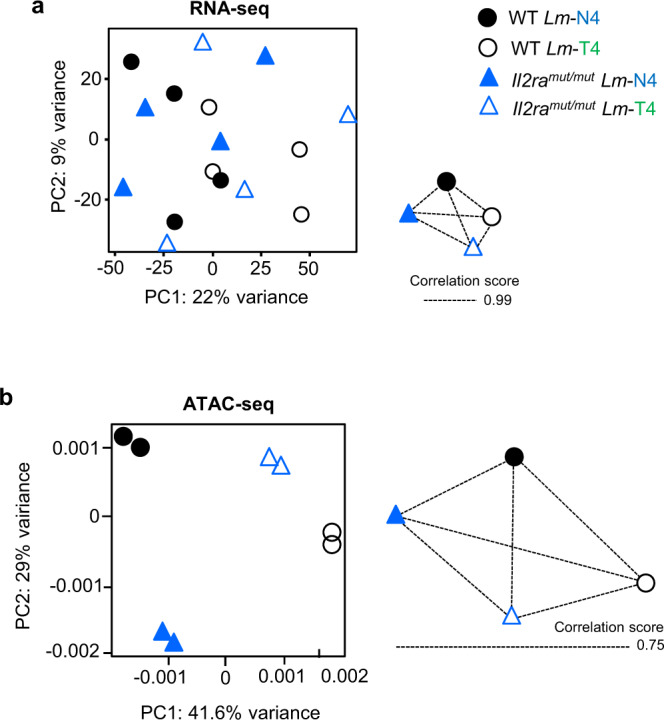
TCR and IL-2 signals alter chromatin remodeling but not gene expression in memory CD8^+^ T cells. *Il2ra*^*mut/mut*^ and WT OT-I cells were adoptively transferred to WT recipient mice, and infected the next day with *Lm*-Ova N4 or T4. 4 weeks later, OT-I memory cells were sorted by FACS and processed for RNA-seq and ATAC-seq analysis. Principal component analysis (PCA) of (**a**) RNA-seq and (**b**) ATAC-seq datasets. Correlation network of similarity between each condition in gene expression (RNA-seq) or chromatin accessibility (ATAC-seq) is also shown. Edge length corresponds to similarity (Pearson correlation). RNA-seq (*n* = 4 mice) and ATAC-seq (*n* = 3–4 mice) results were summarized from 2 independent replicates of experiments. Each symbol represents one mouse.

We sought to better understand the general mechanism by which different strengths of TCR and IL-2 signals shape memory CD8^+^ T cell programming, by comparing the transcriptional and chromatin accessibility profiles of *Il2ra*^*mut/mut*^ and WT OT-I memory CD8^+^ T cells induced upon weak (T4) or strong (N4) *Lm*-expressed epitope priming. Four weeks post-infection, resting OT-I memory cells from each of the four experimental groups were flow-sorted and whole genome analysis of both gene expression (by RNA-seq) and open chromatin regions (OCR) (by ATAC-seq) were conducted (Fig. 5, Supplementary Fig. 5 and Supplementary Data 2). Only 28 to 69 genes out of a total of 47, 643 genes (i.e., 0.05–0.15%) were differentially expressed at least 1.5 fold (adjusted *p*-value ≤0.05) when comparing gene expression between *Il2ra*^*mut/mut*^ and WT resting memory CD8^+^ T cells primed with weak or strong epitopes (Supplementary Fig. 5a, b and Supplementary Data 2). Very few differentially expressed genes were found across the various comparisons when only TCR (e.g., *cdc42ep2*) or IL-2 (e.g., *Eno1b*) varied but a larger number were found when both signals were concomitantly modulated (e.g., *Il27, Tnfsf4, Ncr1, Fcrl5, S1pr3, Il2*). Principal Component Analysis (PCA) based on the gene expression data showed a lack of independent clustering among these experimental conditions with PC1 and PC2 accounting only for 22 and 9% of variance, respectively (Fig. 5a). Pearson correlation comparisons among the different samples also indicated that gene expression was similar across all experimental groups (Score of 0.99). In contrast to gene expression profiles, PCA analysis of genome-wide OCRs highlighted significant differences between the groups, which were largely driven by the strength of TCR signaling (N4 versus T4), as shown in PC1 (Fig. 5b and Supplementary Fig. 6a). Lowering IL-2 signaling also contributed to differential chromatin remodeling that was most pronounced when OT-I cells received strong (N4) compared to weak (T4) TCR signals (in PC2). This was further confirmed using Pearson correlation scores that also revealed the highest similarities in OCRs between T4- (weak) primed OT-I memory cells, independent of IL-2 signaling strength, and the lowest similarities between N4- and T4-primed OT-I memory cells. In addition, Pearson correlation comparisons of OCR profiles among the various experimental conditions indicated that chromatin landscapes were substantially different (score of 0.75). Collectively, these results suggested that the strength of TCR and IL-2 signaling orchestrates memory CD8^+^ T cell functional programming through the modification of chromatin accessibility but not de novo gene expression.

### Modulating both TCR and IL-2 signaling strength together is required to induce broad chromatin remodeling

**Fig. 6:**
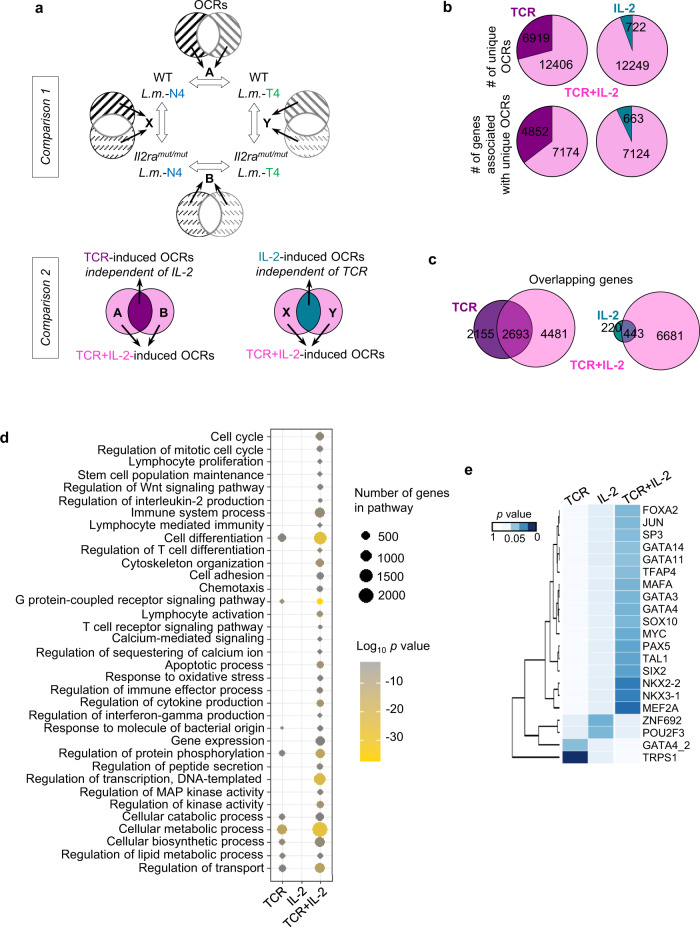
Extensive chromatin remodeling in memory CD8^+^ T cells is observed upon combined changes in both TCR and IL-2 signaling strength. **a** Schematic of the comparative analyses conducted between the open chromatin regions (OCRs) in OT-I memory cells (WT, *Il2ra*^*mut/mut*^) primed following *Lm*-N4 versus *Lm-*T4 infection. **b** Pie charts show the number of unique OCRs and related genes with unique peaks controlled by changes in TCR, IL-2 or TCR + IL-2 signaling from comparisons depicted in (A). **c** Venn diagrams show the number of unique or overlapping genes associated with peaks induced by modulating TCR, IL-2 or TCR + IL-2 signals. **d** Network analysis of biological-process GO term enrichment of the genes with unique peaks induced by changes in TCR, IL-2 or TCR + IL-2 signals. Over-represented GO terms were analyzed by Panther and visualized in a scatterplot. Node color is proportional to the FDR-adjusted *p*-value of the enrichment. Node size is proportional to the number of genes enriched in each GO term. Enrich in ClusterProfiler was used (over-representation analysis with FDR correction Benjamini and Hochberg. **e** Heat map shows the transcription factors enriched in unique OCRs induced by modulating TCR, IL-2 or TCR + IL-2 signals. ATAC-seq (*n* = 3–4 mice) results were summarized from 2 independent replicates of experiments. A linear model was used.

We assessed if the modulation of TCR, IL-2 or both signals induce distinct, overlapping or synergistic sets of OCRs genome-wide by conducting a side-by-side comparative analysis of the unique OCRs between *Il2ra*^*mut/mut*^ and WT OT-I memory cells primed with *Lm*-expressing strong (N4) versus weak (T4) APLs (comparison 1, Fig. 6a). Distribution of OCRs across the genome suggests a greater enrichment for TSS regions (<1 kb) in OT-I memory cells induced upon T4 versus N4 priming (Supplementary Fig. 6b). This first level comparison enabled to identify the OCRs that are unique when i) TCR signaling strength varies independent of IL-2 signals (A and B) and ii) when IL-2 signaling strength varies independent of TCR signals (X and Y). Next, through a second level comparison of the unique OCRs revealed by the first comparisons (A versus B and X versus Y), we defined which OCRs were induced by modulating either i) TCR but not IL-2 signaling strength, ii) IL-2 but not TCR signaling strength, or iii) both TCR and IL-2 signaling strength (comparison 2, Fig. 6a). This analysis showed 6,919 unique OCRs (~41%), i.e., 4,852 potentially associated genes (defined as the nearest, see Methods), common between *Il2ra*^*mut/mut*^ and WT memory cells that received weak versus strong TCR priming signals; therefore, these OCRs were induced upon modulating the strength of TCR signaling whether or not intact IL-2 signaling was present (Fig. 6b). Only 722 OCRs (~6%), i.e., 663 potentially associated genes, that were common to the memory cells that received either strong or weak TCR signals, were uniquely induced by altering IL-2 signals, establishing that modulating the strength of each signal orchestrates the accessibility of different regions of the chromatin. Most interestingly, when both TCR and IL-2 signaling strengths were changed together, this led to the opening of a completely new set of 12,406 or 12,249 unique OCRs, i.e., respectively 7174 or 7124 potentially associated genes, depending on the comparison run. As expected, the unique OCR-associated genes in both comparisons almost fully overlapped (98%, Supplementary Fig. 6c). This result suggested that intact IL-2 signaling alone could not rescue weak TCR priming signals to remodel chromatin, but rather acted synergistically with TCR signals during priming to program chromatin accessibility in memory cells.

Of the genes associated with unique OCRs induced by changes in TCR or IL-2 signaling strength, a substantial proportion of them (~55 to 67%) overlapped with the genes associated with unique OCRs induced by modulating both TCR and IL-2 signals (Fig. 6c and Supplementary Data 3). However, more than 60% of the genes associated with OCRs induced by modulating TCR and IL-2 signals were unique and did not overlap with the genes with unique OCRs induced by either varying TCR or IL-2 signaling strength only. Biological process gene ontology (BP-GO) analysis of the non-overlapping genes from Fig. 6c showed significantly greater diversity of biological processes regulated by modulating both TCR and IL-2 signals compared to TCR only while changes in IL-2 signaling did not yield any significant GO pathways (Fig. 6d, Supplementary Data 4). The pathways associated with the genes exhibiting unique OCRs made accessible by TCR and IL-2 signal changes were related to cell cycle, proliferation and IL-2, stem cells, T cell activation, differentiation and immunity, G-protein-coupled receptor and calcium signal transduction, transcription, chemotaxis and adhesion, cytokine response, apoptosis, metabolism and catabolism. In contrast, the pathways associated with OCRs induced by changes in TCR signaling strength were much more restricted and included immune defense, G-protein coupled receptor signaling and metabolic/catabolic biosynthetic processes.

We next hypothesized that specific groups of transcription factors (TFs) may bind more selectively to the distinct OCRs and contribute to drive specific functional features of the memory cells. Using a motif analysis approach, we then identified candidate TF-binding motifs across OCRs induced upon modulating TCR, IL-2 and TCR + IL-2 signals respectively, and performed enrichment analysis for known motifs based on the HOMER/JASPAR database. We next ran a PCA analysis to examine which TFs may account for segregating the three sets of OCRs (Fig. 6e). We discovered 21 TFs, among which 17 that were enriched in the OCRs induced by changes in TCR + IL-2 signals, while only two were enriched in OCRs induced by varying TCR or IL-2 signals only. Altogether, these results supported the idea that modulating the strength of both TCR and IL-2 signals together programmed memory CD8^+^ T cells with a greater set of OCRs and putative TFs binding sites, consistent with an improved functional fitness.

### Genes involved in memory CD8^+^ T cell stemness, cell cycle and calcium fluxes have promoter regions accessible to key transcription factors

**Fig. 7:**
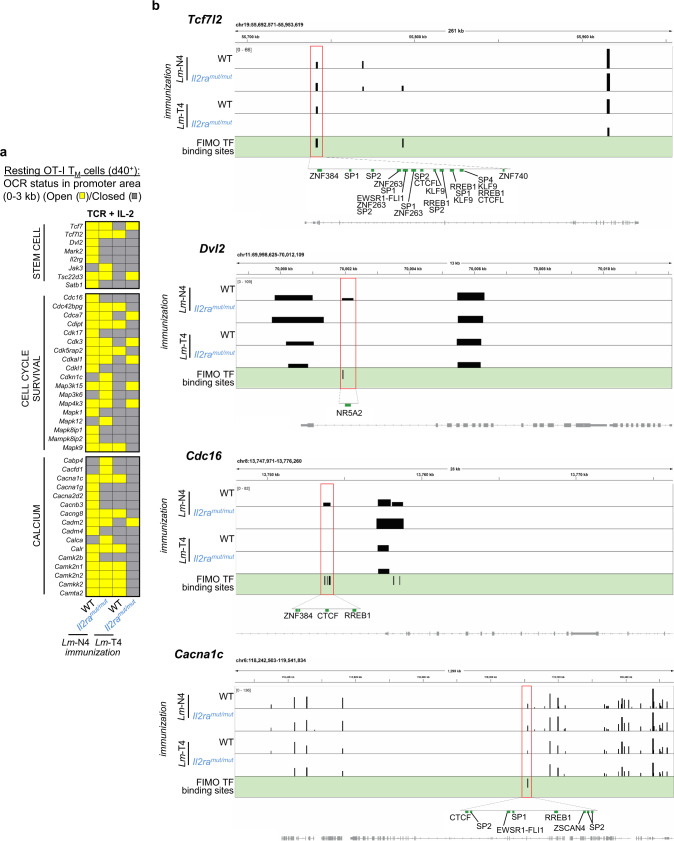
Chromatin accessibility and predicted TF binding in promoter areas of stem cell, cell cycle and calcium flux-related genes of memory CD8^+^ T cells primed under distinct strengths of TCR and IL-2 signals. **a** Heat map shows unique OCRs (yellow) in genes encoding stem cell-related proteins, cell cycle/proliferation and calcium fluxes that were indicated in Fig. 6b when TCR and IL-2 signals were modulated together, in the promoter area and close to the TSS (±~3 kb). Chromatin region status (open, yellow/closed, gray) in WT and *Il2ra*^*mut/mut*^ OT-I memory cells primed with *Lm*-N4 or *Lm*-T4 are reported. **b** Selected examples of genes (*Tcfl2, Dvl2, Cdc16, Cacnac1*) with differential OCRs (red box) in these OT-I memory CD8^+^ T cells. Higher magnifications of the OCRs show FIMO-predicted binding sites for indicated TFs.

We next investigated if the reported changes in global chromatin accessibility targeted functionally relevant genes in memory CD8^+^ T cells. We focused on genes that exhibited differentially accessible OCRs in the promoter area close to the TSS (±3 kb) in WT and *Il2ra*^*mut/mut*^ OT-I memory cells primed with weak or strong TCR signals. We postulated that such OCRs were more likely to reflect direct phenotypic and functional differences in memory CD8^+^ T cells. We noted differentially accessible OCRs in clusters of genes related to stemness, cell cycle and calcium fluxes, which all represent important hallmarks of T cell functional fitness and activation (Fig. 7a and Supplementary Data 5). The greater chromatin accessibility in the promoters of most of these genes in OT-I memory cells primed with strong TCR and IL-2 signals (i.e., WT, *Lm*-N4) was remarkable while these regions were largely closed in those that received weak TCR and IL-2 signals (i.e., *Il2ra*^*mut/mut*^*, Lm*-T4). In T cells that received either weak TCR or IL-2 signals, chromatin accessibility was more nuanced (even if TCR signals had a stronger impact), suggesting that other mechanisms also contributed to accessibility.

We next selected genes involved in the distinct biological processes highlighted above that included *Tcf7, Tcf7l2, Dvl2, Mark2*, *Il2rg, Cdc16, Cdk17, Mapk9, Cacna1c, Cacng8. Tcf7, Tcf7l2, Dvl2* and *Mark2* encoded proteins are involved in the activation the Wnt/β-Catenin pathway while IL-2Rγ transduces common γ-chain cytokine signals (i.e., IL-2, IL-7 and IL-15). *Cdc16*, *cdk17* and *Mapk9* encoded proteins control cell cycle progression whereas that from *Cacna1c* and *Cacng8* genes cooperate to form and regulate L-type high voltage calcium channels implicated in intracellular calcium accumulation. We then evaluated likelihood of binding for known TFs in the differentially accessible promoter-region of these genes using Find Individual Motif Occurrence (FIMO; Fig. 7b, Supplementary Fig. 7b and Supplementary Data 5). While we did not find any TFs binding sites in *Tcf7, Mark2* or *Cdk7* promoter OCRs, we characterized binding sites for multiple TFs in *Tcf7l2, Dvl2, Il2rg, Cdc16, Mapk9, Cacna1c* and *Cacng8* promoter OCRs.

Notably, OCRs associated with the promoter regions of selected genes, such as *Tcfl2*, *Cdc16*, *Cacna1c*, *Cacng8* and *Il2rg*, contained a greater number of putative TF binding sites and higher diversity in families of TFs (i.e., SP, ZNF, KLF, CTCF, RREB) that putatively bind at these sites, suggesting these OCRs and TFs could have important functions in regulating expression of these genes (Fig. 7b and Supplementary Data 5). In contrast, other OCRs in genes like *Dvl2, Cdkl1* and *Mapk9* only exhibited limited TF binding sites, suggesting that these areas may either be less important or more specific in regulating expression of these genes. Thus, this analysis showed that many of the differentially accessible OCRs in genes potentially involved in stemness, cell cycle and calcium fluxes may be subjected to further regulation by families of TFs, consistent with a greater functional fitness of the memory cells primed by strong TCR and IL-2 signals.

### The greater chromatin accessibility in memory CD8^+^ T cells primed with strong TCR and IL-2 signals is consistent with their higher subset diversity and phenotypes

**Fig. 8:**
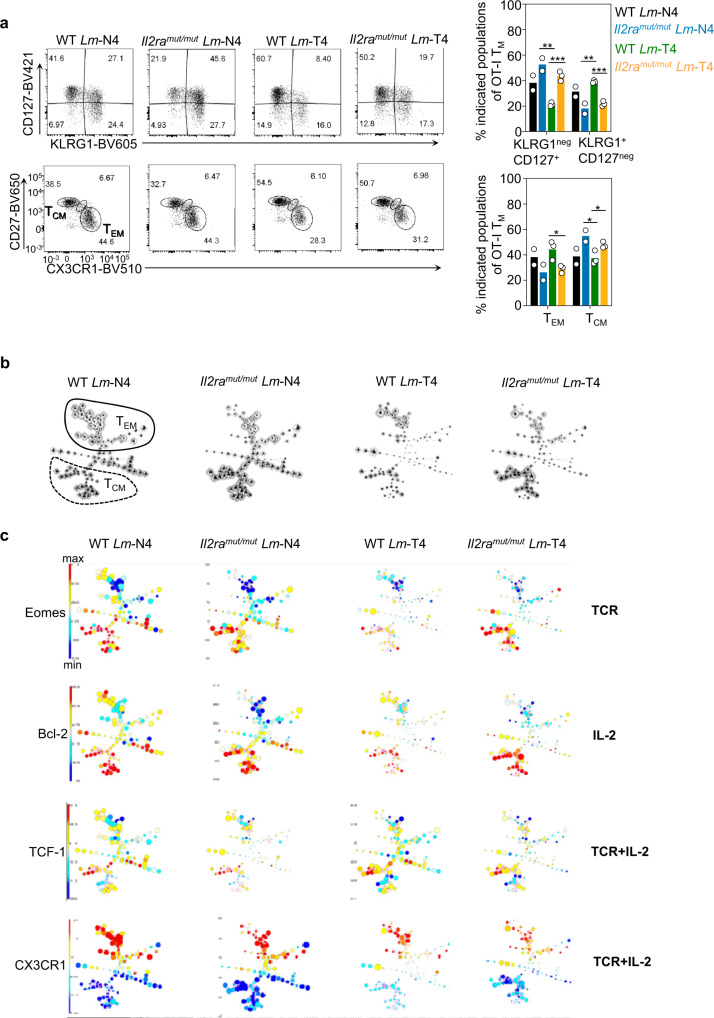
Robust TCR and IL-2 signals induces the most memory CD8^+^ T cell subset diversity and phenotypes. **a** FACS analysis of *Il2ra*^*mut/mut*^ and WT OT-I memory cells primed after infection either *Lm*-N4 or *Lm*-T4 ~5 weeks later, after staining with a panel of 26 memory cell-relevant markers. Bar graphs show classically defined effector (T_EM_), central memory (T_CM_) subsets or based on cell-surface expression of KLRG1, CD127, CD27 and CX3CR1 (*n* = 2 mice for *Lm*-N4 and 3 mice for *Lm*-T4 in 1 representative of 2 independent replicate experiments). **b** FlowSOM analysis of each experimental group containing a pool of concatenated OT-I memory cells from 3 mice. Where classically defined T_EM_ and T_CM_ subsets are, is manually circled. **c** The expression level of indicated markers (Eomes, Bcl-2, TCF-1, CX3CR1) within each node, regulated by each or combined priming signal(s), is represented in a color scale. Data are pooled from 3–4 mice across 2 replicate experiments.

If the epigenetic differences observed in both the breadth of OCR-related GO pathways and increased accessibility of stem-cell related gene promoters in memory CD8^+^ T cells induced by strong TCR and IL-2 signals reflects functional relevance, memory CD8^+^ T cells primed with strong signals should give rise to a greater diversity of subsets and phenotypes than those that received weak TCR and/or IL-2 signals. Using high-dimensional flow cytometry with a 26-color panel that included cell-surface markers, intracellular functional markers and TFs relevant to CD8^+^ T cells (Supplementary Table 1), we conducted an in-depth characterization of resting OT-I memory cells primed with weak or strong TCR and IL-2 signals (Fig. 8 and Supplementary Fig. 8). Focusing first on known memory CD8^+^ T cells subsets such as terminally differentiated KLRG1^+^ or CX3CR1^+^CD27^-^ effector memory (T_EM_) cells and CD127^+^KLRG1^-^ or CX3CR1^-^CD27^+^ central memory (T_CM_) cells^14,30^ indicated a higher proportion of T_EM_-like cells when primed with intact versus weak IL-2 signals (Fig. 8a), a finding consistent with IL-2 signals promoting differentiation of robust effector cells^16,31^. Interestingly, however, increasing TCR signals neither altered the proportion of T_EM_ or T_CM_ cells, underscoring the need to achieve deeper resolution of memory subsets to find more granular differences.

Thus, we next processed the analysis of our high-dimensional flow cytometry data using flow self-organizing map (FlowSOM) that enables unsupervised clustering and dimensionality reduction, and the visualization of discrete memory cell subsets and their relative proportions (Fig. 8b). This analysis showed a remarkable heterogeneity of potential subsets in T_EM_ and T_CM_ cells. Memory cell subset diversity and relative proportions were closely similar in *Il2ra*^*mut/mut*^ or WT OT-I cells (only IL-2 signals differ) primed with either *Lm*-expressing N4 or T4 epitopes. In contrast, memory cells (whether *Il2ra*^*mut/mut*^ or WT) primed with *Lm*-expressing N4 or T4 epitopes (only TCR signaling strength differed) exhibited greater subset diversity and included many differentially represented subsets. Importantly, OT-I memory cell subset diversity and relative proportions in *Il2ra*^*mut/mut*^ OT-I cells primed with T4 compared to WT counterpart primed with N4 or vice-versa (when both TCR and IL-2 signals differ), had the most differentially represented subsets. Deeper FlowSOM analysis on individual cell-surface and intracellular marker expression indicated the relative effect of each or combined signal(s) on the FlowSOM outlined subsets of memory cells (Fig. 8c and Supplementary Figs. 8, 9). In particular, markers associated with a T_CM_ cell phenotype (TCF-1, Eomes, Bcl-2, Sca-1, CD122, CD127, CD27) exhibited significantly higher expression in the subsets included in the broad category of T_CM_ cells. The combined modulation of both TCR and IL-2 signals at priming had the most impact on the majority of markers (e.g., TCF-1, CX3CR1 and most others), also consistent with a greater chromatin accessibility in the promoter of stem-cell related genes (e.g., *Tcf7, Tcf7l2, Il2rg*) in memory cells primed under strong TCR and intact IL-2 signals (Fig. 7). Of note, some markers were more selectively affected by TCR (i.e., Eomes, IRF4) or IL-2 (i.e., Bcl-2) signal changes. Thus, collectively, these results are in agreement with our chromatin landscape analyses indicating that modulating TCR, IL-2 or TCR + IL-2 signaling strength sets distinct non-overlapping OCRs that are likely to be linked to the phenotypic output of potential memory cell subsets.

### Differentially accessible OCRs in genes involved in the control of stemness, cell cycle and calcium fluxes in memory CD8^+^ T cells correlate with distinct functional responses

As an attempt to further link the reported changes in chromatin accessibility to functional differences in the memory CD8^+^ T cells, we focused on the specific clusters of genes with differential accessibility in the promoter area shown in Fig. 7. We hypothesized that such OCRs were likely to be essential in the rapid and differential modulation of memory CD8^+^ T cell activation in vivo. Using FACS analysis, we tested whether WT or *Il2ra*^*mut/mut*^ OT-I memory cells induced upon immunization with *Lm*-expressing weak (T4) or strong (N4) Ova APLs, exhibited different abilities to undergo rapid intracellular calcium signaling, enter cell cycle (16 h), and expand (day 4, 6, 9 and 12) after *Lm*-N4 challenge infection (Fig. 8a).

Intracellular calcium accumulation early on after the secondary challenge infection (16 h) was significantly higher in N4-primed OT-I memory cells compared to T4-primed counterparts, whether on the WT or *Il2ra*^*mut/mut*^ background (Fig. 8b). This indicated that higher calcium signaling in memory CD8^+^ T cells largely depended on strong TCR signals at the time of priming, despite many relevant OCRs being differentially accessible when both TCR and IL-2 signals varied (Fig. 7). However, we also noted that *Cabp1* (Calcium binding protein 1) and *Camk1* (Calcium/calmodulin-dependent protein kinase) accessible OCRs were only modulated by TCR signaling strength, suggesting the products encoded by these genes could potentially act as key regulators of intracellular calcium fluxes in early reactivated memory CD8^+^ T cells (Supplementary Fig. 10a).

**Fig. 9:**
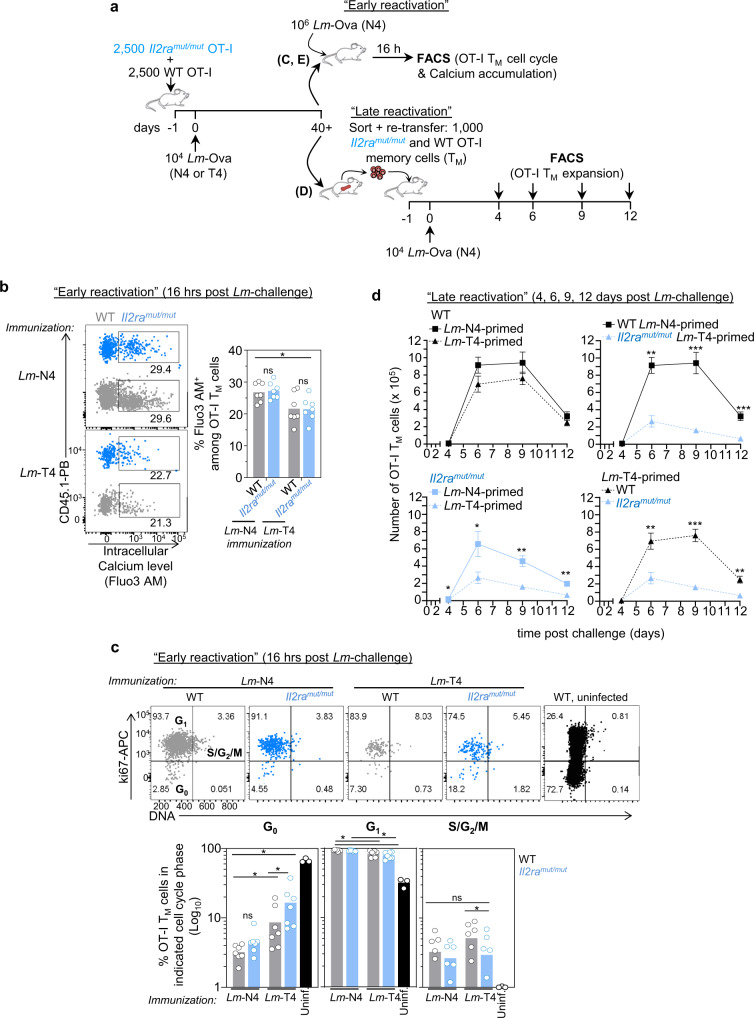
Memory CD8^+^ T cells induced with robust TCR and IL-2 signals initiate faster calcium signaling, cell cycling and expand more competitively than those primed with weaker signals. (**a**) Schematic of experimental designs used in (**b**–**d**). Briefly, 2500 *Il2ra*^*mut/mut*^ or WT OT-I Td^+^ cells were adoptively transferred to WT naïve hosts, and infected the next day with *Lm*-Ova N4 or *Lm*-Ova T4, and housed for ~40 days before the next steps. In (**b**, **c**), immunized mice were challenged with 10^6^ *Lm*-Ova N4 and 16 h later (“early activation”), spleen cells were either (**b**) loaded with Fluo3 AM or not (*p* = 0.0129) (**c**) and stained for cell-surface CD3, CD8, congenic markers. In (**c**) cells were stained for intracellular expression of Ki67 and DNA. Representative dot plots comparing *Lm*-OvaN4 versus *Lm*-Ova T4-primed reactivated OT-I memory cells for indicated marker expression. Graphs summarize the average of 7 mice for each of the 4 experimental conditions across 2 independent replicate experiments. In (**d**), 1000 *Il2ra*^*mut/mut*^ and WT OT-I memory cells from each priming condition, were FACS-sorted and transferred at a 1:1 ratio to new hosts subsequently infected with *Lm*-OvaN4 the day after. At indicated days, spleen cells were stained to quantify *Il2ra*^*mut/mut*^ and WT OT-I memory cell expansion and numbers. Data are the pool of 2 independent replicate experiments (n = 8–14 mice per group per timepoint; each symbol represents one mouse) and are presented as mean cell number with error bars indicating SEM. In all panels, *p*-values are indicated when relevant with *p < 0.05; **p < 0.01; ***p < 0.001; ns, not significant, using two-tailed unpaired Student’s *t* test.

At early stages after activation, we also found that a significantly greater proportion of T4-induced *Il2ra*^*mut/mut*^ and WT OT-I memory cells (factor of ~6 and 3, respectively) remained in the G_0_ phase while N4-counterparts had already transitioned to the G_1_ phase (Fig. 9c and Supplementary Fig. 10b). As expected, a much higher proportion of naïve T cells were in G_0_ compared to early activated N4-memory or effector (day 5) cells (up to ~20 times more). In addition to their faster initiation of cell cycle, N4-induced WT OT-I memory cells underwent more robust and competitive later expansion than both WT and *Il2ra*^*mut/mut*^ OT-I memory cells primed with the weak T4 agonist (Fig. 9d and Supplementary Fig. 10c). Memory cell numbers and frequencies quantified 6, 9, and 12 days post-*Lm*-N4 challenge infection were significantly lower (factor of 2–4) when OT-I cells had been primed with weak TCR and IL-2 signals. These results showed that rapid memory cell entry into cell cycle and competitive proliferation/expansion largely depended on the combination of robust TCR and IL-2 signals during priming. These findings were also consistent with the differentially accessible OCRs noted in the promoters of cell-cycle checkpoint (i.e., *cdc16, cdkl1, cdkn1c*), stem-cell (*Tcf7l2, Dvl2*) and IL-2 (*Il2rg, Jak3, Tsc22d3*) related genes (Fig. 7).

In summary, differential chromatin accessibility at promoter area of genes related to intracellular calcium processes, cell cycle, proliferation and stemness, correlated with distinct functional responses of reactivated memory cells induced by strong or weak TCR and/or IL-2 priming signals. Consistent with our epigenetic analysis, these results support a model of greater fitness of memory cells primed with stronger TCR and IL-2 signals while those that received weak TCR and/or IL-2 signals were sluggish to rapidly activate, enter cell cycle and expand (Supplementary Fig. 11).

## Discussion

Here, we show that the strength of TCR signaling is a key driver of memory CD8^+^ T cell functional characteristics and fitness. We establish that the amount of TCR signals integrated by naïve CD8^+^ T cells during priming dictates whether IL-2 signals are required for them to form fully functional memory CD8^+^ T cells, i.e., (i) that are able to undergo rapid reactivation (calcium signaling, cell cycling) and competitively expand during a rechallenge infection, and (ii) that exhibit higher stemness characteristics. We also show that the concomitant modulation of TCR and IL-2 signals has a profound impact on memory CD8^+^ T cell epigenetic landscape, as measured by a significantly increased chromatin accessibility. In contrast, the extent of chromatin remodeling and opening in memory cells primed under lower TCR or IL-2 signaling strengths was much more reduced.

Current dogma states that increasing the strength of TCR signals in CD8^+^ T cells (i) promotes an effector versus a memory CD8^+^ T cell fate^10,11^, (ii) augments the size of primary CD8^+^ T cell expansion and of the memory cell pool^7,19^, and iii) does not alter the functional features of memory cells^19^. While our results confirm prior findings (i) and (ii), they nevertheless challenge the concept (iii) that TCR signaling strength does not modulate the functional attributes and programming of memory CD8^+^ T cells. Although CD8^+^ T cells primed by weak TCR signals give rise to significantly lower numbers of memory cells most likely as a result of curtailed expansion^19^, our data also show (i) that lowering TCR signaling strength alters their programming through distinct chromatin remodeling and (ii) the requirement for intact IL-2 signaling in order to form fully functional memory cells.

The importance of IL-2 signals in enabling the formation of functional memory cells has been previously documented^32^, but it has also remained controversial^33^. We confirm here the results of the prior studies by showing that T cells primed with the natural OvaN4 epitope do not require IL-2 signals during priming^33^ whereas those primed with the LCMV-derived GP_33-41_ epitope do^32^. By establishing that TCR signaling strength, whether as a result of weak epitope/MHC stability or epitope/MHC/TCR interactions, is an important variable controlling CD8^+^ T cell-dependency on IL-2 signals during priming, we further provide a rational explanation for the earlier discrepancy. Importantly, these results are also consistent with the idea that a quantum threshold of TCR signals needs to be reached for naïve CD8^+^ T cells to bypass the need for intact IL-2 signals and form a pool of functional memory cells. Below such threshold, even though CD8^+^ T cells still expand and form memory, they fail to become fully functional, e.g., equipped to undergo optimal reactivation, initiate cell cycle and competitively expand. While this may represent a safeguard mechanism to prevent memory T cell clones to be reactivated against self- or tumor-derived antigens of low affinity^20^, this result also suggested that the induction of effective memory CD8^+^ T cell responses against weak T cell epitopes, requires robust IL-2 signaling. These findings are particularly relevant in the context of recent studies using an IL-2 superkine partial agonist that preferentially triggers the ERK pathway in spite of STAT5 downstream of the IL-2 receptor, skewing CD8^+^ T cell differentiation towards a stem cell fate^34,35^. Our results suggest that the acquisition of such superior functional features through IL-2 signaling is very likely to be also regulated by the amount of TCR signals received by naïve CD8^+^ T cells during priming. These findings have important implications for improving the rationale design of vaccines and T cell therapies targeting low affinity, tumor-derived antigens in particular.

Approximately one-third of the naturally selected microbial pathogen-derived epitopes we have studied appear to induce CD8^+^ T cells that are dependent on intact IL-2 signaling to differentiate into memory cells that competitively re-expand during antigen re-encounter. This suggests that this mechanism is likely to extend beyond the Ova model system, increasing its potential significance in epitope selection and vaccine design. While our data are consistent with such possibility, it has also been established that Foxp3^+^ regulatory T (Treg) cells favor the priming of high versus low affinity polyclonal CD8^+^ T cells^36^. It is therefore conceivable that IL-2 produced in close proximity to low affinity naïve CD8^+^ T cells that cannot effectively bind IL-2 (i.e., *Il2ra*^*mut/mut*^ or *Il2ra*^*−/−*^), is consumed by Treg cells, further promoting primary expansion of higher affinity clones with a distinct TCR repertoire that would require IL-2 signals during priming for competitive re-expansion. Whether Treg cells are involved or not in expanding a distinct set of T cells, our data together with current literature, remain consistent with the concept that IL-2 has an essential function in the functional imprinting of memory CD8^+^ T cells.

The findings presented in this work are also consistent with several studies showing that the strength of TCR signaling in naive CD4^+^ T cells is an essential determinant of T helper cell effector fates, for which strong TCR signaling skews their differentiation towards Th1^37^, follicular helper (T_FH_)^38^ or germinal center (GC-T_FH_) CD4^+^ T cells^39^. TCR/peptide MHC dwell times rather than equilibrium binding was proposed to predict T helper effector cell fates, with long dwell times promoting T_FH_ or GC-T_FH_ rather than Th1 effector cell fates^39^. Recent reports further involved IL-2 and its signaling as an important mechanism orchestrating such CD4^+^ T cell fates^40,41^. In one study, strong TCR-stimulation with long dwell times skewed naïve CD4^+^ T cell differentiation into effector T_FH_ cells (Bcl-6^+^) and endowed them with IL-2-producing capacity, consistent with a model in which IL-2-secreting cells are precursors of T_FH_ cells^41^. Non-IL-2-secreting CD4^+^ T cells, that received weaker TCR signals and IL-2 from IL-2-secreting CD4^+^ T cells, rather differentiated into non-T_FH_ effector cells (Blimp-1^+^). In contrast, in the other study, strong TCR stimulation, which induces high levels of cell-surface CD25, skewed CD4^+^ T cell-differentiation into Th1 effector cells, while weaker TCR stimulation, and therefore low levels of cell-surface CD25, was associated with a Th1 and T_FH_ memory cell fate^40^. In line with our results and the literature on CD8^+^ T cells^10,19^, this latter study showed that decreasing TCR signals induced memory CD4^+^ T cells that competitively expanded and produced effector functions. Whether weakly stimulated CD4^+^ T cells lacking IL-2 or even other cytokine signaling pathways would fail to form functional memory cells is not known and would need further investigations.

Another important message of this work is that the combination of strong TCR and IL-2 signals during CD8^+^ T cell priming together imposes important modifications in the chromatin accessibility of memory CD8^+^ T cells, therefore alters naïve CD8^+^ T cell long-term programming. We report that combining these signals enables the opening of a distinct genome-wide epigenetic landscape that only moderately overlaps with the landscapes modulated by changes in either TCR or IL-2 signals and appears much broader. The breadth of biological processes targeted and the greater number of TFs (17 compared to 2) that can potentially bind to accessible chromatin regions uniquely present in memory cells primed by strong TCR and IL-2 signals, support the interpretation that combining these signals induces synergistic mechanisms of chromatin remodeling that are not turned on by each individual signal. It is likely that establishment of these distinct epigenetic landscapes occurs at early effector stages after priming and involve DNA/histone-modifying methyltransferases that control chromatin accessibility in memory precursor or effector cells^23,24^. Another non-exclusive possibility based on our data is that combinations of TCR- and IL-2-dependent transcriptional regulators could act together as genome organizers. Prior reports have shown that IRF4 and BATF TFs for instance bind DNA cooperatively to initiate chromatin remodeling and STAT-dependent transcriptional programs in Th17 CD4^+^ T cells^42^ and early effector CD8^+^ T cells^43^. Our transcriptomic analysis at early stages post-priming highlighted that genes encoding both IRF4 and BATF3 TFs were upregulated in OT-I cells primed with robust TCR signals, which could contribute to establishing the distinct epigenetic landscape that we observed.

The analysis of the differentially accessible OCRs in the promoter areas of resting memory cells primed with robust TCR and IL-2 signals showed clusters of genes involved in stem-cell features, cell cycling and calcium fluxes. Our FIMO analysis further indicated that many TFs belonging to distinct families, can potentially bind to these areas. This finding suggested that these differentially accessible promoter regions may have important functions in the expression of these various genes and the functional features of the memory cells. Both the higher chromatin accessibility in genes promoters associated with the Wnt/β-catenin pathway as well as the genome-wide enrichment in predicted binding sites of TFs such as GATA-3, TAL-1, NKX3-1, SIX2 which act as lineage commitment TFs and are involved in stem cell maintenance^44,45^, are consistent with the superior functional features and subset diversity of the memory cells primed under robust TCR and IL-2 signals.

Our comparative high-dimensional FACS analysis of resting memory CD8^+^ T cells is also consistent with differences in stem-cell related gene promoter accessibility. While subdividing the memory cells based on T_EM_ and T_CM_ subsets failed to find differences other than those likely accounted for by intact IL-2 signaling (which favors KLRG1^+^ effector cell differentiation^16,31^), FlowSOM analysis and visualization achieved deeper resolution of potential subsets of memory cells. This enabled a more precise assessment of how much the strength of each signal modulated the size of individual subsets and expression levels of a given memory cell marker. This highlighted that when both TCR and IL-2 signals were co-modulated, the diversity of subsets and their expression of markers driving stemness (TCF-1, Eomes, Sca-1, CD127, CD62L, CD44) were clearly increased. Importantly, the better chromatin accessibility in promoters of genes controlling cell cycle initiation, calcium fluxes and proliferation in resting memory CD8^+^ T cells primed with robust TCR and IL-2 signals correlated with the functional outcomes after their reactivation. These memory cells accumulated intracellular calcium, initiated cell cycle and expanded significantly more than those that were primed with low TCR and IL-2 signals. Since multiple TFs were predicted to bind in these OCRs, it is conceivable that several levels of regulation take place. Interestingly, providing IL-2 signals to the IL-2 signaling defective memory CD8^+^ T cells rescues their ability to competitively expand, indicating that optimal clonal expansion can be achieved through STAT5 signaling. However, given the genome-wide differences in chromatin accessibility between the memory cells primed with distinct TCR and IL-2 signals, it seems unlikely that giving IL-2 signals during reactivation of the memory cells primed with weak TCR and IL-2 signals is sufficient to rescue an epigenetic landscape associated with “high fitness”.

## Methods

### Ethics statement

This study was carried out in strict accordance with the recommendations by the animal use committee at the Albert Einstein College of Medicine. All efforts were made to minimize suffering and provide humane treatment to the animals included in the study.

### Mice

All mice were bred in our SPF animal facility at the Albert Einstein College of Medicine. Housing conditions include a 12 h light/dark cycle, room temperature set to 70° F, and humidity maintained within a 30–70% range. For all experiments, we used 6–10 week old male and female mice that were age- and sex-matched. Females and males were used equally to prevent any gender biases in results. The study has ethical approval by the Albert Einstein College of Medicine, under protocol numbers 20180506 and 00001375. We used wild-type (WT) C57BL/6J (B6) 6–8 weeks old male or female mice, congenic CD45.1^+/+^ (JAX#002014), OT-I^+^ (JAX#003831), P14 (JAX#004694, backcrossed to B6 > 6 times), CD11c-DTR^+/−^ (JAX#004509) and Rosa26-Actin-tomato-stop^loxP/loxP^-GFP (TdT)(JAX#007576) all purchased from the Jackson labs. *Il15*^*−/−*^ (stock#4269) mice were purchased from Taconic farms. We also bred gBT-I^46^ (gift Dr. Carbone), L9.6^+^ Kd^+^^47^, *Il2ra*^*mut/mut*^^28^^,^ and *Ifnar*^*−/−*^ mice (gift Dr. Kohlmeier, Emory Vaccine Center). All mice are on the B6 genetic background unless otherwise specified.

### Microbial pathogens and mouse infections for primary and memory response analyses

#### Listeria monocytogenes (Lm) bacteria and infections

*Lm* on the 10403s genetic background^48^ was used to express different antigens: Ovalbumin (*Lm*-Ova_257–264_ (N4)) and its APLs (T4, A8, Q4), Herpes Simplex Virus 2 (*HSV-2*) glycoprotein B (*Lm*-gB_498–505_), lymphocytic choriomeningitis virus (*LCMV*) glycoprotein (*Lm*-GP_33–41_, *Lm*-GP_276–286_). *Lm*-expressing N4, T4, Q4, GP_33–41_, gB_498–505_, GP_276–286_ were obtained from D. Zehn^19^. We generated *Lm*-A8 according to published methods^49^. Briefly, a DNA fragment encoding for a chimeric protein Ova_209–309_-gB_447–550_ containing the Ova A_264_ (A8) mutation and the gB_498–505_ epitope was synthetized (Genewiz) and cloned into the pHSLV *Lm* transfer vector for *Lm* selection and expression under the LLO/*Hly* promoter. All *Lm* were prepared after passaging into WT B6 mice, by growing to log phase (OD_600_~0.3–0.4) and kept as frozen aliquots for single use in −80 °C. For infections, bacteria were grown to a logarithmic phase (OD600~0.05–0.15) in broth heart infusion medium, diluted in PBS to infecting concentration (10^4^/mouse) and injected i.v. Secondary challenge infections were performed 4–6 weeks later with 10^6^ *Lm*-Ova (N4) unless otherwise specified in the figure legends.

#### Herpes Simplex virus 2 (HSV-2) strains and infections

Virus stocks, both WT HSV-2 (strain 186) and TK^-^ HSV-2 (186ΔKpn) were prepared in Vero cells using standard procedures and virus stocks were kept at −80 °C. *Prior to infections, female* mice were treated with 2 mg medroxyprogesterone acetate subcutaneously (s.c.) and 5 days later were inoculated intravaginally with 2 × 10^5^ plaque forming units (PFU) of TK^-^ HSV-2. For primary infection, spleens were harvested 7.5 days later for FACS analysis. The WT HSV-2 virus was used for i.v. rechallenge infections 60 days after immunization and spleens were harvested 6.5 days later for FACS analysis.

#### Vesicular Stomatitis Virus (VSV) strains and infections

*VSV* encoding *Lm* listeriolysin O (LLO_91–99_) and p60_217–225_^50^ or Ova (gift Kamal Khanna, NYU) kept at −80 °C were thawed and diluted in cold PBS right before infections. 2 × 10^5^ PFU/mouse were injected i.v. into mice. For primary infection, spleens were harvested 7.5 days later for FACS analysis. For rechallenge infections, mice were infected i.v. with *VSV*-LLO_91–99_-p60_217–225_ at least 40 days after immunization and spleens were harvested 6.5 days later for FACS analysis.

### RMA-S peptide:MHC stabilization assay

10^5^ Transporter Associated With Antigen Processing deficient (TAP^−/−^) RMA-S cells were added per well to a 96-well flat-bottom plate and incubated at 30 °C overnight. The next day, various concentration of each tested peptide were added and incubated at 37 °C for 4 h before staining for cell-surface expression of H2-K^b^ or H2-D^b^ expression by FACS. For the LCMV-derived GP_33–41_ peptide, we used the Methionine variant (M41) as the natural Cysteine residue in position 41 quickly oxidizes during peptide storage leading to peptide dimerization^51^.

### In vitro T cell assays

#### Antigen presenting cell (APC) preparation

Single cell suspension was prepared from WT B6 spleens, lysed with ACK lysis buffer, and incubated with 1 mL of anti-CFTR in 1X PBS for 1 min at 37 °C before quenching with 10 mL of complete RPMI media. The APCs were then mixed with indicated concentrations of Ova agonist peptides (N4, T4, A8) and E1 antagonist control for 2 h at 37 °C. APCs were then rinsed and resuspended in complete RPMI media.

#### T cell preparation and co-culture setup

Single cell suspension was prepared from WT or *Il2ra*^*mut/mut*^ OT-I spleens, lysed with ACK lysis buffer, and stained with CTV (Invitrogen) according to the manufacturer’s protocol. The APCs loaded with Ova peptides were co-cultured with CTV-labeled T cells at a 3:1 ratio in a tissue treated 96-well v-bottom plates. Plates were loaded into a 37 °C incubator attached to custom Tecan Freedom EVO 75 robotic platform programmed to conduct automated robotic time series that collected supernatant for cytokine analysis and cell pellets for surface/intracellular marker analysis. For cytokine quantifications, co-culture supernatants were thawed at room temperature for 1 h and cytokines stained with mouse Th1/Th2/Th17 cytokine kit (BD) according to the manufacturer’s protocol. For proliferation and activation, cell pellets were incubated with fluorescently tagged antibodies (see Supplementary Data 5) at room temperature for 30 min. All samples were next acquired on a BD Fortessa FACS HTS.

#### Preparation of cell suspensions and staining for flow cytometry analysis

Spleens or lymph nodes (inguinal and cervical) were dissociated on a nylon mesh. Cell suspensions were treated with red blood cells (RBC) lysis buffer (0.83% NH_4_Cl vol/vol). Blood was harvested into heparin tubes and RBC lysed. Cell suspensions were incubated with 2.4G2 Fc Block and stained with fluorescently tagged antibodies (See Supplementary Table 1) in FACS buffer (PBS, 1%FCS, 2 mM EDTA, 0.02% sodium azide). Brilliant stain buffer (BD) was used when more than two Abs were conjugated with BD Horizon Brilliant fluorescent polymer dyes. Ova_257–264_/K^b^, gB_498–505_/K^b^, GP_33–41_/D^b^, GP_276–284_/D^b^, LLO_91–99_/K^d^ and p60_217–225_/K^d^ biotinylated monomers (1 mg/mL) obtained from the NIH tetramer Core Facility, were conjugated with PE-labeled Streptavidin (1 mg/mL) as follow: 6.4 μL of PE-Streptavidin were added to 10 μL of monomers every 15 min 4 times on ice. Newly generated tetramers (1/400–1/500 dilution) were used to stain cells for 1 h at 4 °C. For transcription factor (TF) intracellular staining, cells were fixed and stained according to the eBioscience Foxp3 Transcription Factor Staining Buffer Set protocol. Data acquisition was done using BD LSR II, FACSAria III or Cytek Aurora flow cytometer. All flow cytometry data were analyzed using FlowJo v9 or v10 software (TreeStar).

#### T cell sorting

CD8^+^ T cells were negatively selected from spleen using anti-CD4, anti-CD11b, anti-MHC II, anti-TER119, anti-B220 and anti-CD19 mAbs (Supplementary Data 5), which all were added and incubated at 5 μg/mL for 30 min at 4 °C. Cells were then washed and incubated with anti-rat Ab magnetic beads at 1 bead/target cell for 40 min at 4 °C (Dynabeads sheep anti-rat IgG, Invitrogen). Cell suspensions were stained with Abs specific for congenic markers. Cells were sorted into 3 mL of complete media (RPMI 1640, 10% FBS, 1% Penicillin/Streptomycin, 55 μM β-mercaptoethanol, 1 mM Sodium Pyruvate, 1X Glutamax, 1X non-essential amino acids) using a 4 laser BD FACS Aria III cell sorter. For RNA-seq experiments, cells were directly sorted into 1X lysis buffer (Takara Bio USA).

### In vivo mouse assays

#### Adoptive T cell transfers

Two thousand *Il2ra*^*mut/mut*^ *Cd45.1*^*+/+*^ TdT^+^ OT-I cells and 2000 *Cd45.2*^*+/+*^ TdT^+^ OT-I cells from blood or spleen were prepared in PBS and transferred i.v. into *Cd45.1*^*+/−*^ WT B6 naïve recipient mice. The next day, mice were immunized as indicated above and in the figure legends. For memory cells, 1000 OT-I memory cells of each genotype from immunized mouse spleens, were negatively selected (see below) and flow-sorted (Aria III) based on expressed congenic markers and TdT expression. Sorted OT-I memory cells were mixed at a 1:1 ratio in 200 μl and immediately transferred to *Cd45.1*^*+/−*^ WT B6 naïve recipient mice that were challenged the next day with indicated microbial pathogen.

#### Mixed bone marrow chimera

BM cells were obtained from flushing femurs with complete RPMI media with 10% FCS. Recipient mice were lethally irradiated with 1,200 rads before immediate reconstitution with 5 × 10^6^ BM cells from WT *Cd45.1*^*+/+*^, *Il2ra*^*mut/mut*^ *Cd45.1*^*+/−*^ and *Il2ra*^*−/−*^ *Cd45.2*^*+/+*^ at a 1:1:1 ratio. Mice were placed under antibiotics for 2 weeks and reconstitution ratios were checked by FACS 4–6 weeks later before immunization experiments.

#### IL-2/anti-IL-2 mouse rescue model

Mice adoptively transferred with *Il2ra*^*mut/mut*^ and WT OT-I cells were injected i.p. with a solution of 1.5 μg recombinant mouse IL-2 (rIL-2, preprotech) mixed to 50 μg anti-IL-2 (clone S4B6) as previously described^32^ every day for 3 or 6 consecutive days post *Lm*-Ova infection. rIL-2 and the anti-IL-2 Ab were incubated rotating at 4 °C for 1 h prior to i.p. injection in a total volume of 300 μL of PBS per mouse.

#### In vivo blocking of antigen presentation

Mice adoptively transferred with *Il2ra*^*mut/mut*^ and WT OT-I cells were injected with 500 *μ*g of anti-K^b^/Ova_257–264_ 25-D1.16 mAb per mouse 2 days after immunization with *Lm*-Ova. CD11c^+^ cells were depleted in mice expressing the diphtheria toxin receptor (DTR) under the CD11c/Itgax promoter upon intraperitoneal (i.p.) injection of 4 ng/g of mouse body weight of diphtheria toxin (DT, Calbiochem) 12 h prior to the targeted CD11c^+^ cell depletion time as indicated in the relevant figure legends. Depletion efficiency was confirmed by FACS on 1–2 extra mice in each independent experiment and by monitoring OT-I cell expansion 7 days post immunization.

#### Cell Trace Violet (CTV) or CFSE labeling

Purified naïve OT-I, gBT, P14, or L9.6T cells were stained with 1–5 μM of CTV or CFSE (Invitrogen) according to the manufacturer’s protocol. 5 × 10^4^ CTV-labeled cells were injected i.v. to congenically distinct recipient mice. Cell proliferation of CTV-labeled cells was analyzed by flow-cytometry based on CTV fluorescence intensity dilution among cells.

#### Intracellular calcium accumulation

Splenocytes were loaded with 2.5 μM Fluo3am (ThermoFisher) in PBS by incubating at 25 °C for 30 min. Wells were then washed with indicator-free media and stained for flow cytometry analysis.

#### Cell-cycle analysis

Following F_c_-block and cell-surface staining, splenocytes were fixed and stained with anti-Ki67 mAb and Live/Dead Aqua (ThermoFisher) to quantify DNA as described^52^.

### Transcriptomic profiling of memory T cells

#### Microarrays

50,000 CFSE-labeled OT-I cells were adoptively transferred in recipient mice subsequently infected with *Lm*-Ova N4, T4 or A8, and 3 days later 10,000 CFSE^low^ (divided) OT-I cells were flow-sorted based on congenic markers after enrichment for CD8^+^ T cells (using negative selection, as described above). Pelleted cells were stored in 700 μl of TRIzol (Life Technologies) at −80C until RNA extraction. Total RNA was extracted using the RNAeasy Micro kit with RNase-Free DNase Set (Qiagen) according to the manufacturer protocol. The quality score and quantity of purified RNA was assessed with a Bioanalyzer RNA 6000 Pico Chip (Agilent). Total RNA was then converted to cDNA, amplified and hybridized to Affymetrix Mouse Transcriptome Array 1.0 Pico. Raw CEL files were preprocessed and normalized using Affymetrix Expression Console (version 1.4.1.46) and resulting data were analyzed with the Affymetrix Transcriptome Analysis Console (version 3.1.0.5). We calculated fold-differences between experimental groups and tested significance using one-way ANOVA (unpaired). Significantly up and downregulated genes were defined with at least a 1.5 fold expression difference and a *p*-value ≤ 0.05. Over-representation of biological process (BP) gene ontology (GO) terms was calculated using DAVID 6.8 and visualized in scatterplots using ggplot2 in R.

#### RNA-seq

One thousand memory OT-I cells were flow-sorted based on congenic markers after enrichment for CD8^+^ T cells (as described above). cDNA was synthesized and amplified directly from intact cells using SMART-Seq v4 Ultra Low Input RNA Kit for Sequencing (Takara Bio USA) according to the manufacturer protocol. The quality score and quantity of purified cDNA was assessed with Qubit dsDNA HS Assay kit (Life technologies) and Bioanalyzer High Sensitivity DNA assay (Agilent). Illumina cDNA library was prepared using Nextera XT DNA Library Preparation Kits according to manufacturer protocol. The quality score and quantity of purified cDNA library was assessed with Qubit dsDNA HS Assay kit (Life technologies) and Bioanalyzer High Sensitivity DNA assay (Agilent). The library samples were performed on Illumina HiSeq (2X150bp and dual index configuration) by Genewiz Inc. Reads were aligned to the Mouse reference mm10 using STAR aligner (v2.4.2a)^53^. Quantification of genes annotated in Gencode vM5 were performed using featureCounts (v1.4.3) and quantification of transcripts using Kallisto (v0.46.1). QC was collected with Picard (v1.83) and RSeQC^54^ (http://broadinstitute.github.io/picard/; v2.6.5). Normalization of feature counts was done using the DESeq2 package (v1.10.1). Differentially expressed genes were identify using negative binomial distribution as implemented in DESeq 2 (R package). Significantly up and downregulated genes were defined with at least a 1.5 fold expression difference and an adjusted *p*-value ≤ 0.05.

### Epigenetic profiling of memory T cells

We performed ATAC-seq analysis on two biological replicates per group as previously described^55^. Briefly, 15,000–50,000 OT-I memory cells were flow-sorted from negatively enriched splenocytes from host mice adoptively transferred with WT or *Il2ra*^*mut/mut*^ OT-I cells and immunized with *Lm*-Ova N4 or T4. Then nuclei were isolated using a solution of 10 mM Tris-HCl, 10 mM NaCl, 3 mM MgCl_2_, and 0.1% IGEPAL CA-630. Immediately following nuclei isolation, the transposition reaction was conducted using Tn5 transposase and TD buffer (Illumina) for 45 min at 37 °C. Transposed DNA fragments were purified using Qiagen Mini-Elute Kit and PCR amplified using NEB Next High Fidelity 2x PCR master mix (New England Labs) with dual indexes primers (Illumina Nextera). Genomic Alignment of sequencing reads; for all sequenced data, paired-end reads were trimmed for adaptors and removal of low-quality reads using Trim_galore (v0.3.7). Trimmed reads were mapped to the Mus Musculus genome (mm10 assembly) using Picard (v1.92). Reads were then filtered to exclude mitochondrial DNA or duplicates using samtools (v0.1.19). For peak calling, all positive-strand reads were shifted 4 bp downstream to center the reads on the transposon binding event. We used MACS2 (v2.1.0) at a *p*-value of 0.01. Irreproducible discovery rate (IDR) calculations using scripts provided by the ENCODE project (https://www.encodeproject.org/software/idr/; v2.0.2 and v2.0.3) were performed on all pairs of replicates, keeping only reproducible peaks showing an IDR value of 0.05 or less. For peak annotation and analysis, peak assignment was done using ChipSeeker (v1.24.0)^56^. Promoter regions were defined as peaks that overlapped a region that was + /- 3 kb from the transcriptional start site (TSS). Intragenic (intronic and exonic) peaks were defined as any peak that overlapped with annotated intronic and exonic peaks, respectively, based on the annotation database. Intergenic peaks were defined as any non-promoter or non-intragenic peaks and were assigned to the gene of the nearest TSS based on the distance from the start of the peak. Conserved and unique open chromatin regions (OCRs) were defined using the bedTools intersect function (v2.29.1) at different level of comparison as represented in Fig. 4b. First level of comparison allowed us to identify unique (non-overlapping) peaks between WT N4 and WT T4 (A); MUT N4 and MUT T4 (B); WT N4 and MUT N4 (X); WT T4 and MUT T4 (Y). Similar approach was used in a second level analysis where we identified conserved (overlapping) peaks between A and B (TCR controlled OCRs), overlapping peaks between X and Y (IL-2 controlled OCRs) and peaks unique of A, B, X, Y (OCRs controlled by both TCR and IL-2). The 3 master lists of peaks, TCR, IL-2 and TCR + IL-2, were used for further analysis after filtering for redundant peaks. De novo analysis and known-motif analysis on OCRs were performed using HOMER (v4.4)^57^ with the findMotifsGenome.pl function. Likelihood of TF binding among unique peaks was evaluated using FIMO tool (4.11.1)^58^. Prior to motif analysis, for overlapping peaks (TCR and IL-2), peaks coordinates were adjusted to cover the full region represented by peak 1 and peak 2 using for each peak the extremes start and stop coordinates. We assessed variability among the ATAC-seq datasets by performing Pearson correlation across samples only considering overlapping peaks across conditions. Over-representation of BP GO terms was calculated using PANTHER and visualized in scatterplots using ggplot2 in R.

### Statistics

Statistical significance was calculated using paired or unpaired Student’s *t* tests with GraphPad Prism software and two-tailed *p* values are given as (*) *p* < 0.1; (**) *p* < 0.01; (***) *p* < 0.001; (****) *p* < 0.0001 and (ns) *p* > 0.1. All *p* values of 0.05 or less were considered significant and are referred to as such in the text.

### Reporting summary

Further information on research design is available in the Nature Research Reporting Summary linked to this article.

## Supplementary information

Supplementary Information

Peer Review File

Description of Additional Supplementary Files

Supplementary Data 1

Supplementary Data 2

Supplementary Data 3

Supplementary Data 4

Supplementary Data 5

Reporting Summary

## Data Availability

The accession number for the ATAC-seq and RNA-seq data reported in this paper is GEO: GSE152394. All other data are available in the main text or the Supplementary Information. Source data are provided with this paper.

## Source data

Source Data
