# Supplementary material for: T cell receptor and IL-2 signaling strength control memory CD8+ T cell functional fitness via chromatin remodeling
Source: Nat Commun. 2022 Apr 26;13:2240. doi: 10.1038/s41467-022-29718-2 (PMC9042912; doi:10.1038/s41467-022-29718-2)
Supplement: Supplementary file 1 — Supplementary Information [file 41467_2022_29718_MOESM1_ESM.pdf]

**Supplementary Information**

**Supplementary Figures 1-11**

**Supplementary Data 1. GO pathways for Figure 1c and Microarrays for Figure 1d**

**Supplementary Data 2. Transcriptomic analysis of resting OT-I memory cells for Figures 5a and Supplementary Figure 5**

**Supplementary Data 3. OCR TCR, IL-2 and TCR+IL-2**

**Supplementary Data 4. GO pathways ATAC-seq comparisons for Figure 6d**

**Supplementary Data 5. OCRs in promoters of selected genes and FIMO TF binding analysis for Figure 7**

**Supplementary Table 1. Cytek FACS Panel**

**Supplementary Table 2. Table for antibodies used**

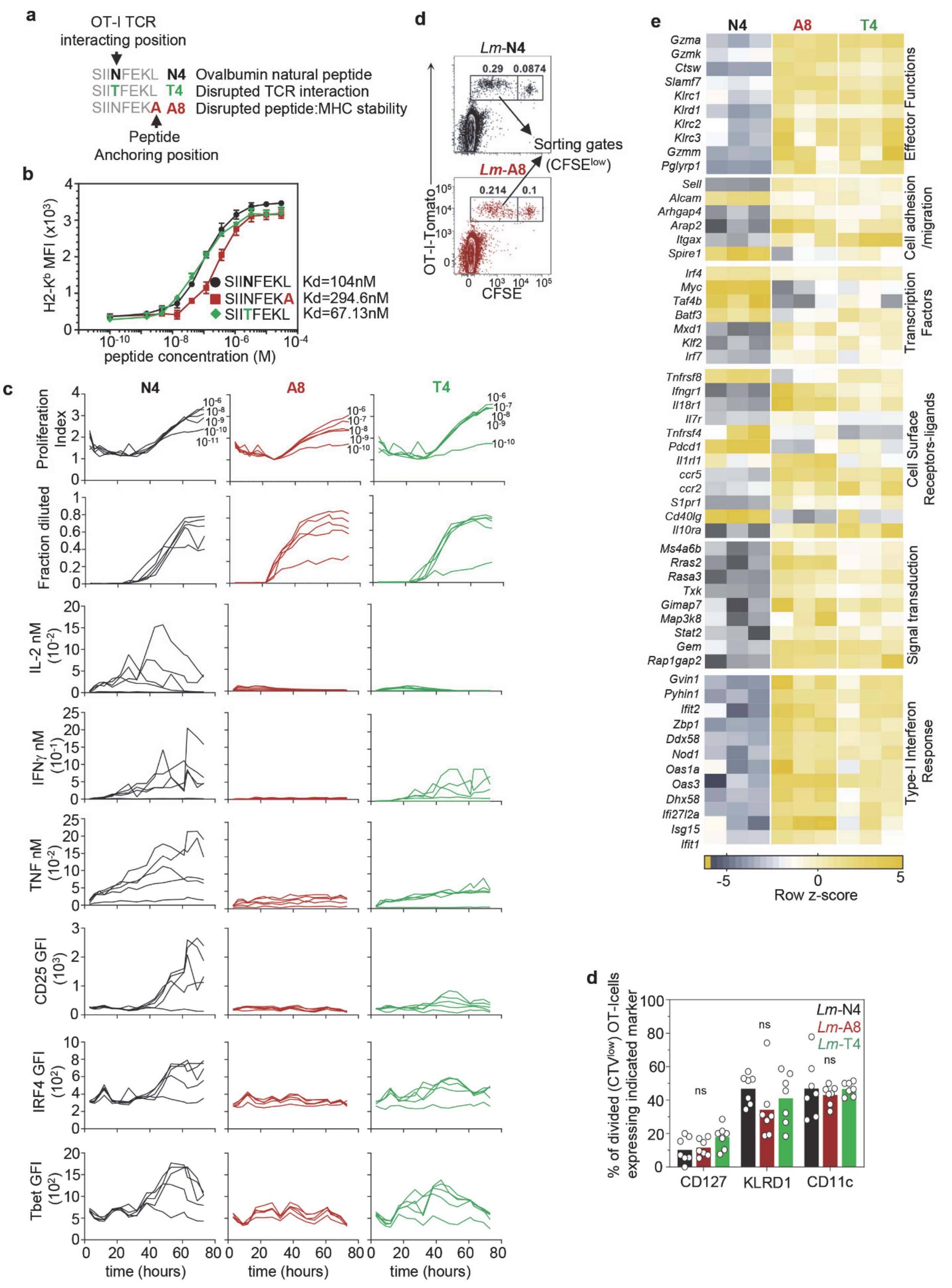

a
OT-I TCR interacting position
SIINFEKL N4 Ovalbumin natural peptide
SIITFEKL T4 Disrupted TCR interaction
SIINFEKA A8 Disrupted peptide:MHC stability
Peptide Anchoring position
b
H2-K$^b$ MFI (x10$^3$)
peptide concentration (M)
SIINFEKL Kd=104nM
SIINFEKA Kd=294.6nM
SIITFEKL Kd=67.13nM
c
N4
A8
T4
Proliferation Index
Fraction diluted
IL-2 nM (10$^{-2}$)
IFNγ nM (10$^{-1}$)
TNF nM (10$^{-2}$)
CD25 GFI (10$^3$)
IRF4 GFI (10$^2$)
Tbet GFI (10$^2$)
time (hours)
d
Lm-N4
Lm-A8
0.29
0.0874
0.214
0.1
Sorting gates (CFSE$^{low}$)
OT-I-Tomato
CFSE
e
N4
A8
T4
Gzma
Gzmk
Ctsw
Slamf7
Klrc1
Klrd1
Klrc2
Klrc3
Gzmm
Pglyrp1
Effector Functions
Sell
Alcam
Arhgap4
Arap2
Itgax
Spire1
Cell adhesion /migration
Irf4
Myc
Taf4b
Batf3
Mxd1
Klf2
Irf7
Transcription Factors
Tnfrsf8
Ifngr1
Il18r1
Il7r
Tnfrsf4
Pdcd1
Il1rl1
ccr5
ccr2
S1pr1
Cd40lg
Il10ra
Cell Surface Receptors-ligands
Ms4a6b
Rras2
Rasa3
Txk
Gimap7
Map3k8
Stat2
Gem
Rap1gap2
Signal transduction
Gvin1
Pyhin1
Ifit2
Zbp1
Ddx58
Nod1
Oas1a
Oas3
Dhx58
Ifi27l2a
Isg15
Ifit1
Type-I Interferon Response
Row z-score
d
% of divided (CTV$^{low}$) OT-Icells expressing indicated marker
Lm-N4
Lm-A8
Lm-T4
ns
CD127
KLRD1
CD11c

**Supplementary Figure 1. TCR signaling strength affects early $CD8^+$ T cell activation program.** (**a**) Ova-derived SIINFEKL and its APLs. (**b**) H2-$K^b$ RMA-S stabilization assay comparing Ova N4, A8 and T4 across 2 independent replicate experiments. Data are presented as average MFI values and error bars indicate SEM. Calculated Kd for each peptide is shown. (**c**) Graphs show *in vitro* stimulation of OT-I cells cultured with various concentrations of Ova N4, A8 and T4. (**d**) Representative FACS dot plots showing $CFSE^{low}$ N4- and A8-primed OT-I cells 3 days post-infection subsequently sorted for microarray analysis. (**e**) Heat map of selected genes grouped under the indicated categories and for which expression is significantly different between N4-, A8- and T4-primed OT-I cells. (**f**) Graphs show proportion of $CTV^{low}$ (divided) OT-I cells expressing indicated markers 3 days post immunization with *Lm*-Ova N4, A8 or T4 across 2 independent replicate experiments (n=7 mice per group). Each symbol represents 1 mouse and ns indicates no statistical significance using two-tailed unpaired Student's *t* test corrected for multiple comparison using a 1% False Discovery Rate (FDR).

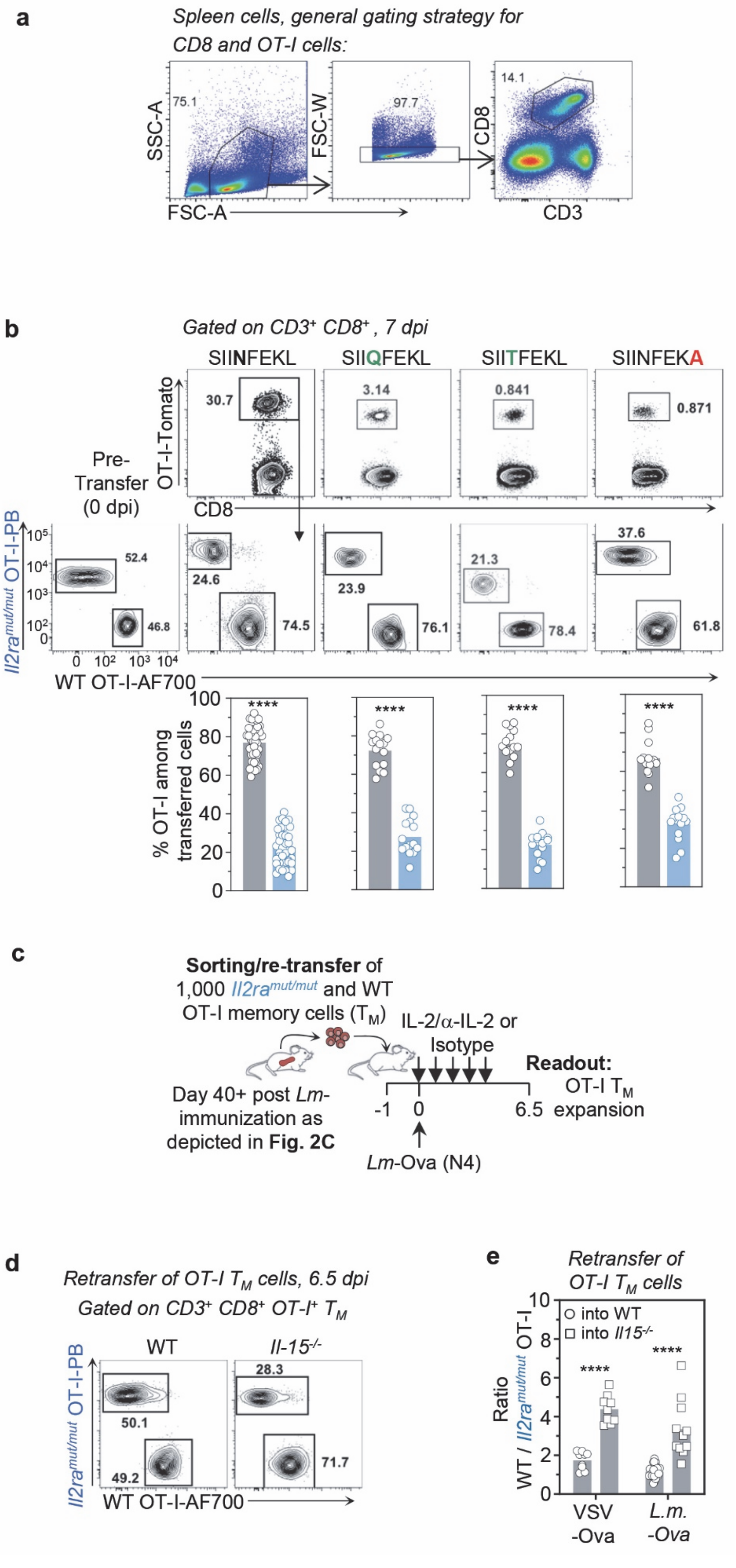
a
Spleen cells, general gating strategy for CD8 and OT-I cells:
SSC-A
FSC-A
75.1
FSC-W
97.7
CD8
CD3
14.1
b
Gated on CD3+ CD8+, 7 dpi
SIINFEKL
SIIQFEKL
SIITFEKL
SIINFEKA
OT-I-Tomato
CD8
30.7
3.14
0.841
0.871
Pre-Transfer (0 dpi)
Il2ra mut/mut OT-I-PB
WT OT-I-AF700
52.4
46.8
24.6
74.5
23.9
76.1
21.3
78.4
37.6
61.8
% OT-I among transferred cells
****
c
Sorting/re-transfer of 1,000 Il2ra mut/mut and WT OT-I memory cells (TM)
IL-2/α-IL-2 or Isotype
Readout: OT-I TM expansion
Day 40+ post Lm-immunization as depicted in Fig. 2C
-1
0
6.5
Lm-Ova (N4)
d
Retransfer of OT-I TM cells, 6.5 dpi
Gated on CD3+ CD8+ OT-I+ TM
WT
Il-15-/-
Il2ra mut/mut OT-I-PB
WT OT-I-AF700
50.1
49.2
28.3
71.7
e
Retransfer of OT-I TM cells
into WT
into Il15-/-
Ratio WT / Il2ra mut/mut OT-I
****
VSV -Ova
L.m. -Ova

**Supplementary Figure 2. CD8$^+$ T cells primed with weak TCR and IL-2 signals failed to expand competitively during primary challenge.** (**a**) General gating strategy for CD8$^+$ T cells and OT-I cells. (**b**) Representative FACS dot plots of adoptively transferred *Il2ra*$^{mut/mut}$ versus WT OT-I cells 7 days post primary infection with *Lm* expressing N4, Q4, T4 or A8. Bar graphs show the relative pooled frequency of expanded OT-I cells across >5 independent replicate experiments (n=14-42 mice per group; each data point represents 1 mouse). *p*-values are indicated when relevant with ****$p < 0.0001$ using two-tailed paired Student's *t* test. (**c**) Schematic of experimental design complementing Figure 2C. (**d**) Representative FACS dot plots of relative proportions of *Il2ra*$^{mut/mut}$ and WT OT-I memory cells from Figure 2E. (**e**) Bar graph shows the ratio of WT versus *Il2ra*$^{mut/mut}$ OT-I memory cells adoptively transferred in either WT or *Il15*$^{-/-}$ recipient mice subsequently infected with indicated pathogens across 3 independent replicate experiments (n=9-24 mice per group; each data point represents 1 mouse). *p*-values are indicated when relevant with ****$p < 0.000044$ and 0.000052 using two-tailed unpaired Student's *t* test.

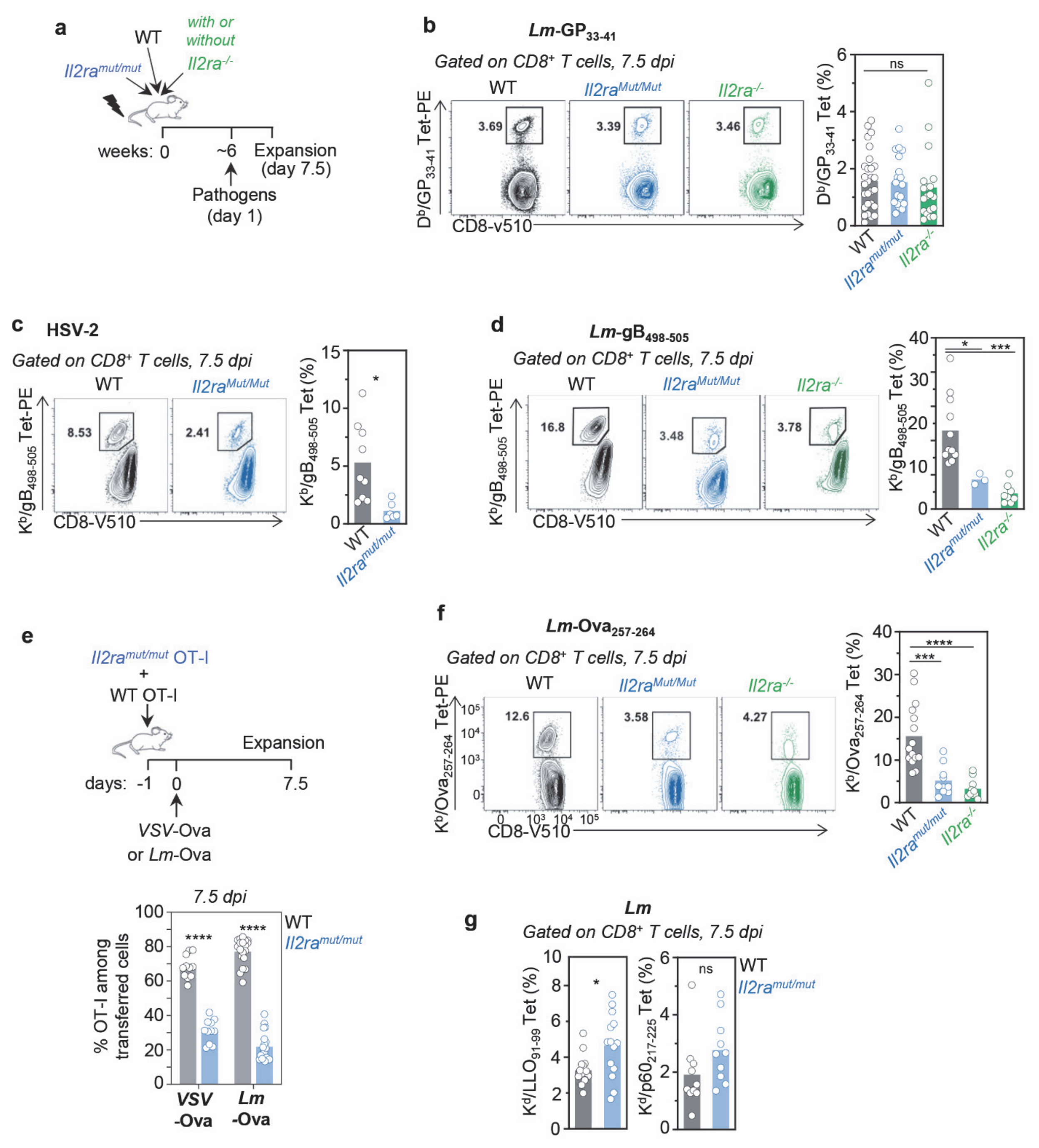

**Supplementary Figure 3. Analysis of WT and IL-2-disrupted pathogen-specific CD8⁺ T cell primary responses in response to various epitopes and challenge infections. (a)** Schematic of BM chimera experimental setup. Irradiated hosts were reconstituted with BM harvested from WT, *Il2ra*$^{mut/mut}$ and/or *Il2ra*$^{-/-}$ mice (ratio 1:1:1), and 6 weeks later, infected with indicated pathogens. **(b**) Representative FACS plots of WT and *Il2ra*$^{mut/mut}$ GP33-41/D$^{b}$ Tet$^{+}$ CD8$^{+}$ T cells

in BM chimeras infected by *Lm*-GP33-41. Bar graphs show the relative frequencies of *Il2ra*$^{mut/mut}$ versus WT Db/GP33-41-specific Tet$^{+}$ CD8$^{+}$ T cells 7.5 days post primary infection in 2-4 independent replicate experiments (n=4-20 mice). **(c)** Representative FACS dot plots of WT and *Il2ra*$^{mut/mut}$ gB498-505/K$^{b}$ Tet$^{+}$ CD8+ T cells in BM chimeras infected by HSV-2. Bar graph shows the relative frequency of *Il2ra*$^{mut/mut}$ versus WT Tet$^{+}$ CD8$^{+}$ T cells 7.5 days post primary infection across 2 replicate experiments (n=9 mice for WT and 6 mice for *Il2ra*$^{mut/mut}$ groups). **(d)** Representative FACS dot plots of WT and *Il2ra*$^{mut/mut}$ gB498-505/K$^{b}$ Tet$^{+}$ CD8$^{+}$ T cells from BM chimeras infected by *Lm*-gB498-505. Bar graph shows the relative frequency of *Il2ra*$^{mut/mut}$ versus WT Tet$^{+}$ CD8$^{+}$ T cells 7.5 days post primary infection across 2 replicate experiments (n=3-11 mice). **(e)** Schematic of experimental setup to examine primary expansion of adoptively transferred *Il2ra*$^{mut/mut}$ and WT OT-I cells. Bar graph shows the relative frequency of *Il2ra*$^{mut/mut}$ and WT OT-I cells 7.5 days post primary infection across 2-3 replicate experiments (n=11 mice for *VSV*-Ova and 19 mice for *Lm*-Ova). **(f)** Representative FACS dot plots of Ova257-264/K$^{b}$ Tet$^{+}$ WT and *Il2ra*$^{mut/mut}$ CD8$^{+}$ T cells in BM chimeras infected by *Lm*-Ova257-264. Bar graph shows the relative frequency of *Il2ra*$^{mut/mut}$ versus WT Tet$^{+}$ CD8$^{+}$ T cells 7.5 days post primary infection across 2-3 replicate experiments (n=14 mice for WT and 7 mice for *Il2ra*$^{mut/mut}$ and *Il2ra*$^{-/-}$). **(g)** Bar graphs show the relative frequency of *Il2ra*$^{mut/mut}$ versus WT indicated Tet$^{+}$ CD8$^{+}$ T cells 7.5 days post primary *Lm* infection of B6-K$^{d}$ mice across 2 replicate experiments (n=10 mice for K$^{d}$/p60217-225 and 14 mice K$^{d}$/LLO91-99). In all panels, each symbol represents 1 mouse and *p* values are indicated when relevant with $^{*}p < 0.05$; $^{**}p < 0.01$; $^{***}p < 0.001$; $^{****}p < 0.0001$; ns, not significant, using two-tailed unpaired Student's *t* test.

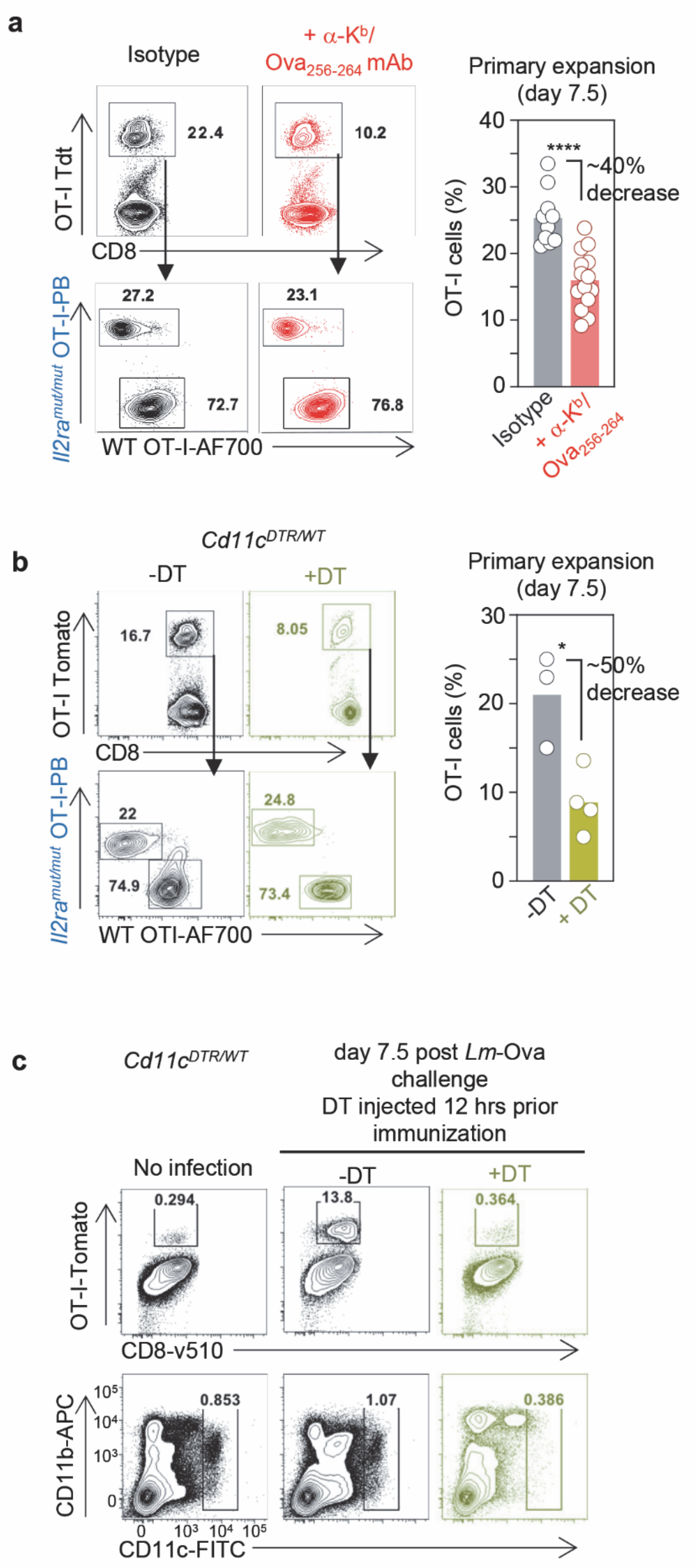

a
Isotype
+ α-Kb/ Ova256-264 mAb
22.4
10.2
OT-I Tdt
CD8
27.2
23.1
72.7
76.8
Il2ramut/mut OT-I-PB
WT OT-I-AF700
Primary expansion (day 7.5)
OT-I cells (%)
****
~40% decrease
Isotype
+ α-Kb/ Ova256-264
b
Cd11cDTR/WT
-DT
+DT
16.7
8.05
OT-I Tomato
CD8
22
24.8
74.9
73.4
Il2ramut/mut OT-I-PB
WT OTI-AF700
Primary expansion (day 7.5)
OT-I cells (%)
*
~50% decrease
-DT
+ DT
c
Cd11cDTR/WT
day 7.5 post Lm-Ova challenge
DT injected 12 hrs prior immunization
No infection
-DT
+DT
0.294
13.8
0.364
OT-I-Tomato
CD8-v510
0.853
1.07
0.386
CD11b-APC
CD11c-FITC

**Supplementary Figure 4. Disrupting antigen presentation impairs primary CD8$^+$ T cell responses.** *Il2ra$^{mut/mut}$* and WT OT-I cells adoptively transferred to naïve WT (**a**) or *Cd11cDTR/WT* (**b, c**) mice were subsequently infected the day after with *Lm*-OvaN4. Antigen presentation was disrupted or not (isotype) by injecting (**a**) anti-MHC-I K$^b$/Ova256-264 mAb or (**b**) diphtheria toxin (DT) 48 hrs post-infection and spleen cells were stained for FACS analysis of OT-I cell expansion of each genotype for the various treatments 7.5 days later. Representative FACS dot plots are shown. Bar graph shows the frequency OT-I cell expansion in anti-K$^b$/Ova256-264 mAb (n= 14 mice) versus isotype-treated (n= 10 mice) or DT-treated (n= 4 mice) versus untreated mice (n= 3 mice in 2-3 replicate experiments. (**c**) Same design as in (B), but with *Il2ra$^{mut/mut}$* and WT OT-I cells adoptively transferred to *Cd11cDTR/WT* recipient mice further injected with diphtheria toxin (DT) 12 hours prior infection to deplete CD11c$^+$ cells. Dot plots show OT-I cell expansion (or lack of) and CD11c$^+$ DC depletion 7.5 days post challenge infection in 1 of 4 representative mice. In all relevant panels, each symbol represents 1 mouse and *p*-values are indicated when relevant with $*p < 0.05$; $**p< 0.01$; $***p< 0.001$; $****p< 0.0001$; ns, not significant, using two-tailed unpaired Student's *t* test.

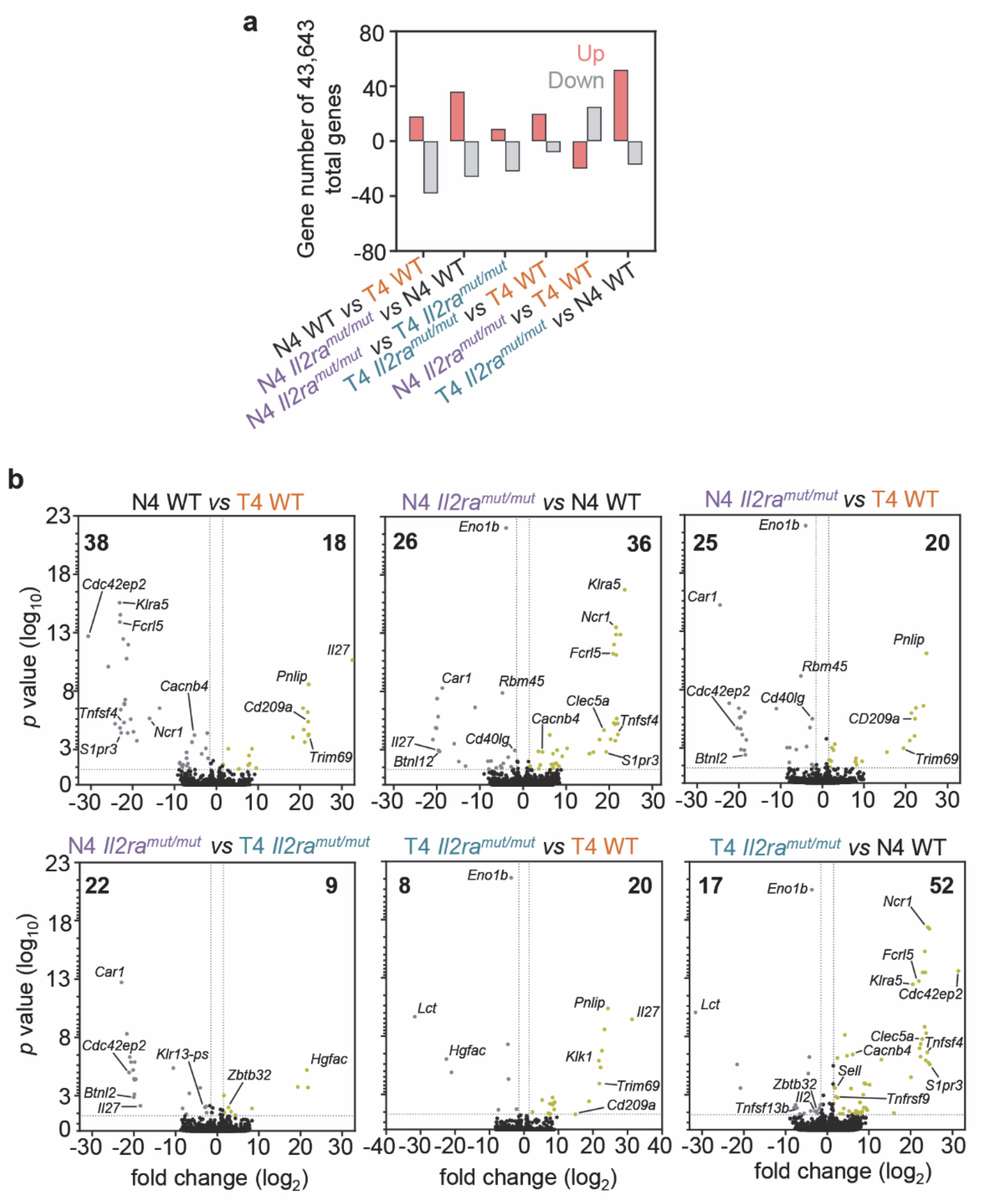


**Supplementary Figure 5. The strength of TCR and/or IL-2 signaling only minimally affects gene expression in resting memory CD8$^{+}$ T cells. (a)** Bar graph shows the number of significantly up- and down-regulated genes in *Il2ra*$^{mut/mut}$ and WT resting OT-I memory cells primed after infection *Lm*-OvaN4 or *Lm*-OvaT4, defined as genes with at least 1.5 fold change, adjusted $p$ value $\leq 0.05$ in each respective comparison. **(b)** Volcano plot of $p$-values versus gene expression fold change in each respective comparison. Significantly up- and down-regulated genes were defined as genes with at least 1.5 fold change, p-value $\leq$ 0.05, and colored gold or gray, respectively. One-way ANOVA statistical test was used.

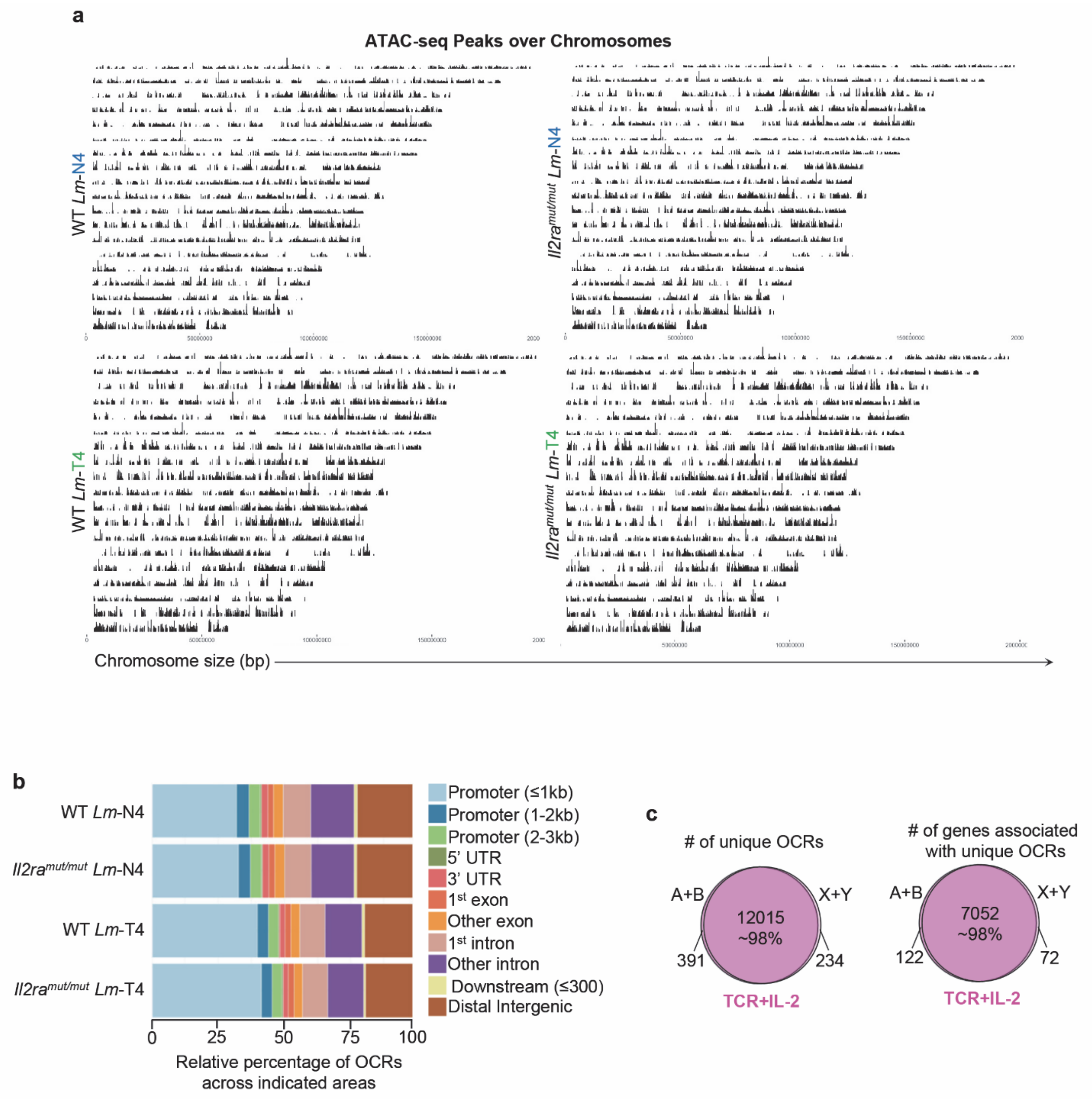


**Supplementary Figure 6. Analysis of chromatin remodeling in OT-I memory cells primed under the various conditions. (a)** Distribution of ATAC-seq peaks across chromosomes in the various resting OT-I memory cells compared. **(b)** Relative OCR proportions across genome areas in *Il2ra*$^{mut/mut}$ and WT resting OT-I memory cells primed after infection *Lm*-OvaN4 or *Lm*-OvaT4. **(c)** Venn diagrams comparing the number of unique OCRs and the number of genes associated with these unique OCRs induced by modulation of TCR+IL-2 signals.

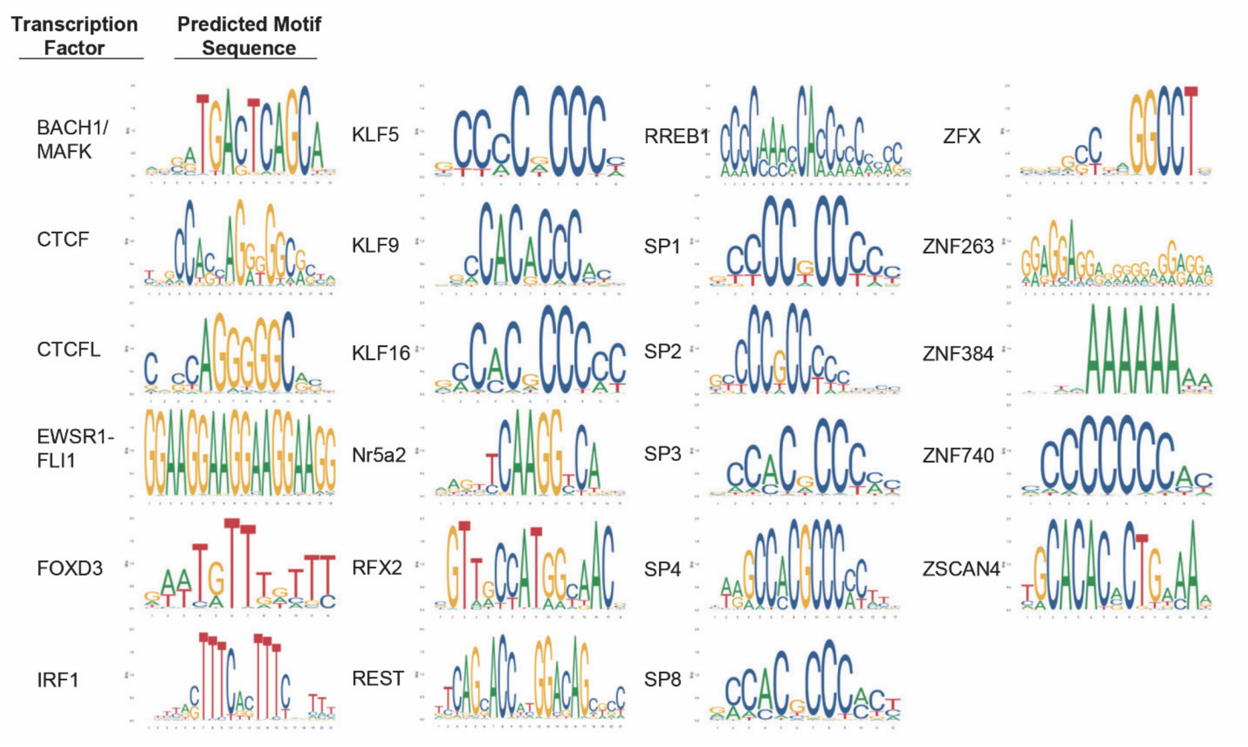


**Supplementary Figure 7. DNA binding motifs for indicated transcription factors.** The JASPAR database was used to recover the predicted DNA binding sequences for listed TFs revealed from the FIMO analysis of the differentially accessible OCRs of Figure 7a genes.

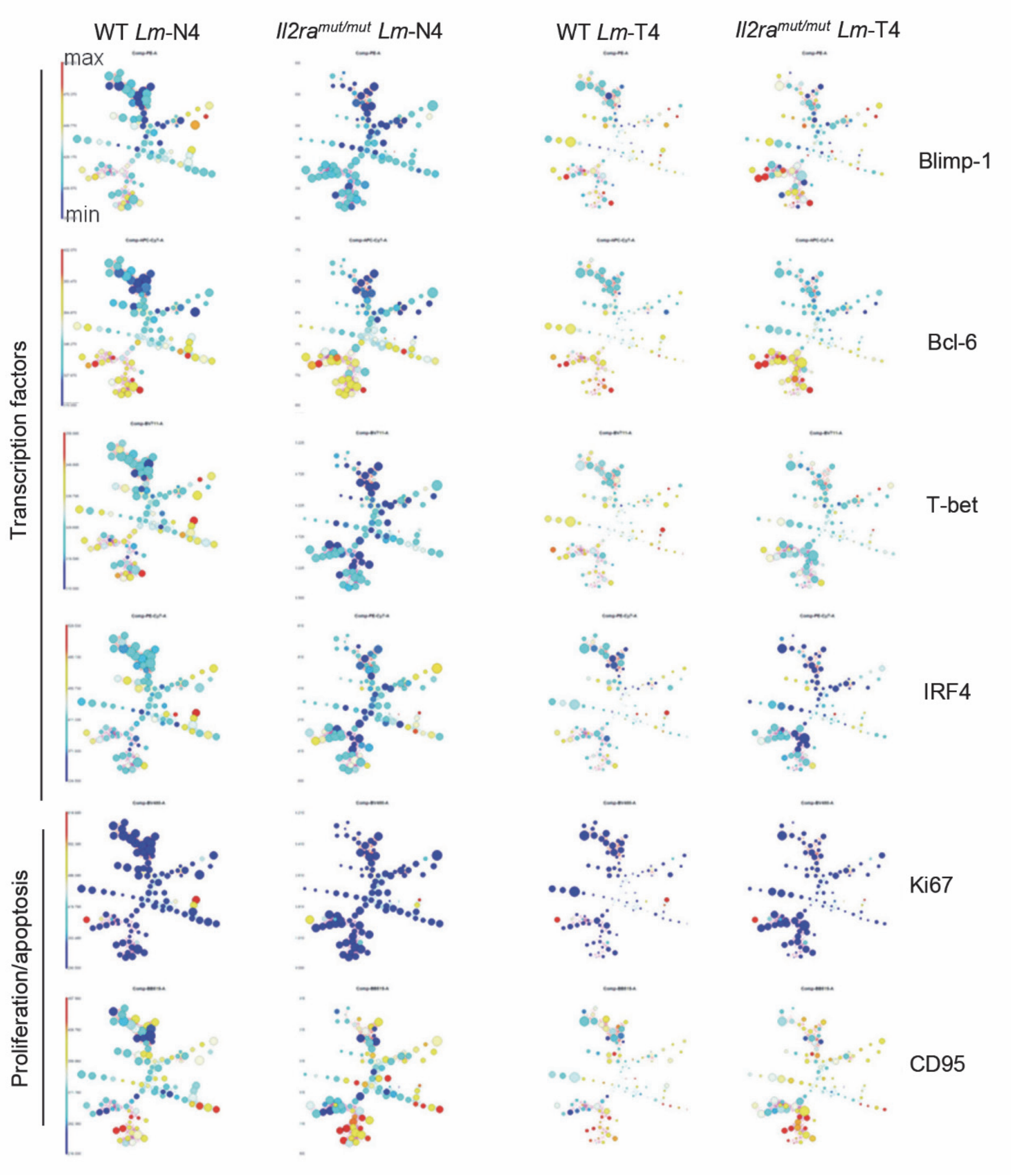


**Supplementary Figure 8. FlowSOM analysis of *Il2ra$^{mut/mut}$* and WT OT-I memory cells primed with either *Lm*-N4 or *Lm*-T4 after staining with a panel of 26 memory cell-relevant markers.** The expression level of each marker within each node is represented in a color scale.

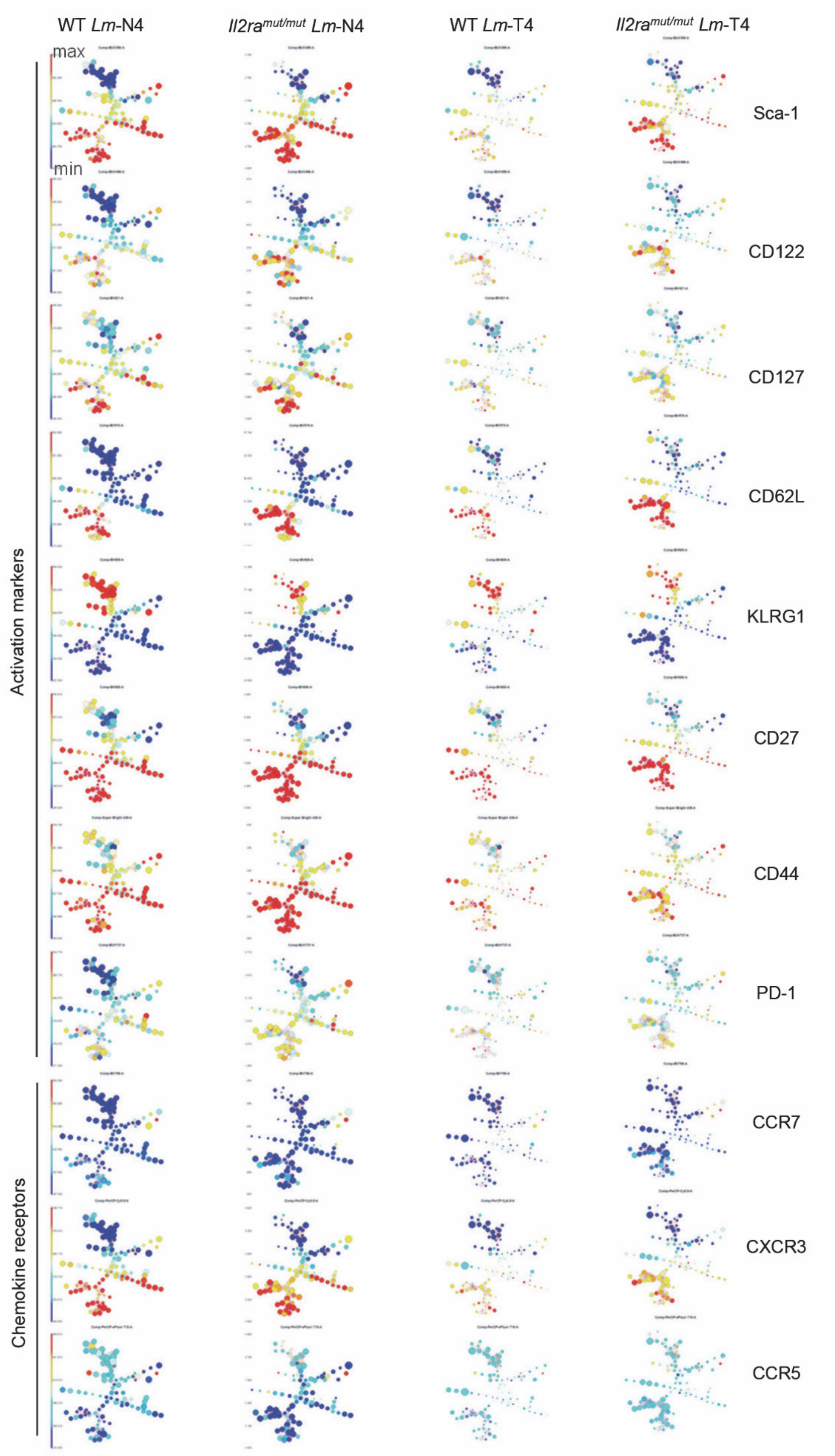
WT Lm-N4
Il2ra^mut/mut Lm-N4
WT Lm-T4
Il2ra^mut/mut Lm-T4
max
min
Activation markers
Chemokine receptors
Sca-1
CD122
CD127
CD62L
KLRG1
CD27
CD44
PD-1
CCR7
CXCR3
CCR5

**Supplementary Figure 9. FlowSOM analysis of *Il2ra*$^{mut/mut}$ and WT OT-I memory cells primed with either *Lm*-N4 or *Lm*-T4 after staining with a panel of 26 memory cell-relevant markers.** The expression level of each marker within each node is represented in a color scale.

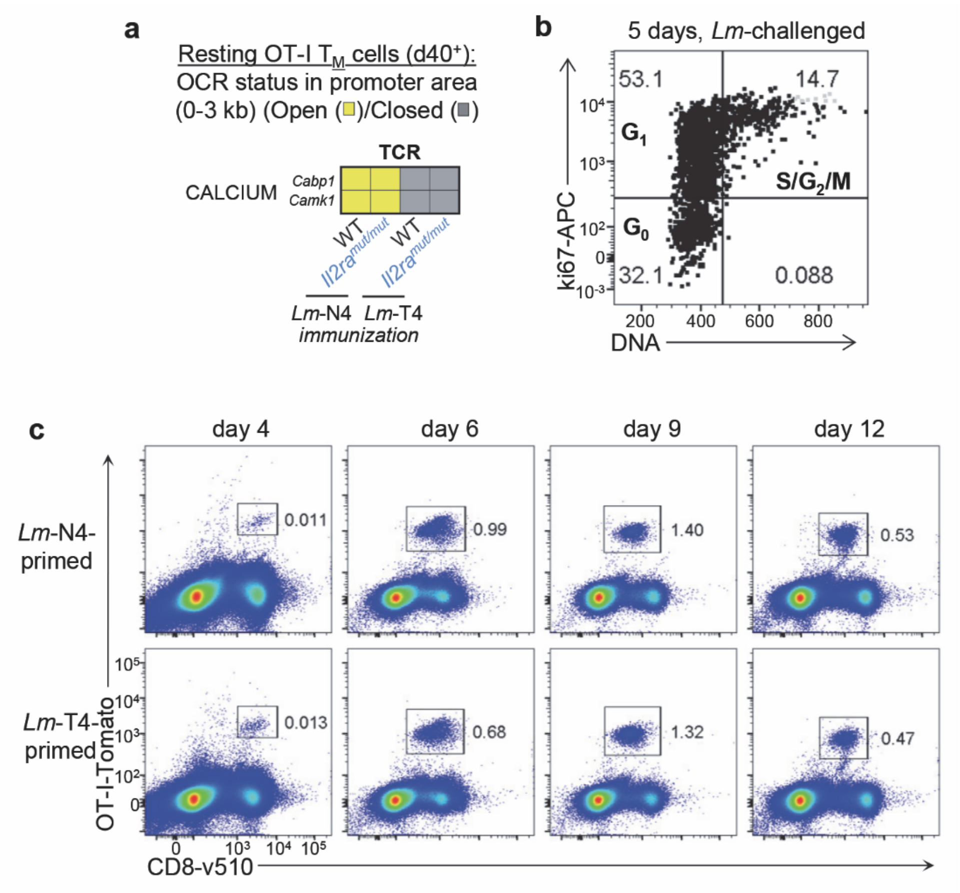


**Supplementary Figure 10. Functional analysis of reactivated memory CD8$^{+}$ T cells.** (**a**) Heat map shows OCRs (yellow) in genes encoding calcium fluxes that were revealed in Figure 6b when only TCR signals were modulated, in the promoter area and close to the TSS (+/- ~3kb). Chromatin region status (open, yellow/closed, grey) in WT and *Il2ra*$^{mut/mut}$ OT-I memory cells primed with *Lm*-N4 or *Lm*-T4 are reported. (**b**) Representative FACS dot plot of cell cycle analysis of splenic CD8$^{+}$ T cells 5 days post *Lm*-immunization, after staining for cell surface CD3, CD8 and intracellular Ki67 and DNA. (**c**) Experimental design is that of Figure 9A, "late reactivation". 40 days post primary infection, 1,000 *Il2ra*$^{mut/mut}$ and WT OT-I memory cells from each priming condition, were FACS-sorted and transferred at a 1:1 ratio to new hosts subsequently infected with *Lm*-OvaN4 the day after. At indicated days, spleen cells were stained to quantify frequencies of global OT-I memory cell expansion among CD8$^{+}$ T cells. Data is a representative dot plot of n=15 mice.

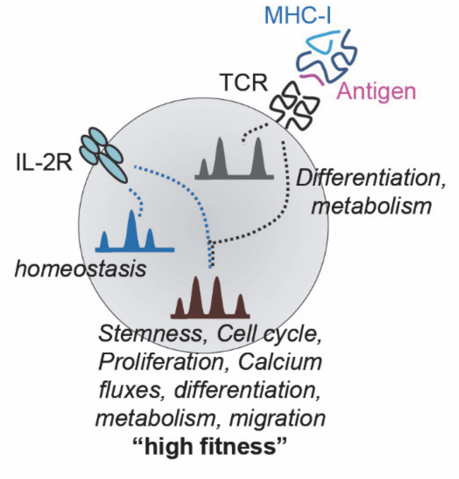


**Supplementary Figure 11. Proposed working model.**
