# Supplementary material for: T cell receptor and IL-2 signaling strength control memory CD8+ T cell functional fitness via chromatin remodeling
Source: Nat Commun. 2022 Apr 26;13:2240. doi: 10.1038/s41467-022-29718-2 (PMC9042912; doi:10.1038/s41467-022-29718-2)
Supplement: Supplementary file 3 — Description of Additional Supplementary Files [file 41467_2022_29718_MOESM3_ESM.pdf]

Description of Additional Supplementary Files

Supplementary Data 1. GO pathways for Figure 1c

Supplementary Data 2. Transcriptomic analysis of resting OT-I memory cells for Figures 5a and Supplementary Figure 5

Supplementary Data 3. OCR TCR, IL-2 and TCR+IL-2

Supplementary Data 4. GO pathways ATAC-seq comparisons for Figure 6d

Supplementary Data 5. OCRs in promoters of selected genes and FIMO TF binding analysis for Figure 7
