# Supplementary material for: T cell receptor and IL-2 signaling strength control memory CD8+ T cell functional fitness via chromatin remodeling
Source: Nat Commun. 2022 Apr 26;13:2240. doi: 10.1038/s41467-022-29718-2 (PMC9042912; doi:10.1038/s41467-022-29718-2)
Supplement: Supplementary file 9 — Reporting Summary [file 41467_2022_29718_MOESM9_ESM.pdf]

Corresponding author(s): Gregoire Lauvau

Last updated by author(s): Feb 10, 2022

Reporting Summary

Nature Portfolio wishes to improve the reproducibility of the work that we publish. This form provides structure for consistency and transparency in reporting. For further information on Nature Portfolio policies, see our Editorial Policies and the Editorial Policy Checklist.

Statistics

For all statistical analyses, confirm that the following items are present in the figure legend, table legend, main text, or Methods section.

n/a	Confirmed	
☐	☒	The exact sample size (*n*) for each experimental group/condition, given as a discrete number and unit of measurement
☐	☒	A statement on whether measurements were taken from distinct samples or whether the same sample was measured repeatedly
☐	☒	The statistical test(s) used AND whether they are one- or two-sided<br>*Only common tests should be described solely by name; describe more complex techniques in the Methods section.*
☒	☐	A description of all covariates tested
☐	☒	A description of any assumptions or corrections, such as tests of normality and adjustment for multiple comparisons
☐	☒	A full description of the statistical parameters including central tendency (e.g. means) or other basic estimates (e.g. regression coefficient) AND variation (e.g. standard deviation) or associated estimates of uncertainty (e.g. confidence intervals)
☐	☒	For null hypothesis testing, the test statistic (e.g. *F*, *t*, *r*) with confidence intervals, effect sizes, degrees of freedom and *P* value noted<br>*Give P values as exact values whenever suitable.*
☒	☐	For Bayesian analysis, information on the choice of priors and Markov chain Monte Carlo settings
☐	☒	For hierarchical and complex designs, identification of the appropriate level for tests and full reporting of outcomes
☐	☒	Estimates of effect sizes (e.g. Cohen's *d*, Pearson's *r*), indicating how they were calculated

Our web collection on statistics for biologists contains articles on many of the points above.

Software and code

Policy information about availability of computer code

Data collection	No software was used to collect data.
Data analysis	Statistical analysis was carried out using GraphPad Prism (v8). All flow cytometry data were analyzed using FlowJo software (TreeStar v9 or v10). Transcriptomic microarray data was preprocessed and normalized with Affymetrix Expression Console (v1.4.1.46) and analyzed with Affymetrix Transcriptime Analysis Console (v3.1.0.5). RNA-sequencing reads were aligned using STAR aligner (v2.4.2a), annotated using Gencode (vM5) and analyzed using Kallisto (v0.46.1), Picard (v1.83), RSeQC (v2.6.5), and R-package DESeq2 (v1.10.1). We identifies differentially expressed genes using negative binomial distribution in DESeq 2 (v1.10.1). For epigenomic profiling, ATAQ-Seq data was processed with Trim_galore (v0.3.7) , Picard (v1.92), and samtools (v0.1.19). Peak calling was performed with MACS2 (v2.1.0) and Irreproducible discovery rate (IDR) was calculated with ENCODE Project (https://www.encodeproject.org/software/idr/; v2.0.2 and v2.0.3) to ensure reproducibility. Peak annotations were done using ChipSeeker (v1.24.0) and chromatin accessibility was defined using bedTools (v2.29.1). Known-motif analysis was performed using the findMotifsGenome.pl function in HOMER (v4.4). Likelihoood of TF binding to accessible chromatin regions was determined using FIMO (v4.11.1). Gene ontology was analyzed using DAVID (v6.8) and PANTHER (v14) and scatter plots were visualized using ggplot2 in R. Full details are described in the Methods section of this paper.

For manuscripts utilizing custom algorithms or software that are central to the research but not yet described in published literature, software must be made available to editors and reviewers. We strongly encourage code deposition in a community repository (e.g. GitHub). See the Nature Portfolio guidelines for submitting code & software for further information.

Data

Policy information about availability of data

All manuscripts must include a data availability statement. This statement should provide the following information, where applicable:

- Accession codes, unique identifiers, or web links for publicly available datasets
- A description of any restrictions on data availability
- For clinical datasets or third party data, please ensure that the statement adheres to our policy

The GEO accession number for the ATAC-seq and RNA-seq data reported in this paper GSE152394 (https://www-ncbi-nlm-nih-gov.ezproxy.u-pec.fr/geo/query/acc.cgi?acc=GSE152394). All data is available in the main text or the supplementary materials.

Field-specific reporting

Please select the one below that is the best fit for your research. If you are not sure, read the appropriate sections before making your selection.

[x] Life sciences [ ] Behavioural & social sciences [ ] Ecological, evolutionary & environmental sciences

For a reference copy of the document with all sections, see nature.com/documents/nr-reporting-summary-flat.pdf

Life sciences study design

All studies must disclose on these points even when the disclosure is negative.

Sample size	No sample size calculation was performed. Experiments shown represent at least 2 or 3 independent biological replicates with 3-5 mice for each experiment. Results were compared between experimental groups with indicated statistical tests. . A power analysis and sample size estimation tool is used to calculate group sizes63. Based on preliminary data, we determined that groups of 6-12 mice were needed overall to reject the null hypothesis.
Data exclusions	Approximately 5% of experimental samples were clear outliers exhibiting a >10 fold difference compared to the range of the other samples in the experiment. Such samples were excluded from our analyses but any other samples with variation below 10 fold were included as also shown in the bar graphs.
Replication	All experiments shown in this study were replicated independently with groups of 3-5 female or male mice of 6-10 weeks of age at least 2 or 3 times at different points in time throughout the study. RMA-S experiments were carried out multiple times at the hands of multiple researchers. Sequencing data included three independent biological replicates to ensure validity of observations. We observed the occasional outlier, however all attempts at replication remained successful.
Randomization	Mice from each relevant genotype we investigate were bred and randomly assigned to different experimental groups. Age and gender matched mice between 6-10 weeks were used across groups. For the few non-mouse experiments that involved cell lines, cells were grown from independent batches between replicates and and equally distributed across the different experimental conditions.
Blinding	Experimental groups were not blinded to the experimenters in charge of the project as it was difficult to organize but raw data generated was interpreted by the PI independently.

Reporting for specific materials, systems and methods

We require information from authors about some types of materials, experimental systems and methods used in many studies. Here, indicate whether each material, system or method listed is relevant to your study. If you are not sure if a list item applies to your research, read the appropriate section before selecting a response.

Materials & experimental systems

n/a	Involved in the study	
[ ]	[x]	Antibodies
[ ]	[x]	Eukaryotic cell lines
[x]	[ ]	Palaeontology and archaeology
[ ]	[x]	Animals and other organisms
[x]	[ ]	Human research participants
[x]	[ ]	Clinical data
[x]	[ ]	Dual use research of concern

Methods

n/a	Involved in the study	
[x]	[ ]	ChIP-seq
[ ]	[x]	Flow cytometry
[x]	[ ]	MRI-based neuroimaging

Antibodies

Antibodies used	All information is provided in the Supplementary Table 7.

Validation	The antibodies used in this study come from commercial manufacturers as specified in Supplementary Table 7. Antibodies were used in accordance with manufacturer recommendations. All antibodies used were validated for appropriate species reactivity (i.e. mouse) and for use in flow cytometry applications. Optimal concentrations were determined empirically. Specified dilutions, clones, and manufacturers are indicated in Supplementary Table 7. Antibody staining procedures are specified in the Method section of the paper.

Eukaryotic cell lines

Policy information about cell lines

Cell line source(s)	The only cell line used in this study was RMA-S cell line which was obtained from Dr. Steve Porcelli (Albert Einstein College of Medicine).
Authentication	The cell line was not authenticated.
Mycoplasma contamination	RMA-S cells were confirmed mycoplasma negative.
Commonly misidentified lines (See ICLAC register)	No commonly misidentified lines were used in this study.

Animals and other organisms

Policy information about studies involving animals; ARRIVE guidelines recommended for reporting animal research

Laboratory animals	All mice were bred in our SPF facility at the Albert Einstein College of Medicine. Housing conditions included a 12 hour light/dark cycle, a 70 degree Fahrenheit room temperature set point, and monitored humidity (30-70% range) . For all experiments, we used 6-10 week old male and female mice that were age- and sex-matched. Females and males were used equally to prevent any gender biases in results. The study has ethical approval by the Albert Einstein College of Medicine, under protocol numbers 20180506 and 00001375. We used wild-type (WT) C57BL/6J (B6) , congenic CD45.1+/+ (JAX#002014), OT-I+ (JAX#003831), P14 (JAX#004694, backcrossed to B6/DBA/2>6 times), CD11c-DTR+/- (JAX#004509) and Rosa26-Actin-tomato-stoploxP/loxP-GFP (TdT)(JAX#007576) all purchased from the Jackson labs. Il15-/- (stock#4269) mice were purchased from Taconic farms. We also bred gBT-I (gift Dr. Carbone), L9.6+ Kd+55, Il2ramut/mut and Ifnar-/- mice (gift Dr. Kohlmeier, Emory Vaccine Center). All mice are on the B6 genetic background unless otherwise specified.
Wild animals	The study did not involve wild animals.
Field-collected samples	The study did not involve field-collected samples.
Ethics oversight	This study was carried out in strict accordance with the recommendations by the animal use committee at the Albert Einstein College of Medicine.

Note that full information on the approval of the study protocol must also be provided in the manuscript.

Flow Cytometry

Plots

Confirm that:

- [x] The axis labels state the marker and fluorochrome used (e.g. CD4-FITC).
- [x] The axis scales are clearly visible. Include numbers along axes only for bottom left plot of group (a 'group' is an analysis of identical markers).
- [x] All plots are contour plots with outliers or pseudocolor plots.
- [x] A numerical value for number of cells or percentage (with statistics) is provided.

Methodology

Sample preparation	Spleens or lymph nodes were dissociated on a nylon mesh. Cell suspensions were treated with red blood cells lysis buffer (0.83% NH4Cl vol/vol). Blood was harvested into heparin tubes and RBC lysed. Cell suspensions were incubated with 2.4G2 Fc Block and stained with fluorescently tagged antibodies (See Table S6) in FACS buffer (PBS, 1%FCS, 2mM EDTA, 0.02% sodium azide). All procedures are detailed in the Methods section of the paper.
Instrument	BD LSR II, FACSAria III and Cytek Aurora
Software	FlowJo v9 or v10 software (TreeStar)
Cell population abundance	For adoptive transfer experiments, memory WT or IL-2ra mut/mut OT-I cells were sorted by FACS. In N4-primed mice, the CD8+ compartment contained about 2-3% memory OT-I cells while T4-primed contained about 0.4%.

Gating strategy

In all plots, cell populations from spleen or blood were gated based on FSC-A and SSC-A and doublet exclusion. Further gating on lineage markers (i.e. CD3, CD8, or CD11c) and fluorescent (i.e. Td Tomato) and congenic markers (i.e. CD45.1, CD45.2) was specified for each respective panel.

[x] Tick this box to confirm that a figure exemplifying the gating strategy is provided in the Supplementary Information.
