# Supplementary material for: T cell receptor and IL-2 signaling strength control memory CD8+ T cell functional fitness via chromatin remodeling
Source: Nat Commun. 2022 Apr 26;13:2240. doi: 10.1038/s41467-022-29718-2 (PMC9042912; doi:10.1038/s41467-022-29718-2)
Supplement: Supplementary file 2 — Peer Review File [file 41467_2022_29718_MOESM2_ESM.pdf]

nature portfolio

Peer Review File

T cell receptor and IL-2 signaling strength control memory CD8 + T cell functional fitness via chromatin remodeling

Open Access This file is licensed under a Creative Commons Attribution 4.0 International License, which permits use, sharing, adaptation, distribution and reproduction in any medium or format, as long as you give appropriate credit to the original author(s) and the source, provide a link to the Creative Commons license, and indicate if changes were made. In the cases where the authors are anonymous, such as is the case for the reports of anonymous peer reviewers, author attribution should be to 'Anonymous Referee' followed by a clear attribution to the source work. The images or other third party material in this file are included in the article's Creative Commons license, unless indicated otherwise in a credit line to the material. If material is not included in the article's Creative Commons license and your intended use is not permitted by statutory regulation or exceeds the permitted use, you will need to obtain permission directly from the copyright holder. To view a copy of this license, visit http://creativecommons.org/licenses/by/4.0/.

REVIEWER COMMENTS

Reviewer #1 (Remarks to the Author):

This manuscript explores the relative roles of the TCR and IL-2 signaling in the integration of activation signals that control CD8 memory T cell programming and fitness. They use a combination of OVA altered epitopes with varying affinities for OT-1 T cells, as well as endogenous responses to a number of previously defined epitopes recombinantly expressed in Listeria, to monitor the responses of T cells with normal or impaired IL-2 signaling. In this way the authors were able to modulate both TCR and IL-2 signal strength during the primary response to infection and test the recall response capability of the ensuing memory T cells. They showed that dependence on IL-2 for the formation of functional memory T cells was variable and epitope dependent, and suggest these differences were driven by variable TCR signal strength. They go on to perform comprehensive gene expression, chromatin and flow cytometric analysis to define the relative roles of combined TCR+IL-2 signaling in the programming of memory T cell function. Overall, I found the study quite fascinating, and it sheds substantial light on the complex interplay between TCR and cytokine signaling with regards to effector and memory T cell function. The experiments are well done, and for the most part I found the conclusions to be justified by the presented experiments. However, I have a few moderate concerns about data interpretation as enumerated below.

1. The authors intend to conclude that dependence on IL-2 varies based on TCR signal strength, relying largely on the OVA variant epitopes. The variable dependence of various polyclonal responses on IL-2 complicates this interpretation. For example, while they argue that the dependence on IL-2 for the gp33-specific response (both polyclonal and P14) can be explained by unstable binding to Class I, other studies have found the polyclonal response to this epitope to demonstrate a normal range of affinity as compared to other epitopes, and P14 are on the high end of that affinity range. It therefore seems likely that the reasons underlying variable epitope dependence on IL-2 are complex. For example, one possibility is that IL-2 expands the repertoire diversity of the memory pool even when functional differences are not immediately apparent. A more nuanced discussion of these issues could be helpful.

2. Related to the point above, Figure 3F is confusing. While gp34-43 binds to Kb, gp33-41 binds Db, and that tetramer is used to measure responses. What is the relevance of Kb binding? This should be clarified, or the panel removed.

3. Do the authors have information on the IL-2-dependence of the polyclonal response to the OVA variants, especially A8? If available, this could provide support for the idea that IL-2-dependence of specific epitopes is related to the stability of binding to MHC, as compared to other polyclonal responses.

4. The Kb blockade and DTR treatments in Figure 3 are presented without controls. It would be helpful to show that treatment starting at day 0 impairs response to confirm efficacy.

5. DT treatments and confirmation of cellular depletion are not described in the methods.

Reviewer #2 (Remarks to the Author):

Using unique OVA peptides with differing TCR signal strength in conjunction with an IL-2R mutant animal, Chin and colleagues identify deficits in the proliferative potential and cytokine production by CD8+ memory T cells that are dependent upon TCR signal strength and IL-2 receptor signaling. Furthermore, these changes were found to be correlated with differences in chromatin accessibility, with several of the newly accessible regions being dependent upon either TCR signaling, IL-2 signaling, or a combination of both. Emphasizing the relative importance of TCR signal strength to memory differentiation, the authors used several unique epitopes with varying

TCR strength and found weaker TCR stimulation led to an increased dependence on IL-2 signaling during primary infection for complete memory differentiation and that these differences in signal strength can have unique effects on the subsequent amount of Tem and Tcm. Lastly, the authors identify that it is not the duration of antigen exposure that controls the quality of memory differentiation, but the strength and amount of both TCR and IL-2 signaling during the initial first 48 hours of the priming event. While there are several interesting findings in this study that bear further development, in present form, many of the major conclusions are overreaching and unsubstantiated.

Major Concerns:

1. A finding central this study is the importance of initial priming event to subsequent long-term memory formation. The authors' data (and other data in the field) indicate that early TCR activation events control the quality of memory cell differentiation with effects on the types of memory cells generated. However, no attempts are made to resolve the potential role of IL-2 signaling specifically on TCR sensitivity prior to priming. Several lines of evidence in the literature indicate that IL-2 signaling can reduce the initial threshold for TCR signaling events, especially in CD8 T cells. In light of the presented data on the importance of the initial priming event, further clarity on exactly when these signals are relevant prior to and after activation would improve this work. Additionally, controlling for the type of memory cells (i.e., Tem and Tcm) subsequently transferred for rechallenge would provide valuable insight as to any cell type specific deficits.
2. As both IL-2R signaling and TCR strength modify memory differentiation and functional properties of the cells generated, it would be beneficial to the authors' arguments if evidence were provided as to how decreased TCR signaling during these priming events can directly lead to late stage functional differences. The cells were similarly activated and have at least reached the threshold for proliferation so how did the early activation signals diverge in the cellular programming?
3. As the majority of the identified deficits in proliferative potential are performed after multiple competitive proliferation events it is difficult to resolve exactly when the TCR and IL-2 signaling pathways are having their effects following activation vs reactivation. To help resolve this issue, it would strengthen the authors' arguments to vary when these cells are actually directly in competition. For instance, do memory cells differentiated with weaker TCR stimulation or less IL-2 signaling similarly show these deficits when put into a competitive environment exclusively upon re-exposure? Little has been done to ensure that the cells are fully differentiated similarly prior to rechallenge and/or if these effects are just intrinsic deficits since the overall cellular response upon subsequent exposure seems to mirror the results for the initial priming event.
4. The ATAC-seq data and analysis presented are inadequate to justify the conclusion that "Memory CD8+ T cell epigenetic differences translate into phenotypic differences" as this finding is computationally based upon one data type. While it is appreciated that it would be technically challenging to isolate sufficient cell numbers to perform whole-genome ChIP-seq, at a minimum, CHIP-pcr at multiple relevant target sites for histone modifications and transcription factors would be needed to substantiate this claim. Similarly, none of the identified changes specifically explain the observed deficits as they were primarily metabolic in nature in what appear to be quiescent cells based upon the RNA expression data.
5. Related to points 1 and 4, no clear attempt has been made to explain why there are gross differences in chromatin accessibility nor what the functional effects are of these changes. Since the RNA-seq analysis could not identify functional differences in transcription under the differing differentiation conditions at later timepoints it begs the question of the functional relevance of the identified ATAC-seq changes. Further experiments to highlight actual changes in gene expression and/or cellular function that correlate to the identified differences would substantially improve the relevance of these data. Additionally, some evidence of the necessity of these chromatin changes in CD8 memory cell function would significantly strengthen the authors argument. There were also minimal attempts to interpretatively place these data in the context of the biological system from which they were collected and how changes near the identified genes could lead to the identified functional deficits.
6. Unfortunately, much of the presented data does not represent particularly large advances in our understanding of T-cell memory differentiation. Recent evidence has identified changes in chromatin accessibility during initial priming events that are necessary for functional memory formation in CD4 and CD8 T cells (Scharer 2017, Phan 2017). These changes have similarly been associated with digital gene expression and gross metabolic changes following early TCR signaling events and have similarly been shown to subsequently impact memory differentiation (Pearce

2009, DiToro 2018). Although it is quite interesting to identify these late stage functional deficits correlated to quality of initial TCR activation and IL-2 signaling, several groups have similarly demonstrated dramatic effects on the quality of Tcm and Tem differentiation that were similarly associated with TCR strength in both CD4 and CD8 T cells (Smith-Garven 2010, Obar 2010, Gasper 2014). As the current study's results are primarily observational and seemingly predictable based on prior work, without somehow linking these phenomena the current findings do not appear to represent a significant advance in our understanding of the mechanisms for memory differentiation.

Minor Concerns:
1. The in vitro and in vivo data analyses in Figure 1 have several issues, including a lack of separation of genes that are increasing or decreasing in the data set for ontology enrichment. Instead it appears all differentially regulated genes were combined in all data sets, substantially hindering the interpretation. The authors themselves identify the conflicting results on cell cycle progression between the in vitro and in vivo data; however this issue is glossed over and ignored. Why the difference (or why the in vitro data were included if the findings were not relevant)?
2. The representative flow plots in Figure 6A do no match the bar graph immediately adjacent nor in the text of the results, creating significant confusion.
3. There needs to be some discussion on why a competitive environment between these 2 cell populations seems to be necessary to identify the observed effects. This experimental design is unique to the results depicted here and is different between several of the previous findings that have found minimal functional effect of TCR strength on memory cell differentiation (Zehn 2009). Additionally, this competition could dramatically affect the potential viability due to the amount of IL-2 signaling that the mutant animals could be exposed to during both priming and re-exposure.
4. In the 3rd paragrapgh of the discussion there appears to be an inference that decreasing TCR strength leads to enhanced memory cell formation. However, since primary challenge with a weaker TCR signaling peptide lead to dramatically less cells overall and poorer memory function how would this be possible?
5. The discussion in general is speculative and primarily correlative. There would likely be substantial improvement if the authors were able to more directly link their conclusions to their data.
6. There are numerous syntax and grammatical errors throughout the paper such that the readability is substantially affected dramatically weakening the authors conclusions.

Reviewer #3 (Remarks to the Author):

Shu Shien Chin et al. present a potentially interesting study on the role of the T cell receptor strength and Il-2 signaling during memory CD8+ T cell differentiation. The authors suggest that during priming, both TCR strength and Il-2 signal regulate naïve CD8+ T differentiation into functional memory cells. They examine both T cell transcriptomic and epigenetic programming at several loci, among which genes related to metabolic processes.

Although the concept of Il-2 modulation of TCR signal is not new, the authors use elegant immunization and adoptive T cell transfer models to elucidate how these signals impact T cell fate determination. However, the role of IL-2 and TCR signaling strength in memory CD8+ T cell responses have been widely analyzed before.

Main concerns:
1. In Fig 1 A, the authors indicate that OT-I cells that receive weak TCR signals in vitro enter cell cycle and expand earlier than OT-I cells receiving strong TCR signals. These results should be also reported in terms of total numbers of both alive and death T cells. The readout outcomes in OT-I cells primed with N4, T4 and A8 in vitro and in vivo seem inconsistent, the authors should provide more details about these discrepancies.

2. In Fig 1C, the RNA-seq pathway analysis reveals several processes related to cell cycle/proliferation and death, metabolism, adhesion, migration, interferon and effector functions… These analyses are purely descriptive. Are the up-regulated genes functionally related with the observed phenotypes? More detailed cell cycle gene analyses could be included in Fig 1C. Is it possible that the differences in cell cycle genes (Fig 1D) reflect differences in phases of cell cycle?

3. In Fig 2, the authors use Il2ramut/mut OT-I cells in adoptive transfer model, to investigate the requirement for IL-2-signals to form functional memory. This is a constitutive mutant, as a consequence it is difficult to distinguish between the impact of the lack of IL-2 signaling during T cell precursor differentiation, during priming, and during recall. The importance of IL-2 during priming for memory development has been analyzed by several groups before (some of which are cited by the authors).

4. In Figure 4, the authors use different models to investigate IL-2-dependent and -independent contributions to memory responses to natural epitopes. Based on the stabilization assay results (Fig 3F), it seems that the peptides with lower stability promote weaker responses (GP33-41). These results should be discussed, the conclusion of the paragraph should be modified accordingly.

5. In Figure 6, the ATAC-seq results show several differences in open chromatin loci among the different experimental conditions. The authors suggest that the are genes associated with OCRs induced by changes in TCR signals selectively targeted metabolic processes. Which metabolic process? How these processes affect the differentiation of memory T cell subsets? The mechanisms involved should be explored more precisely.

REVIEWER COMMENTS

Reviewer #1 (Remarks to the Author):

This manuscript explores the relative roles of the TCR and IL-2 signaling in the integration of activation signals that control CD8 memory T cell programming and fitness. They use a combination of OVA altered epitopes with varying affinities for OT-1 T cells, as well as endogenous responses to a number of previously defined epitopes recombinantly expressed in Listeria, to monitor the responses of T cells with normal or impaired IL-2 signaling. In this way the authors were able to modulate both TCR and IL-2 signal strength during the primary response to infection and test the recall response capability of the ensuing memory T cells. They showed that dependence on IL-2 for the formation of functional memory T cells was variable and epitope dependent, and suggest these differences were driven by variable TCR cytometric analysis to define the relative roles of combined TCR+IL-2 signaling in the programming of memory T cell function. Overall, I found the study quite fascinating, and it sheds substantial light on the complex interplay between TCR and cytokine signaling with regards to effector and memory T cell function. The experiments are well done, and for the most part I found the conclusions to be justified by the presented experiments.
We thank this reviewer for her/his positive assessment.

However, I have a few moderate concerns about data interpretation as enumerated below.

1. The authors intend to conclude that dependence on IL-2 varies based on TCR signal strength, relying largely on the OVA variant epitopes. The variable dependence of various polyclonal responses on IL-2 complicates this interpretation. For example, while they argue that the dependence on IL-2 for the gp33-specific response (both polyclonal and P14) can be explained by unstable binding to Class I, other studies have found the polyclonal response to this epitope to demonstrate a normal range of affinity as compared to other epitopes, and P14 are on the high end of that affinity range. It therefore seems likely that the reasons underlying variable epitope dependence on IL-2 are complex. For example, one possibility is that IL-2 expands the repertoire diversity of the memory pool even when functional differences are not immediately apparent. A more nuanced discussion of these issues could be helpful.
It is indeed possible that the mechanisms accounting for our findings in the TCR transgenic systems (OT-I, P14) and the corresponding polyclonal responses are different. We have included this discussion point in the revised paper.

2. Related to the point above, Figure 3F is confusing. While gp34-43 binds to Kb, gp33-41 binds Db, and that tetramer is used to measure responses. What is the relevance of Kb binding? This should be clarified, or the panel removed.
We provide new data that measure D^b stability after binding by the natural GP_{33-41} peptide, the GP_{33M41} stabilized variant where C41 is replaced by M41, and the $GP_{276-286}$ peptide (**Figure 4F**). The data shows very little capacity of the natural GP_{33-41} and $GP_{276-286}$ peptides to make stable complexes with D^b, consistent with our proposed model.
With regards to the relevance of K^b binding, earlier work (Hudrisier et al., Virology. 1997;234(1):62-73) had reported that the LCMV-derived GP_{33-41} peptide can be bound by both D^b and K^b, which was the rationale behind measuring its capacity to stabilize K^b. We agree that this result adds only confusion to the paper, therefore we have removed it from the revised version.

3. Do the authors have information on the IL-2-dependence of the polyclonal response to the OVA variants, especially A8? If available, this could provide support for the idea that IL-2-

dependence of specific epitopes is related to the stability of binding to MHC, as compared to other polyclonal responses.

This is a technically challenging experiment to design since *Lm*-A8 immunization induces over ~20 times less OT-I cells than *Lm*-N4 at memory stage (**See Figure A.A below**). We attempted an experiment without transferring OT-I cells to track polyclonal memory CD8$^+$ T cells induced upon *Lm*-A8 immunization, but we were unable to detect any N4-tetramer$^+$ CD8$^+$ T cells in mice immunized with *Lm*-A8 (**Figure A.B below**). While these experiments were not technically feasible, we conducted several additional experiments to get to this reviewer's point (see below).

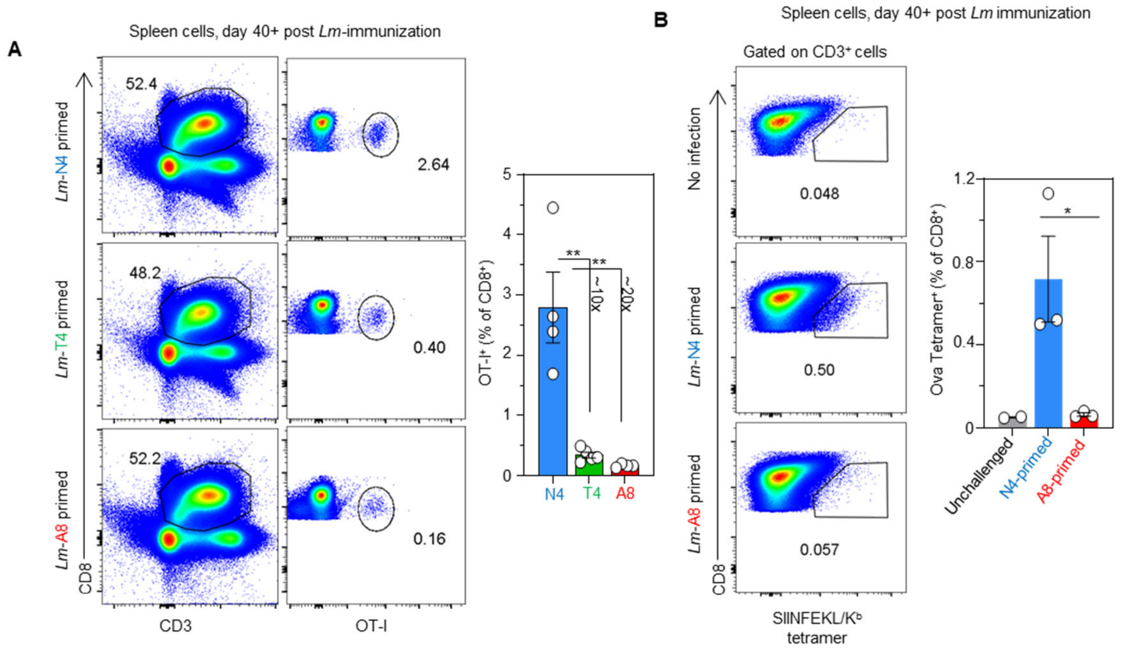

Figure A: (A) Mice transferred with 2,500 Td+ OT-I T cells i.v. were immunized the next day with indicated *Lm* and 40 days later spleen harvested and stained with Abs against cell surface CD3 and CD8. Frequency of OT-I memory cells among CD8$^+$ T cells is shown in a representative mouse and bar graph show data summary across n=3 mice in 1 of >15 experiments. **(B)** Same experimental set up as in A) except that mice did not receive OT-I cells. At day 40 post immunization, spleen cells were harvested and stained for cell-surface CD3, CD8 and SIINFEKL tetramers. A representative mouse dot plot is shown with a bar graph summarizing results across n=3 mice.

Before we describe them, we would like to clarify our interpretation of the results. We believe that the data using various SIINFEKL APLs indicate that the key parameter is the amount of TCR signals that are received/integrated by naïve CD8$^+$ T cells at the time of priming. This can be achieved either by lowering TCR and peptide interactions (modeled by the T4 or Q4 APLs) or by disrupting the overall peptide/MHC stability, which subsequently disrupts TCR interactions (modeled by the A8 APL). Thus, as an alternative to further support our interpretation that peptide/MHC stability is a key parameter in determining IL-2 dependency, we have conducted a new experiment which design is based on that of **Figure 4A**. Briefly, we transferred OT-I cells at day 1, 3 or 5 post-immunization with *Lm*-A8, *Lm*-T4 or control *Lm*-N4, to monitor how long each Ova APL is presented *in vivo* (**revised Figure 4A**). While N4 is presented more than 5 days, A8 presentation is lost very quickly between 1 and 3 days post challenge and T4 is presented until day 3. This result indicates that the A8 APL is only "seen" by the OT-I T cells for a very short amount of time *in vivo*. Together with the MHC-I stabilization assay, these data support the idea

that epitopes that cannot form stable complexes with MHC-I are less likely to provide robust TCR signals to naïve T cells.

4. The Kb blockade and DTR treatments in Figure 3 are presented without controls. It would be helpful to show that treatment starting at day 0 impairs response to confirm efficacy.
We apologize for not providing these controls in the initial version of our manuscript. These data have now been included in a **new Figure S4** in the revised paper. Panel A) shows that anti-K^b/SIINFKEL Ab blockade during priming decreases the size of OT-I T cell expansion by ~40% compared to control isotype Ab-treated mice. The treatment impacts WT and *Il2ra*$^{mut/mut}$ OT-I cells similarly. Panels B) and C) are related to the *CD11c*^{DTR} experiments: B) shows that depletion of $CD11c^+$ cells 2 days after *Lm*-Ova infection, decreases OT-I cell expansion by ~50%. Both WT and *Il2ra*$^{mut/mut}$ OT-I cells are similarly impacted by the treatment. Panel C) further shows that when DT-treatment occurs prior to the priming, OT-I cells failed to be primed and expand, as expected. The selective elimination of $CD11c^{hi}$ cells upon DT injection is also provided in the dot plots below. Altogether, this set of data shows that both types of treatments led to impaired primary OT-I cell expansion (decrease by ~40-50%).

5. DT treatments and confirmation of cellular depletion are not described in the methods.
We thank this reviewer for her/his comment and have now added this information to the methods of the revised paper.

Reviewer #2 (Remarks to the Author):

Using unique OVA peptides with differing TCR signal strength in conjunction with an IL-2R mutant animal, Chin and colleagues identify deficits in the proliferative potential and cytokine production by CD8+ memory T cells that are dependent upon TCR signal strength and IL-2 receptor signaling. Furthermore, these changes were found to be correlated with differences in chromatin accessibility, with several of the newly accessible regions being dependent upon either TCR signaling, IL-2 signaling, or a combination of both. Emphasizing the relative importance of TCR signal strength to memory differentiation, the authors used several unique epitopes with varying TCR strength and found weaker TCR stimulation led to an increased dependence on IL-2 signaling during primary infection for complete memory differentiation and that these differences in signal strength can have unique effects on the subsequent amount of Tem and Tcm. Lastly, the authors identify that it is not the duration of antigen exposure that controls the quality of memory differentiation, but the strength and amount of both TCR and IL-2 signaling during the initial first 48 hours of the priming event. While there are several interesting findings in this study that bear further development, in present form, many of the major conclusions are overreaching and unsubstantiated.
We have conducted multiple additional experiments over the past 7 months to address the major critiques raised by this reviewer. We hope that these additional data will now convince her/him.

Major Concerns:
1. A finding central this study is the importance of initial priming event to subsequent long-term memory formation. The authors' data (and other data in the field) indicate that early TCR activation events control the quality of memory cell differentiation with effects on the types of memory cells generated. However, no attempts are made to resolve the potential role of IL-2 signaling specifically on TCR sensitivity prior to priming. Several lines of evidence in the literature indicate that IL-2 signaling can reduce the initial threshold for TCR signaling events, especially in CD8 T cells. In light of the presented data on the importance of the initial priming

event, further clarity on exactly when these signals are relevant prior to and after activation would improve this work.

We believe the main work this reviewer refers to shows that IL-2 signals lowers TCR signaling threshold during (but not prior to) priming (J. Immunol 2017, 198:2445-56). This work, and related literature (from the Cantrell group in particular), focused on the impact of IL-2 signaling on TCR signaling events and subsequent primary $CD8^+$ T cell expansion, mostly *in vitro*. Data from these studies show that IL-2 signaling lowers the threshold of TCR signaling needed to achieve full TCR-mediated activation and primary expansion. While these prior observations are certainly consistent with our findings, none of these studies investigated if varying these signals impacted the chromatin accessibility (thus the epigenetic programming) and the functional features of induced memory cells. Our data provide a global view of how the modulation of these signals changes memory $CD8^+$ T cell chromatin landscape and multiple functional features. Also, we reveal that IL-2 signals do not simply rescue low TCR signals to form fully functional memory cells, but rather that these signals act synergistically to control a whole new chromatin landscape that is distinct from those controlled by either signal alone.

As far as the timing of these signals for optimal primary T cell activation and expansion, the prior cited studies did not resolve when they may be required. We have performed additional experiments and provide additional data in which we have provided IL-2 signals with IL-2/anti-IL-2 mAb complexes treatment for 3 days instead of 6, when OT-I T cells are primed with the weak agonist T4 (**revised Figure 2C**). The results show that the extent of memory cell rescue is comparable under both treatment regimen, suggesting that IL-2 signals are mostly needed early on during priming (See also our response to point 3 from Reviewer #3).

Additionally, controlling for the type of memory cells (i.e., Tem and Tcm) subsequently transferred for rechallenge would provide valuable insight as to any cell type specific deficits.

We attempted to conduct this experiment, however we were not able to purify enough *Il2ra*$^{mut/mut}$ OT-I TCM and TEM cells induced by *Lm*-T4 immunization to reliably perform the adoptive transfer experiment of OT-I memory cell subsets in multiple mice (**Figure B.A below**). Priming with *Lm*-T4 induces >10 times fewer memory cells than with *Lm*-N4, and the *Il2ra*$^{mut/mut}$ represent ~only 25% of them, and this needs further subdivision into TEM and TCM subsets (**Figure B.B**). We nevertheless provide as a communication to this reviewer, the outcome of sorting and transferring WT OT-I TCM and TEM subsets, or control bulk WT OT-I memory cells induced upon *Lm*-N4 or *Lm*-T4 immunizations to naïve recipient mice challenged the next day (**Figure B.C**). WT OT-I TCM largely outcompeted TEM cells, as also expected from the literature. This result indicated that, in this experimental setting, the TCM compartment accounts for the differences in clonal expansion competitiveness between WT OT-I memory cells primed with strong compared to weak peptide agonists.

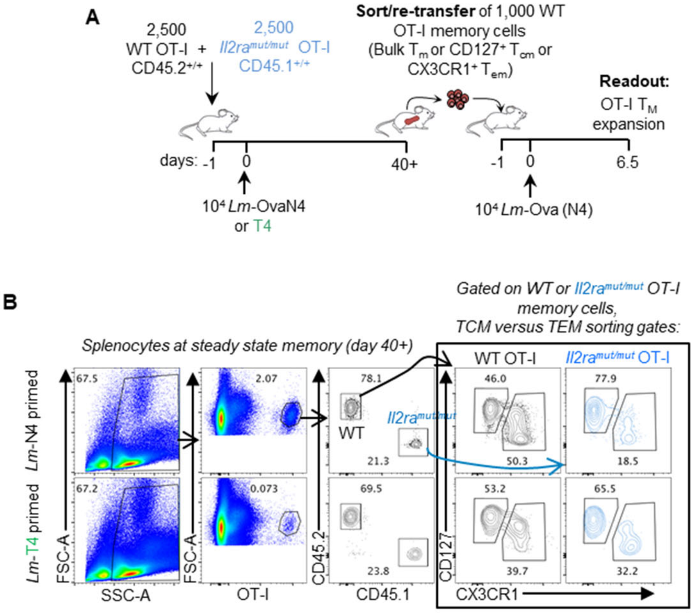

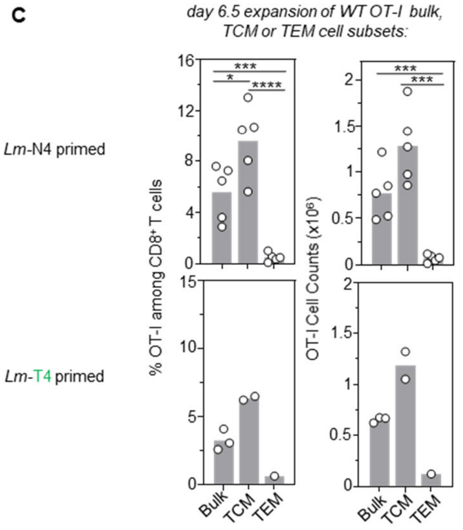

Figure B: (**A**) Schematic of experimental design. (**B**) Spleen cells from day 40-immunized mice were stained for CD45 congenic markers and CD127, CX3CR1. Sorting gates are shown for OT-I T_M cells (Tomato+), CD45 congenic markers and CX3CR1/CD127 with respective frequencies. 1,000 bulk, TCM or TEM OT-I memory cells were sorted in the WT OT-I compartment induced upon either *Lm*-N4 or *Lm*-T4 immunizations. (**C**) Transferred WT OT-I T_M cell (bulk, TCM and TEM) expansion 6.5 days post recall infection across n=5 mice.

2. As both IL-2R signaling and TCR strength modify memory differentiation and functional properties of the cells generated, it would be beneficial to the authors' arguments if evidence were provided as to how decreased TCR signaling during these priming events can directly lead to late stage functional differences. The cells were similarly activated and have at least reached the threshold for proliferation so how did the early activation signals diverge in the cellular programming?

This is a very interesting question that we are planning to investigate in the upcoming years. It is very likely that epigenetic regulators are involved in setting up the chromatin accessibility but we currently do not have evidence for this. We have included a short discussion point on this in the revised manuscript. The current study represents 7+ years of work already and the question raised by this reviewer seems beyond its scope.

3. As the majority of the identified deficits in proliferative potential are performed after multiple

competitive proliferation events it is difficult to resolve exactly when the TCR and IL-2 signaling pathways are having their effects following activation vs reactivation. To help resolve this issue, it would strengthen the authors' arguments to vary when these cells are actually directly in competition. For instance, do memory cells differentiated with weaker TCR stimulation or less IL-2 signaling similarly show these deficits when put into a competitive environment exclusively upon re-exposure?

This is a good point. To get to this question, we generated WT or *Il2ra*$^{mut/mut}$ OT-I memory cells in a non-competitive setting. We also produced WT or *Il2ra*$^{mut/mut}$ OT-I memory cells in a competitive setting as control. At memory stage (day 40+ post immunization), we FACS-sorted OT-I memory cells and co-transferred them to recipient mice that we subsequently challenged with *Lm*-N4 the next day (as depicted in the schematic, **Figure C.A below**). Both *Lm*-T4- and *Lm*-A8-primed *Il2ra*$^{mut/mut}$ OT-I memory cells expanded less competitively than WT counterpart whether induced in a competitive setting or not, confirming our original findings.

However, *Il2ra*$^{mut/mut}$ OT-I memory cells induced by *Lm*-N4 immunization in a non-competitive setting exhibited impaired expansion compared to that of WT (**Figure C.B below**). This unexpected result shows that in an experimental setting where a higher than physiological number of OT-I cells (2,500 cells) are transferred and primed with a high affinity epitope (N4), WT OT-I cells acquire better proliferative potential than *Il2ra*$^{mut/mut}$ counterparts at memory stage. This led us to hypothesize that in a non-competitive setting, such high number of WT OT-I cells outcompetes any endogenous polyclonal CD8^{+} T cells during priming, presumably integrating more priming signal and achieving better differentiation. Thus, we next conducted the same experiment but by transferring physiological numbers of OT-I cells (i.e., 100 cells) either in a competitive or a non-competitive setting (**Figure C.C below**). As postulated, when low, physiological numbers of OT-I cells were used, *Il2ra*$^{mut/mut}$ OT-I memory cells remained as competitive as WT counterparts in either situation. Therefore, generating OT-I memory cells with higher than physiological numbers of naïve OT-I cells in a competitive setting is in fact closer to the normal physiology than in a non-competitive setting. In summary, these results validate our original conclusion that naïve CD8^{+} T cells that receive lower TCR and IL-2 signals fail to acquire full proliferative potential at memory stage. For clarity and brevity of the revised manuscript, and since the major conclusion remains, we have decided to provide these results as a private communication to the reviewers.

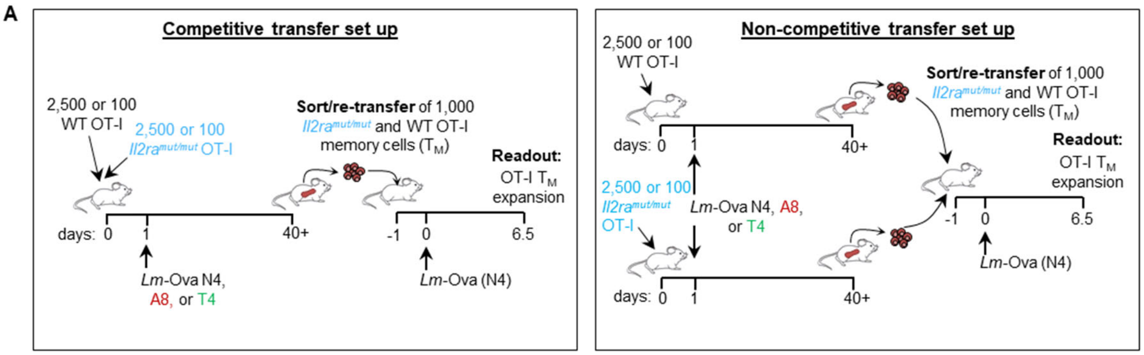

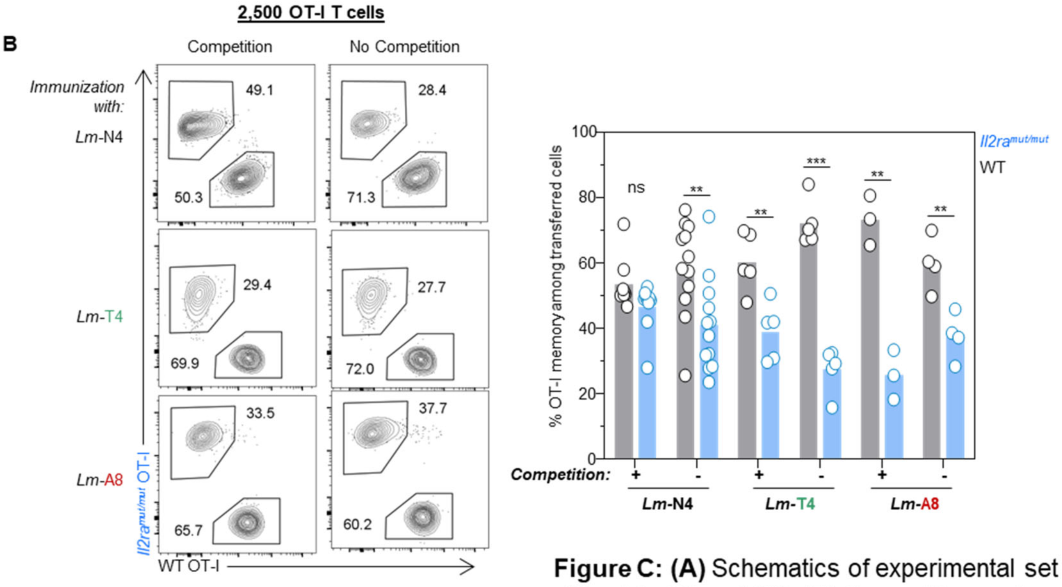

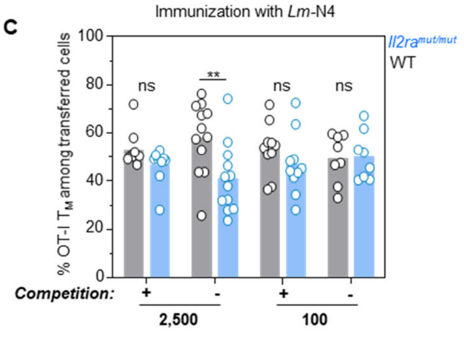

Figure C: (A) Schematics of experimental set ups utilized in (B) and (C). **(B)** Competitive and non-competitive adoptive transfers of 2,500 congenically distinct WT or *Il2ra*$^{mut/mut}$ OT-I cells in mice immunized with 10^4 *Lm*-N4, *Lm*-T4 or *Lm*-A8. 40+ days after immunization, 1,000 OT-I memory cells of each genotype were FACS sorted and co-transferred to recipient mice that were challenged with 10^4 *Lm*-N4 the next day. 6.5 days later, OT-I memory cell expansion was quantified by FACS in the spleens of recipient mice. (C) The same experiment as above was conducted but using adoptive transfers of either 100 or 2,500 WT or *Il2ra*$^{mut/mut}$ OT-I cells subsequently immunized with *Lm*-N4. Expansion of 1,000 purified and re-transferred OT-I memory cells was quantified as in (B). Experiments were repeated independently at least twice (n=8-10 mice). Representative FACS dot plots are shown with summary bar graphs. Each symbol corresponds to 1 individual mouse and p-values are indicated.

Little has been done to ensure that the cells are fully differentiated similarly prior to rechallenge and/or if these effects are just intrinsic deficits since the overall cellular response upon subsequent exposure seems to mirror the results for the initial priming event.

We are not sure we understand this reviewer's point. Extensive immunophenotyping (25+ markers) of OT-I memory cells induced under the various priming conditions reveals clear

differences in multiple markers expression in steady state memory cells, suggesting differences in the differentiation fate of these cells. Importantly, in the case of *Lm*-N4-primed OT-I memory cells (both WT and *Il2ra*$^{mut/mut}$), WT OT-I cells outcompete *Il2ra*$^{mut/mut}$ counterparts during the primary response (**Figure S2A**). However, both WT and *Il2ra*$^{mut/mut}$ OT-I memory cells expanded comparably during secondary Ag encounter (**Figure 2B**). This was not the case for weak agonists (Q4, T4, A8) where *Il2ra*$^{mut/mut}$ OT-I memory cells remained outcompeted by WT counterparts, further indicating impaired functional differentiation of these cells.

4. The ATAC-seq data and analysis presented are inadequate to justify the conclusion that "Memory CD8+ T cell epigenetic differences translate into phenotypic differences" as this finding is computationally based upon one data type. While it is appreciated that it would be technically challenging to isolate sufficient cell numbers to perform whole-genome ChIP-seq, at a minimum, CHIP-pcr at multiple relevant target sites for histone modifications and transcription factors would be needed to substantiate this claim.
We agree that this sub-heading was overstated. We have modified it in the revised version so that it reflects the data shown in **Figures 8 and S8**. We have also carefully modified this section to avoid overstatements.
Similarly, none of the identified changes specifically explain the observed deficits as they were primarily metabolic in nature in what appear to be quiescent cells based upon the RNA expression data.
Figures 9 and S9 were meant to get to this exact question. Our rationale was that if chromatin regions were differentially accessible, that would potentially translate into functional difference upon reactivation of the memory cells. However, and as pointed out by this reviewer, more direct evidence that specific OCRs and related genes translate into functional differences of expression was lacking.

With this comment in mind, we delved back into our data, the GO pathway analyses and the actual genes. From the non-filtered list of GO pathways associated with genes with unique OCRs when TCR, IL-2 or TCR+IL-2 signals vary during priming (**Table S4**), we generated a new REViGO plot that is more representative of the differential/common pathways (**New Figure 6D**). This notably revealed that when both signals are modulated together, the OT-I memory cells acquire a much broader chromatin accessibility than when only TCR signals were changed. This reanalysis of the GO pathway data also shows that the large majority of the pathways modulated by TCR signal changes were also modulated by TCR+IL-2 signal changes. Few select GO pathways related to specific metabolic processes (Table S4) were unique to TCR signal changes but it was difficult to link them to functional read-outs (See also our answer to point 5 of Reviewer #3).

To more specifically address this reviewer's comment, we focused on a set of newly highlighted GO pathways, related to stem cells, calcium signaling and cell cycling which represent important hallmarks of T cell functional fates and activation. Our rationale to focus on such differentially accessible OCRs was that these were likely to be essential in the rapid and differential modulation of memory OT-I cell activation and clonal expansion. Thus we analyzed genes related to these GO pathways and whether they presented differentially accessible OCRs in the promoter area (+/-3kb), close to the start site of transcription (TSS)(**new Figure 7 and S7**). **Figure 7A** reports the state of these differentially accessible OCRs in several genes (yellow:open, grey:closed) in the different pathways in WT or *Il2ra*$^{mut/mut}$ OT-I memory cells primed by either *Lm*-N4 or *Lm*-T4. In **Figure 7B and Table S5**, we next focused of some of these key genes (*Tcf7l2, Dvl2, Cdc16, Cacna1c*) and searched for TFs known to bind to these differentially accessible OCRs. The data reveals that most of these OCRs have predicted binding sites for many TFs that belong to distinct family of TFs. This suggests that these OCRs may represent important areas of regulation for the expression each of these genes.

Next, we used i) high dimensional spectral flow cytometry to provide a deeper phenotypic assessment of resting memory cells induced under the various conditions (**Figures 8 and S8**), and ii) functional FACS assays to directly monitor cell cycle stages and intracellular calcium accumulation at early (16 hrs) and late (day 4, 6, 9 and 12) stages post-challenge infections (**new Figures 9 and S9**).

Using flow cell organizing map (FlowSOM) representation, results in **Figures 8 and S8** show higher subset proportions and phenotypic diversity in memory cells primed under strong TCR and IL-2 signals. In particular, we note markers associated with a stem cell-memory-like phenotype (TCF-1, Eomes, Bcl-2, Sca-1, CD122, CD127, CD27) with significantly higher expression in the subsets included in the broad category of T_{CM} cells. Results in **Figures 9 and S9** show that early on post-reactivation, a significantly greater proportion of T4-induced *Il2ra*$^{mut/mut}$ OT-I memory cells (~6 times more) remained in G_0 phase while N4-counterparts had already transitioned to G_1 (**Figure 9C**). As a control comparison, a much higher proportion of naïve T cells were in G_0 compared to early activated N4-memory or effector (day 5) cells (up to ~20 times). In addition to this faster initiation of cell cycle of N4-induced WT OT-I memory cells, we also observed a more robust and competitive later expansion of these cells than that of both WT and *Il2ra*$^{mut/mut}$ primed OT-I memory cells with the weak T4 agonist (**Figure 9D**). Thus, rapid memory cell entry into cell cycle and competitive proliferation/expansion largely depended on the combination of robust TCR and IL-2 signals during priming, consistent with our deeper analyses of the differentially accessible OCRs in the promoters of stem-cell and cell-cycle related genes (**Figure 7**). In addition, intracellular calcium accumulation was significantly higher in N4-primed OT-I memory cells compared to T4-primed counterparts, whether on the WT or *Il2ra*$^{mut/mut}$ background, suggesting calcium-dependent processes in the memory cells largely depended on robust TCR signals during priming (**Figure 9B**). This was despite many relevant OCRs being differentially accessible when both TCR and IL-2 signals varied (**Figure 7A**). However, we also noted that *Cabp1* (Calcium binding protein 1) and *Camk1* (Calcium/calmodulin-dependent protein kinase) accessible OCRs were only modulated by TCR signaling strength, suggesting these genes could potentially act as key regulators of intracellular calcium fluxes in early reactivated memory $CD8^+$ T cells.

In summary, this new set of data establishes a stronger correlation between chromatin accessibility at promoter area of genes related to stemness, cell cycle and calcium fluxes, and the phenotypic subset diversity/proportions in resting memory cells and the measurable proliferative and intracellular calcium responses of reactivated memory cells that were induced with strong or weak TCR and IL-2 signals.

5. Related to points 1 and 4, no clear attempt has been made to explain why there are gross differences in chromatin accessibility nor what the functional effects are of these changes. Since the RNA-seq analysis could not identify functional differences in transcription under the differing differentiation conditions at later timepoints it begs the question of the functional relevance of the identified ATAC-seq changes. Further experiments to highlight actual changes in gene expression and/or cellular function that correlate to the identified differences would substantially improve the relevance of these data.
See our response to point #4 above.
Additionally, some evidence of the necessity of these chromatin changes in CD8 memory cell function would significantly strengthen the authors argument. There were also minimal attempts to interpretatively place these data in the context of the biological system from which they were collected and how changes near the identified genes could lead to the identified functional deficits.
To prove that some of the specific OCRs highlighted above (or even others) are involved in corresponding gene expression and memory T cell functions, inducible genetic deletion of such OCRs in the T cells at memory stage would be necessary. This requires the development of

either conditional knock-in or CRISPR-mediated deletion mouse models which seems the very logical next step of our studies but requires many more years to complete.

6. Unfortunately, much of the presented data does not represent particularly large advances in our understanding of T-cell memory differentiation. Recent evidence has identified changes in chromatin accessibility during initial priming events that are necessary for functional memory formation in CD4 and CD8 T cells (Scharer 2017, Phan 2017). These changes have similarly been associated with digital gene expression and gross metabolic changes following early TCR signaling events and have similarly been shown to subsequently impact memory differentiation (Pearce 2009, DiToro 2018). Although it is quite interesting to identify these late stage functional deficits correlated to quality of initial TCR activation and IL-2 signaling, several groups have similarly demonstrated dramatic effects on the quality of Tcm and Tem differentiation that were similarly associated with TCR strength in both CD4 and CD8 T cells (Smith-Garven 2010, Obar 2010, Gasper 2014).
The cited studies as well as many others have indeed established that after priming, naïve T cells undergo robust epigenetic changes as they differentiate into effector and memory cells. Chromatin accessibility changes have also been associated with many well-known improved functional features of memory cells, including essential metabolic changes. In addition, the fact that stronger TCR signaling strength favors the onset of terminally differentiated effector cells is well known. What has not been shown, however, is that TCR and IL-2 signaling strengths control specific chromatin accessibility patterns and can modulate a significant proportion of the genome. To our knowledge, none of the previously cited studies, or even others in the current literature, have made such link and defined specific OCRs modulated by these signals as we did. These results provide a first key step that needs to be reported to the broader scientific community, so that further investigations of the underlying molecular mechanisms orchestrating such chromatin accessibility, can be undertaken. We believe these findings set the stage for a better understanding of memory $CD8^+$ T cell epigenetic programming controlled by TCR signaling strength, in the presence or the absence of robust IL-2 signals.
As the current study's results are primarily observational and seemingly predictable based on prior work, without somehow linking these phenomena the current findings do not appear to represent a significant advance in our understanding of the mechanisms for memory differentiation.
As reported in response to point 4, we have conducted additional experiments to connect specific OCRs and the acquisition of functional features by the memory cells (**Figures 7, 8, 9 and S7, S8, S9**). While it is not feasible to correlate all OCR/gene differences we report with functional read-outs, we believe the new data added to the revised manuscript provide a closer "proof of concept" of the validity of our findings.

Minor Concerns:
1. The in vitro and in vivo data analyses in Figure 1 have several issues, including a lack of separation of genes that are increasing or decreasing in the data set for ontology enrichment. Instead it appears all differentially regulated genes were combined in all data sets, substantially hindering the interpretation.
We thank this reviewer for her/his suggestion. We have re-run the GO pathway analysis on the differentially expressed genes that were up- or down-regulated (**new Figure 1C and Table S1**). The data clearly highlight that *Lm*-T4- and *Lm*-A8-primed OT-I T cells exhibit a very similar expression program in upregulated genes reflected in the overlapping immune pathways compared to the *Lm*-N4-primed T cells. The differences in the pathways related to the differentially down-regulated genes were mostly related to non-immune functions. Essentially no pathways were found between *Lm*-A8- and *Lm*-T4-primed OT-I cells, consistent with the very small number of differentially expressed genes between these two priming conditions.

The authors themselves identify the conflicting results on cell cycle progression between the in vitro and in vivo data; however this issue is glossed over and ignored. Why the difference (or why the in vitro data were included if the findings were not relevant)?
Several cell-cycle cyclin-dependent kinase-encoding genes (*cdkn3, cdkn2d, cdkn2c, cables1*) and genes coding for the E2F2 transcription factor and Trp53inp1, are upregulated in OT-I cells primed with weak (T4, A8) compared to the strong peptide agonist (N4). E2F2 and Trp53inp1 suppress cell cycle and the various kinases listed before are reported to act as negative regulators of cell cycle progression. These observations are therefore consistent with the finding that T4- and A8-primed OT-I cells underwent less sustained proliferation and arrest cell cycle faster than N4-primed counterparts, both *in vitro* and *in vivo*. Also, *cdk6* and *myc* genes are upregulated in N4-primed OT-I cells, and both are reported to drive robust cell cycle progression and proliferation, consistent with N4-primed OT-I cell undergoing prolonged proliferative response than T4/A8-primed counterparts. We have now clarified these points in the corresponding revised result section.

2. The representative flow plots in Figure 6A do no match the bar graph immediately adjacent nor in the text of the results, creating significant confusion.
This has now been corrected. We apologize for the confusion.

3. There needs to be some discussion on why a competitive environment between these 2 cell populations seems to be necessary to identify the observed effects. This experimental design is unique to the results depicted here and is different between several of the previous findings that have found minimal functional effect of TCR strength on memory cell differentiation (Zehn 2009).
We do not know if transferring the distinct OT-I memory cells into different hosts would phenocopy the results obtained in the competitive transfer setting, e.g., if T4-primed WT OT-I memory cells would expand better than *Il2ra*$^{mut/mut}$ counterparts in different hosts. However, the only rigorous approach to compare the proliferative potential of T cells and obtain accurate cell numbers is to use adoptive transfers of the exact same numbers of memory cells. The Zehn experiments compared the frequencies of OT-I memory cells primed by the various Ova APLs among CD8^{+} T cells at peak recall response in the blood (day 4). These data showed that OT-I memory cells expanded comparably whichever the priming Ova APL was, a result that is consistent with ours, when we compared *Lm*-N4 and *Lm*-T4-primed OT-I memory cells (See **Figure 9D**). In this paper, expansion of OT-I memory cells primed with weaker Ova APLs and impaired in IL-2 signaling was never tested. With regards to memory cell differentiation/fate, the only activation marker that was monitored was CD44. We provide a high dimensional FACS analysis with 20+ discriminative markers which reveals many differences that could not be detected in this prior study.
Additionally, this competition could dramatically affect the potential viability due to the amount of IL-2 signaling that the mutant animals could be exposed to during both priming and re-exposure.
We are not sure we understand this reviewer's point. Only WT mice were used as recipients of WT and *Il2ra*$^{mut/mut}$ OT-I cells in all experiments.

4. In the 3rd paragrapgh of the discussion there appears to be an inference that decreasing TCR strength leads to enhanced memory cell formation. However, since primary challenge with a weaker TCR signaling peptide lead to dramatically less cells overall and poorer memory function how would this be possible?
We refer, in this section of the discussion, to the functional characteristics of the memory cells that were generated, not their numbers. In particular, prior **Figure 8B** showed that early on after challenge infection, OT-I memory cells induced upon weak APL priming and low IL-2 signals were Tbet^{hi} and expressed higher effector functions (IFNγ, CCL3). However, their numbers were

significantly lower (up to 10 times, **Figure A.A,** See also response to reviewer #1, point 3), and these cells were also impaired in their ability to re-expand (**new Figure 9D**). Thus, while the memory cells induced by weak peptide agonists express higher levels of some effector molecules early post activation, these cells are clearly outnumbered by those induced with strong agonists, and they are less competitive during expansion. In that sense, this reviewer's point is well taken, and we have clarified our thinking accordingly in the revised discussion.

5. The discussion in general is speculative and primarily correlative. There would likely be substantial improvement if the authors were able to more directly link their conclusions to their data.
The discussion has been substantially modified in light of the new results.

6. There are numerous syntax and grammatical errors throughout the paper such that the readability is substantially affected dramatically weakening the authors conclusions.
We apologize for these errors. We believe that the revised manuscript has been now carefully edited to prevent such issue.

Reviewer #3 (Remarks to the Author):

Shu Shien Chin et al. present a potentially interesting study on the role of the T cell receptor strength and Il-2 signaling during memory CD8+ T cell differentiation. The authors suggest that during priming, both TCR strength and Il-2 signal regulate naïve CD8+ T differentiation into functional memory cells. They examine both T cell transcriptomic and epigenetic programming at several loci, among which genes related to metabolic processes.

Although the concept of Il-2 modulation of TCR signal is not new, the authors use elegant immunization and adoptive T cell transfer models to elucidate how these signals impact T cell fate determination. However, the role of IL-2 and TCR signaling strength in memory CD8+ T cell responses have been widely analyzed before.
We thank this reviewer for the positive comments. With regards to the role of IL-2 and TCR signaling strength, the major take home message from current literature seems that strong TCR and IL-2 signals favor a terminal effector state and robust T cell expansion while the opposite rather promotes a memory fate. To our knowledge, however, no studies i) have shown that the strength of TCR signaling determined the requirement for strong IL-2 signaling to form memory cells that can expand competitively, ii) have linked the strength of these signals to specific sets of OCRs and related genes/pathways in the memory cells, iii) have correlated the chromatin accessibility pattern in specific genes of resting memory $CD8^+$ T cells to functional read-outs after reactivation.

Main concerns:
1. In Fig 1 A, the authors indicate that OT-I cells that receive weak TCR signals in vitro enter cell cycle and expand earlier than OT-I cells receiving strong TCR signals. These results should be also reported in terms of total numbers of both alive and death T cells.
These experiments did not include a live/dead stain, however, careful FSC/SSC gating was performed to mostly exclude dead/dying cells. These are *in vitro* priming assays with peptide-pulsed splenocytes that prime robust T cell responses and activated T cells quickly acquire notable phenotypic changes that are easily measurable in FSC and SSC. Apoptotic and dead cells exhibit very different FSC/SSC compared to activated, proliferating T cells and therefore can be largely excluded. In order to streamline the message of the paper, we now provide two different measures that more directly reflect OT-I cell proliferative response after N4, T4 or A8

peptide stimulation (**Figure 1A, top graphs**). The proliferation index (top left) is the average generation number among cells that have divided at least once and the fraction diluted is the proportion of all cells that have divided at least once. The data show that i) N4 induces more prolonged OT-I cell proliferation than the weaker agonists T4 or A8 and ii) T4 and A8 exhibit a closely comparable proliferative response.

The readout outcomes in OT-I cells primed with N4, T4 and A8 in vitro and in vivo seem inconsistent, the authors should provide more details about these discrepancies.

We have provided a detailed response to this comment also raised by Reviewer #2 (minor point 1). We also paste our answer below:

Several cell-cycle cyclin-dependent kinase-encoding genes (*cdkn3, cdkn2d, cdkn2c, cables1*) and genes coding for the E2F2 transcription factor and Trp53inp1, are upregulated in OT-I cells primed with weak (T4, A8) compared to strong peptide agonists. E2F2 and Trp53inp1 suppress cell cycle and the various kinases listed before are reported to act as negative regulators of cell cycle progression. This is therefore consistent with the finding that T4- and A8-primed OT-I cells underwent a lower average generation number (division index 0.3-0.5) and arrest proliferation faster than N4-primed counterparts, both *in vitro* and *in vivo*. Also, *cdk6* and *myc* genes are upregulated in N4-primed OT-I cells, and both are reported to drive robust cell cycle progression and proliferation, consistent with N4-primed OT-I cell undergoing stronger proliferative response than T4/A8-primed counterparts (division index 0.6). We have now clarified these points in the corresponding revised result section.

2. In Fig 1C, the RNA-seq pathway analysis reveals several processes related to cell cycle/proliferation and death, metabolism, adhesion, migration, interferon and effector functions… These analyses are purely descriptive. Are the up-regulated genes functionally related with the observed phenotypes? More detailed cell cycle gene analyses could be included in Fig 1C. Is it possible that the differences in cell cycle genes (Fig 1D) reflect differences in phases of cell cycle?

We have conducted new sets of experiments to check if at least some of the differentially regulated genes implicated in proliferation (CD25), survival (CD127), migration (CCR2, CCR5, CD11c), activation/effector functions (PD-1, KLRD1) translated into differences in protein expression. CTV-labelled OT-I cells were transferred to mice subsequently challenged with the various *Lm*-Ova APLs, and CTV^{low} (dividing) OT-I cells analyzed in the spleens of immunized mice 3 days later (**new Figures 1E and S1F**). We confirmed that, like for their respective genes, CD25, CCR2, CCR5 and PD-1 expression were significantly different in N4- compared to A8- or T4-primed OT-I cells. However, we did not find significant differences in expression of CD127, CD11c or KLRD1 at this time point, possibly reflecting distinct kinetics of gene versus protein expression.

3. In Fig 2, the authors use Il2ramut/mut OT-I cells in adoptive transfer model, to investigate the requirement for IL-2-signals to form functional memory. This is a constitutive mutant, as a consequence it is difficult to distinguish between the impact of the lack of IL-2 signaling during T cell precursor differentiation, during priming, and during recall. The importance of IL-2 during priming for memory development has been analyzed by several groups before (some of which are cited by the authors).

As noted by this reviewer, the *Il2ra*$^{mut/mut}$ mouse is a constitutive mutant, preventing us from determining at which stage IL-2 signaling is needed. This is why we conducted the IL-2 signal rescue experiment reported in **Figure 2C.** Data suggested that rescuing IL-2 signals during priming and the primary response (day 0 to 6) is sufficient to enable competitive expansion of *Il2ra*$^{mut/mut}$ OT-I memory cells primed with a weak agonist (A8). These data are also consistent with the original results reported by the Bevan group (Williams et al., Nature 2006), although this

study did not make any connection to TCR signaling strength as the key mechanism regulating IL-2 dependency of memory cells to form fully functional memory cells.

In order to refine the timing of when IL-2 signals are needed to rescue *Il2ra$^{mut/mut}$* memory T cell competitive expansion, we conducted several experiments in which we treated mice immunized with a weak agonist (T4) with anti-IL-2/IL-2 complexes either i) for a shorter duration during priming (3 days) or ii) during recall infection (**revised Figure 2C and S2B**). Control groups included isotype-treated mice during primary and secondary challenge infections. Rescuing IL-2 signaling for only 3 days enabled more competitive expansion of *Lm*-T4-primed OT-I memory cells compared to isotype-treated group, and the rescue was comparable to that of the 6 day-treated group (**Figure 2C**). This suggested that robust IL-2 signaling most likely acted at very early stages of T cell priming to restore the proliferative competitiveness of T cells primed with weak agonists.

Interestingly, when anti-IL-2/IL-2 complexes were given during the recall response, WT and *Il2ra$^{mut/mut}$* OT-I memory cell expansion was fully restored, suggesting that providing robust IL-2 signals can also fully rescue impaired memory cell proliferative response. When T cells received strong TCR signals during priming (*Lm*-N4), robust IL-2 signals were not required for competitive recall expansion. However, when T cells were primed with weak TCR signals, strong IL-2 signals were required either during priming or recall response. Altogether these results confirm our findings that strong IL-2 signals are required in T cells primed with weak peptide agonists, to program them with a full proliferative potential at memory stage. The new data further indicate that IL-2 rescue signals can be provided either during early stage of priming or during the recall response.

We note that, in contrast to the Williams and Bevan study in 2006, providing IL-2 signals during the recall response fully restores the expansion of memory cell impaired in IL-2 signaling. In this paper, the authors use mixed BM chimera mice reconstituted with WT and *Il2ra$^{-/-}$* BM and GP_{33-41}/D^b-specific polyclonal memory $CD8^+$ T cells were monitored using corresponding tetramers. A relative fold expansion of WT and *Il2ra$^{-/-}$* memory cells before and 5 days after the challenge infection was calculated in isotype and IL-2/IL-2 Ab-treated mice. In contrast to our experiment, this set up does not allow to start with a known number of memory cells. In addition, many lymphocytes in the WT but not the knockout compartment will respond to the IL-2 treatment. We believe these important limitations in the experimental design could potentially confound interpretation of the results and account for the current discrepancy. These data have been incorporated in the revised manuscript and discussed accordingly.

4. In Figure 4, the authors use different models to investigate IL-2-dependent and -independent contributions to memory responses to natural epitopes. Based on the stabilization assay results (Fig 3F), it seems that the peptides with lower stability promote weaker responses (GP33-41). These results should be discussed, the conclusion of the paragraph should be modified accordingly.

Peptides with lower stability such as GP_{33-41} or Ova/A8 indeed induce a lower primary T cell expansion and an overall lower number of memory cells. Likewise, Ova/T4 also primes lower number of OT-I memory cells. In all of these experiments, induced memory cells require IL-2 signals to form memory cells that competitively expand, indicating that the critical parameter is the amount of TCR signals that naïve T cells get during priming. When an exact same number (1,000) of memory cells primed with strong (N4) or weak (T4) peptide agonists is purified and transferred to new recipient challenged the next day, their ability to expand is equivalent (**Figure 9D**). However, if IL-2 signaling is impaired (in *Il2ra$^{mut/mut}$* T cells), only the ones primed with weak peptide agonists fail to competitively expand. Thus, although numbers of induced memory cells are lower upon priming with weak peptide agonists, their intrinsic ability to expand competitively is not altered if IL-2 signaling is intact. We hope this clarifies our reasoning. Our answer to point 3 of Reviewer #1 also gets to this similar question. We have also tweaked the revised

manuscript to clarify our thinking.

5. In Figure 6, the ATAC-seq results show several differences in open chromatin loci among the different experimental conditions. The authors suggest that the are genes associated with OCRs induced by changes in TCR signals selectively targeted metabolic processes. Which metabolic process? How these processes affect the differentiation of memory T cell subsets? The mechanisms involved should be explored more precisely.
The data indicate that modulating both TCR and IL-2 signaling strength together led to remarkably more extensive genome-wide chromatin remodeling than only TCR or IL-2. Our GO pathway analysis further reveals that chromatin remodeling at genes involved in some more focused metabolic processes is occurring when only TCR signals are modulated. We have looked more specifically through these genes which involve unique lipid-modifying and glycan-modifying enzymes, cell-cycle and respiratory chain associated proteins. Proteins and enzymes related to such broad cellular processes are also found with remodeled OCRs in the memory cells primed under varying TCR and IL-2 signals, making it difficult to more directly assess specific functional outcomes in the memory cells induced with varying TCR signaling strengths. The prior figure 8B measured lipid uptake (BODIPY) by reactivated OT-I memory cells and showed lower uptake in the memory cells that were primed with weak (T4) compared to strong TCR signals. Lowering IL-2 signaling further diminished this uptake, consistent with the fact that modulating both TCR and/or IL-2 signals changed chromatin accessibility at these gene promoters. However, in order to streamline the take home message of the paper, and since were unable to make a better link to OCRs modulated by TCR signaling strength only, we have decided to remove these data from the revised paper and remain focused on stemness, cell cycle and calcium fluxes (**new Figures 7 and 9**, see also our detailed answer to Reviewer #2, point 4).

REVIEWERS' COMMENTS

Reviewer #1 (Remarks to the Author):

The author response and additional manuscript additions and experiments have sufficiently addressed my prior concerns.

Reviewer #2 Comments were mediated by reviewer #1 and considered suitably addressed.

(please request if you wish to see these comments in detail)

Reviewer #3 (Remarks to the Author):

The authors did a good job in revising their manuscript and performed several experiments addressing the main concerns raised by the referees.
